# A mobile phone application for malaria case-based reporting to advance malaria surveillance in Myanmar: a mixed methods evaluation

**DOI:** 10.1186/s12936-021-03701-6

**Published:** 2021-03-26

**Authors:** Win Han Oo, Win Htike, Julia C. Cutts, Kyawt Mon Win, Kaung Myat Thu, May Chan Oo, Galau Naw Hkawng, Paul A. Agius, Pyae Phyo Htoon, Nick Scott, Ellen Kearney, Clarissa Moreira, Alisa Pedrana, Mark Stoove, Aung Thi, Ye Win Aung, Min Min Thein, Kathryn Rosecrans, Kyi Tun Lwin, Aung Kyi Min, Freya J. I. Fowkes

**Affiliations:** 1 Burnet Institute, Melbourne, Australia; 2 Department of Medicine, Peter Doherty Institute for Infection and Immunity, University of Melbourne, Melbourne, Australia; 3 National Malaria Control Programme, Ministry of Health and Sports, Nay Pyi Taw, Myanmar; 4 Department of Epidemiology and Preventive Medicine, Department of Infectious Diseases, Monash University, Melbourne, Australia; 5 Save the Children, Yangon, Myanmar; 6 Melbourne School of Population and Global Health, University of Melbourne, Melbourne, Australia

**Keywords:** Malaria, Surveillance, Elimination, mHealth, Mobile phone applications

## Abstract

**Background:**

To achieve malaria elimination in the Greater Mekong Subregion, including Myanmar, it is necessary to ensure all malaria cases are detected, treated, and reported in a timely manner. Mobile phone-based applications for malaria reporting, case management, and surveillance implemented at a community-level may overcome reporting limitations associated with current paper-based reporting (PBR), but their effectiveness in this context is unknown.

**Methods:**

A mixed methods evaluation study was undertaken to determine the effectiveness of a national Malaria Case-Based Reporting (MCBR) mobile phone application in improving malaria case reporting compared to the existing PBR reporting system in Myanmar. Methods included secondary analysis of malaria case report data, questionnaires, focus group discussions and field observations of community volunteers, interviews and direct observations of malaria programme stakeholders, and cost analysis. Using a combination of these approaches the following areas were investigated: data quality and completeness, data access and usage, capacity for timely reporting, the acceptability, functionality, and ease of use of the application and facilitators and barriers to its use, and the relative cost of MCBR compared to the PBR system.

**Results:**

Compared to PBR, MCBR enabled more accurate and complete data to be reported in a much timelier manner, with 63% of MCBR users reporting they transmit rapid diagnostic test outcomes within 24 h, compared to 0% of PBR users. MCBR was favoured by integrated community malaria volunteers and their supervisors because of its efficiency. However, several technical and operational challenges associated with internet coverage, data transmission, and e-literacy were identified and stakeholders reported not being confident to rely solely on MCBR data for programmatic decision-making.

**Conclusions:**

Implementation of MCBR provided timely and accurate data for malaria surveillance. Findings from this evaluation study will enable the optimization of an application-based reporting system for malaria monitoring and surveillance in the Greater Mekong Subregion and advance systems to track progress towards, and certify, the achievement of malaria elimination targets.

**Supplementary Information:**

The online version contains supplementary material available at 10.1186/s12936-021-03701-6.

## Background

The emergence of drug resistant malaria in the Greater Mekong Subregion (GMS) has led all GMS countries (Myanmar, Lao People Democratic Republic, Cambodia, Thailand, Vietnam and Yunnan Province in China) to commit to eliminating malaria by 2030 [1, 2]. Between 2012 and 2015, GMS countries reduced malaria morbidity and mortality by 54 and 84% respectively [2]. Malaria programmes in many malaria-endemic areas in the region are now transitioning from sustained control to elimination stages and are classified as being in the “pre-elimination” phase, which involves reinforcement of reporting and surveillance systems [3]. To achieve elimination, accurate and complete notification of individual (not-aggregated) malaria cases within 24 h of diagnosis is considered essential to allow for timely and spatially-specific responses [4]. This is reflected in the 1-3-7 surveillance and response strategy pioneered by China and adopted, but not yet routinely implemented, by national malaria control programmes of other GMS countries, which specifies reporting of confirmed malaria cases within one day of diagnosis, investigation of specific cases within 3 days, and targeted foci control measures to prevent further transmission within 7 days [5].

In the GMS, with the exception of Yunnan Province in China, malaria surveillance systems at the village-level rely on paper-based reporting from a network of community health workers who provide malaria prevention, diagnosis, treatment and referral services to their communities in hard-to-reach and underserved areas [6, 7]. However, an evaluation of surveillance system performance in four GMS countries found that a system of paper-based reporting (PBR) and validation processes at each health administration level was inefficient and prone to delays in reporting as well as incomplete records and inaccessible data [8]. The National Strategic Plans of GMS countries acknowledge that routine malaria surveillance needs to be strengthened to ensure complete and timely reporting of all malaria cases to achieve malaria elimination [9–12].

Adopting a mobile health (mHealth) approach to malaria surveillance and public health practice may address current challenges in data accuracy and timely reporting in malaria surveillance [13]. SMS messaging and mobile applications for malaria reporting, case management, and surveillance have been pilot-tested in malaria programmes in Africa and Asia [14–19]. However, most of the studies published to-date report on applications that provide limited functionality within the broader surveillance system, were only tested in a confined geographical area, or were restricted to facility-based malaria services. The use of mobile phone applications for malaria case reporting by community-based volunteers within a national malaria surveillance system is yet to be reported [16].

In 2016 in Myanmar, Save the Children (SC), a non-governmental organization, led the development and roll-out of a mobile phone Malaria Case-Based Reporting (MCBR) application adapted from an application developed and pilot tested by Population Services International in Cambodia [19]. The open source MCBR application was originally developed for Android version 4.4 or later, using DHIS 2.23, and was later upgraded. It was distributed through the Aptoide application and the code can be requested and modified or reused for not-for-profit purposes. MCBR was designed to enable community health workers [known as integrated community malaria volunteers (ICMV)] to report accurate and complete malaria data in near real-time (within 1 day after getting the diagnosis), with the aim of improving 1-3-7 malaria surveillance strategy and increasing the effectiveness of malaria elimination programmes. Herein, a nationally representative mixed-methods evaluation of the effectiveness of the MCBR application for malaria reporting, its acceptability by stakeholders and the feasibility of it replacing the pre-existing PBR system for malaria surveillance is reported. MCBR application data quality and completeness, data access and usage, application functionality and ease of use, capacity for timely reporting, facilitators and barriers to use, and relative cost compared with the pre-existing PBR reporting system were analysed.

## Methods

### MCBR application and study setting

**Fig. 1:**
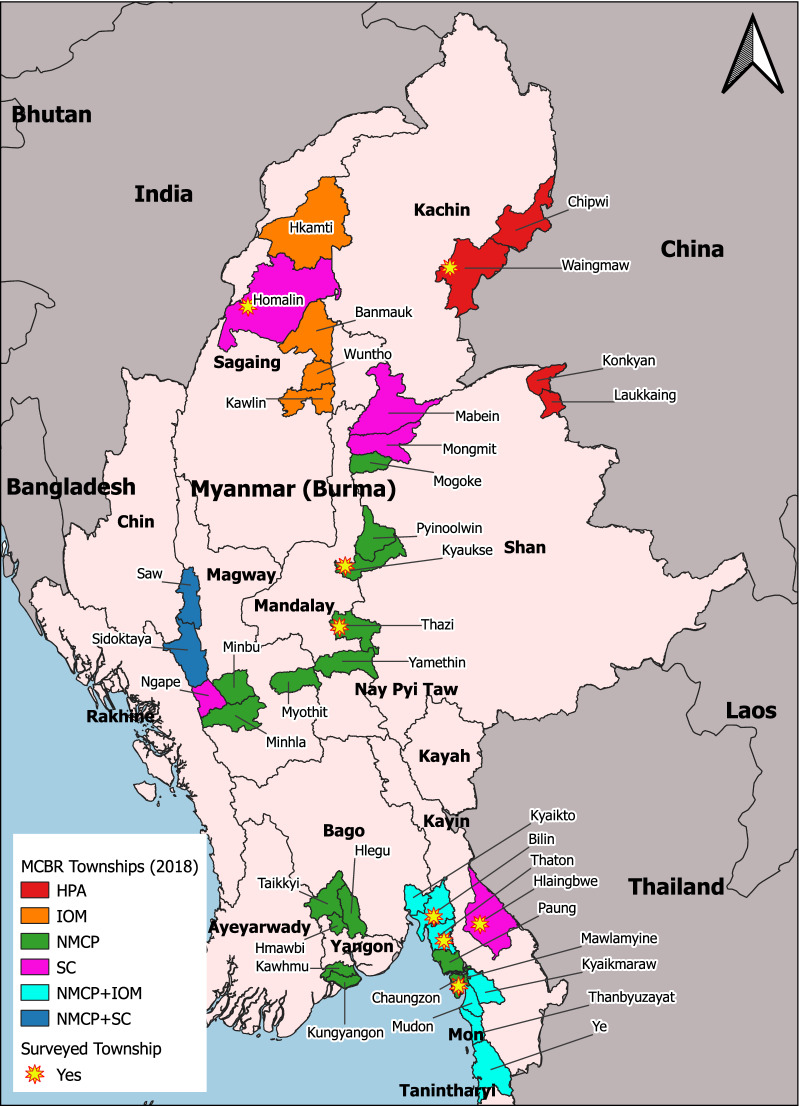
Map of townships using MCBR in 2018 and included in the evaluation

The data collected in the MCBR application aligns with indicators in the national malaria treatment guidelines and mirrors the standardized carbonless paper national malaria case register used by Myanmar National Malaria Control Programme (NMCP) and all implementing partners for PBR in Myanmar. MCBR captures 13 data elements relating to malaria rapid diagnostic tests (RDTs) (Additional file 1) and stock management, as well as additional job aid functionality, which provides operators with specific guidance on anti-malarial prescriptions and dosages and health communication messages (Additional file 2). MCBR data can be entered and stored offline but relies on an internet connection for automated transmission to a database housed on the District Health Information System 2 (DHIS2). As part of the MCBR roll-out, each participating ICMV was provided with a Samsung (Galaxy J1 or J2) mobile phone, with the MCBR application pre-loaded, a SIM card, and mobile credit. Following a 2017 pilot with 20 ICMVs in Mon State, malaria programme staff from NMCP and implementing partners underwent training to enable them to train ICMVs to use the application. The NMCP and implementing partners trained a total of 1527 ICMVs in 2018 who were operating in 1527 villages (of an estimated 20,000 villages serviced by ICMVs nationally) in 47 townships in 8 states/regions across Myanmar (see map of included study areas in Fig. 1 and list of villages, Additional file 3: Table S1). ICMVs were trained to report results from each malaria test using both the PBR and MCBR systems. Malaria data collected through MCBR is available to staff from all implementing partners and NMCP staff at different administrative levels through user accounts that allow them to log into the DHIS2 instance where data is sent. DHIS2 has built in audit logs and both the MCBR app and the DHIS2 instance require login with a username and password.

### Study design, data collection and analyses

The mixed methods evaluation was conducted in 2019–2020 and included quantitative secondary analysis of data collected using the PBR and MCBR systems as per the national malaria case register, relative cost data associated with each data collection system, and quantitative data from a questionnaire survey of ICMVs. These data were complimented with qualitative data from focus group discussions with ICMVs, key informant and in-depth interviews with malaria programme stakeholders, and field observations of ICMVs and data management staff. Study reporting adhered to the mHealth evidence reporting and assessment (mERA) [20] (Additional file 4) and STROBE checklists (Additional file 5).

Methods and data collection tools are detailed in Additional files 6 and 7, respectively. Briefly, NMCP and implementing partners provided secondary datasets containing data from individual malaria tests (RDTs) performed by ICMVs between 01 and 2018 and 31 December 2018, collected through the MCBR and PBR systems. Secondary data analysis was limited by the lack of a common identifier for the malaria test, or the patient, between the two reporting systems which precluded direct matching of the MCBR and PBR record for each malaria test. For test data from implementing partners, a common identifier for volunteers and information on volunteer training dates enabled us to exclude PBR test records (N = 21,272) that were performed by volunteers who had not yet been trained to use MCBR and were thus solely using the PBR system. These records were excluded because the aim was to only compare the completeness of records submitted by the cohort of volunteers who were using both systems concurrently. As exclusion of volunteers only using PBR was not possible for NMCP data (due to the lack of a common identifier for volunteers between the MCBR and PBR systems), secondary data analysis was performed separately to implementing partners. Data completeness was assessed by calculating the proportion of MCBR and PBR records that had complete fields (non-blank) for those data elements that were common to both systems and proportions were compared using Chi-squared tests (Stata version 15.0, StataCorp, Texas, USA). Median difference (in seconds) between finalization of data entered into the MCBR and synchronization was calculated from application meta-data available for Shan state.

A Myanmar language questionnaire was conducted to explore ICMVs’ views on the functionality and ease of use of both the MCBR and PBR system and capacity for timely reporting. To achieve a nationally representative sample, a two-stage cluster sampling approach was used to sample 163 ICMVs in nine townships across four implementing partner strata. Inverse proportional sampling weights were derived and applied, and variance estimation was corrected for the complex sampling approach employed. Participation rates for each township cluster and weighted counts are presented in  Additional file 3: Tables S2 and S3.

Fourteen focus group discussions were conducted with 83 ICMVs from Kachin, Kayin and Mon States, Mandalay, Sagaing and Yangon Regions and Nay Pyi Taw Union Territory of Myanmar townships (≥ 18 years; male: 38, female: 45), in groups of 5–6. A focus group discussion guide was used to explore ICMV opinions regarding the MCBR and PBR systems, with a focus on access, ease of use, functionality, and capacity for timely reporting applying qualitative descriptive approach. Applying the phenomenological approach, semi-structured interviews, including 12 in-depth interviews and two key informant interviews, were conducted with Ministry of Health and Sports and implementing partner staff to explore their experiences with data access and usage, policy making, and programme implementation. Interviewees were purposively recruited based on their roles managing malaria reporting data and to ensure representation from multiple organizational levels. Focus group discussions, in-depth and key informant interviews were audio recorded and transcribed verbatim, translated into English, organized, managed, and analysed thematically (deductive followed by inductive analysis) [21] in Nvivo (version 12). Two researchers immersed and coded data, and then discussed themes and subthemes to reach a consensus on the interpretation [22].

Researchers conducted field observations in 44 ICMV villages (6–7 ICMVs per township) and five field offices (where field managers and data monitoring and evaluation staff undertake malaria data management) using standardized observational field guides with checklists (Additional file 7). In ICMV observations, researchers recorded notes on village infrastructure and inspected malaria case registers, stock and health education record books, and ICMV’s notes for recording and reporting of malaria. In offices, researchers also inspected monthly and quarterly reports and MCBR records and documents relevant to the evaluation of malaria PBR or MCBR surveillance.

A programme experience approach was used to estimate the cost of development and roll out of the MCBR system compared to ongoing costs of running the PBR system. One-off and ongoing costs associated with each reporting system were itemized using cost data from expenditure reports. The cost of nation-wide implementation of the MCBR system was estimated based on the cost of the initial roll-out, minus any components related to the piloting process, multiplied by a geographical scaling factor calculated by dividing the estimated number of villages serviced by ICMVs nationally in Myanmar (~ 20,000) by the number of villages covered by the MCBR roll-out to end of 2019 (2488).

## Results

### Data quality and completeness of MCBR and PBR systems

In 2018, the implementing partner organizations received 41,040 malaria test records from 683 ICMVs through the MCBR system and 49,788 malaria test records from 615 ICMVs through the PBR system (Table 1). For the data from implementing partners, PBR records received from volunteers who were not yet trained to use MCBR were excluded, but this was not possible to do for data from NMCP. Thus, there was a larger excess of PBR records compared to MCBR records in the NMCP dataset. Over the same period the NMCP received 7882 malaria test records from 280 ICMV through MCBR and 52,214 malaria test records from 748 ICMV through PBR (Table 1). Across all organizations, MCBR and PBR records had a similar level of completeness. For date of test, patient sex, and RDT result, 100% of MCBR and PBR records were complete across all organizations. For pregnancy status 100% of MCBR and PBR records were complete for NMCP and 100% of MCBR records and 99.96% of PBR records were complete for implementing partners. For implementing partners and NMCP, the completeness of patient age, address, and the percentage of records with complete entries across five key fields was marginally higher for MCBR (100% for each field) compared to PBR (completion range 99.27–99.98%, p ≤ 0.040). For RDT-positive results (MCBR n = 311; PBR n = 403), 100% of MCBR records had a complete entry for the uncomplicated/complicated status of infection compared to 85% of PBR records (p < 0.001) and all PBR and MCBR records had a complete entry for anti-malarial provided in the case of RDT positive results. Of those RDT positive cases where there was evidence that referral was necessary according to the national guidelines (case was RDT positive and < 1 year old, pregnant or presenting with severe malaria symptoms), 94.11% (n = 48/51) of MCBR records had a complete entry for referral, compared to 33% (n = 1/3) of PBR records (p < 0.02) and within the NMCP all MCBR records (n = 8) and PBR records (n = 1) were complete, however total numbers were low.

**Table 1:** Completeness of MCBR data compared to PBR in implementing partner organizations and NMCP

	Implementing partner organizations			NMCP		
	MCBR	PBR	P value^a^	MCBR	PBR	P value^a^
Number of unique volunteers in 2018, N	683	615	–	280	748	–
Total number of records in 2018	41,040	49,788	–	7882	52,214	–
Overall completeness, N (%)						
Date of test	41,040 (100)	49,788 (100)	–	7882 (100)	52,214 (100)	–
Patient age	41,040 (100)	49,788 (100)	–	7882 (100)	52,186 (99.95)	0.04
Patient sex	41,040 (100)	49,788 (100)	–	7882 (100)	52,214 (100)	–
Patient address	41,040 (100)	49,780 (99.98)	0.008	7882 (100)	52,019 (99.63)	0.04
Pregnancy status (among females)	21,309/21,309 (100)	24,814/24,823 (99.96)	0.005	3971/3971 (100)	26,379/26,379 (100)	–
Pregnancy status (among females aged ≥ 15 years)	15,437/15,437 (100)	17,496/17,502 (99.97)	0.033	2950/2950 (100)	20,382/20,382 (100)	–
RDT result	41,040 (100)	49,788 (100)	–	7882 (100)	52,214 (100)	–
Records with 5 key data elements complete^b^, N (%)	41,040 (100)	49,780/49,788 (99.98)	0.01	7882 (100)	51,834/52,214 (99.27)	< 0.001
Total number of RDT positive results, N	283	292	–	28	111	–
Completeness for RDT positive results, N (%)						
Complicated/uncomplicated malaria status	283/283 (100)	249/292 (85.27)	< 0.001	28/28 (100)	111/111 (100)	–
Anti-malarial given	283/283 (100)	292/292 (100)	–	28/28 (100)	111/111 (100)	–
Referral recorded^c^	48/51 (94.11)	1/3 (33.33)	0.02	8/8 (100)	1/1 (100)	–

^a^Chi-squared test

^b^Data elements included in analysis were date of test, patient age, patient sex, patient address, and RDT result

^c^Where case is RDT positive AND < 1 year old, pregnant or presenting with severe malaria symptoms according to the guidelines for malaria diagnosis and treatment in Myanmar

### Functionality and ease of use of MCBR and PBR and adherence to standard guidelines

**Fig. 2:**
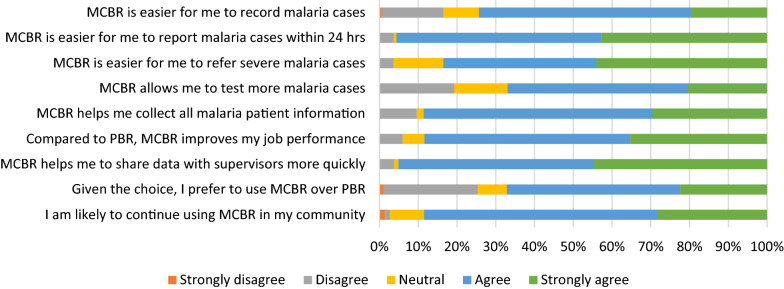
Opinions of ICMVs on malaria case management (MCBR versus PBR) and perceived value of MCBR Data was obtained from a representative survey of 163 ICMVs

Among 163 surveyed ICMVs (200 invited, participation rate 82%), the majority (80%) reported using both PBR and MCBR for all malaria patients, with the remainder using PBR for all patients and MCBR (in addition to PBR) for some of their patients (13%) or MCBR for all patients and PBR for some (7%) (Additional file 3: Table S4). In field observations, the time taken for reporting using the PBR and MCBR systems was similar (median 3 min per patient (IQR: PBR 7.5, MCBR 13). However, most ICMVs agreed or strongly agreed that compared to PBR, MCBR was easier for them to record malaria cases (75%), to report malaria cases within 24 h (96%), to refer severe malaria cases (84%) and would allow them to test more malaria cases (67%) (Fig. 2). Most ICMVs agreed that MCBR helped them share data with their supervisors more quickly than PBR (95%), they preferred to use MCBR (67%) and agreed they were likely to continue using it in their communities (88%) (Fig. 2).

Only 43% of the ICMVs reported using the MCBR application for malaria stock management. Of those, most found the MCBR application easier for reporting stock-outs and checking stock balance (81 and 66%, respectively) compared to PBR (Additional file 3: Table S5) although technical difficulties with the stock module were experienced by some ICMVs. Notably this stock module was dropped from the MCBR application in late 2019, after the completion of this study. The job aid component of the application, which provides ICMVs with specific guidance on anti-malarial prescriptions and dosages depending on the clinical information entered, as well as health communication messages, was identified as a benefit of MCBR in focus group discussions with the ICMVs and interviews with the stakeholders.

### Capacity for timely reporting

In interviews, stakeholders from NMCP and implementing partners indicated they were impressed with the timeliness of MCBR reporting. In questionnaires, most ICMVs (88%) reported that MCBR facilitated timely reporting and enabled them to report malaria cases at any time (Table 2), a strength confirmed in focus group discussions with ICMV. However, only about two-thirds (63%) of ICMVs reported that they submitted their MCBR reports within 24 h after each test, with less than a quarter (22%) submitting them monthly (Table 3). In contrast, 87% of ICMVs reported they submitted PBR records monthly, and no ICMVs reported submitting PBR records within 24 h (Table 3), a finding confirmed in focus group discussions and field observations of ICMVs. Monthly reporting of MCBR data may have been due to internet connectivity issues, with 20% of ICMVs reporting they having to travel outside their village to gain internet access which required time and sometimes out-of-pocket expenses for the ICMV (Table 3). Analysis of MCBR application meta-data (from Shan State only) found that, of test reports successfully transmitted to the server, the median time from finalization of data entry to synchronization was 40 s (IQR 21 s).

**Table 2:** Experience of MCBR use of 163 surveyed ICMVs

	N (%)^a^
Benefits of using MCBR	
Can communicate with supervisors easily	150 (91.7)
Can report at any time because it can be done with my mobile	145 (88.7)
Timely reporting	144 (88.2)
Does not require other tools as it only needs a mobile	116 (71.2)
Other^b^	22 (13.6)
Difficulties of using MCBR	
Poor internet connection	69 (42.0)
No electricity to use MCBR	47 (28.7)
Poor performance of mobile phone	40 (24.7)
Burmese font typing	29 (17.7)
Inaccessibility to internet	28 (16.9)
Not familiar to use mobile device	24 (14.5)
Records were not synced instantly	23 (14.3)
Small screen of mobile phone	20 (12.6)
Need to charge mobile frequently	19 (11.4)
Having issues with phone credit to enable consistent use of MCBR	17 (10.3)
Other^c^	24 (14.9)

^a^Denominators vary for each question due to the application of inverse proportional sampling weights

^b^Includes better knowledge sharing, saves time, easy data entry, better servicing, saves money, better communication, better stock management

^c^Includes not having enough time to do ICMV work, error in MCBR application, and error in stock management module of MCBR application

**Table 3:** Malaria reporting by 163 surveyed ICMV using PBR and MCBR

	PBR N (%)	MCBR N (%)
Frequency of report submission to respective organization		
Immediately after testing	0	102 (62.3)
Daily	0	1 (0.5)
Weekly	0	11 (6.7)
Fortnightly	0	2 (1.2)
Monthly	141 (86.6)	36 (21.9)
After monthly reporting deadline	1 (0.7)	1 (0.9)
Quarterly	21 (12.7)	0
As convenient	0	2 (1.2)
Other/unspecified	0	9 (5.4)
Logistics of submitting reports		
ICMV physically transports report to supervisor	114 (70.1)	na
ICMV submits report at meetings and training sessions	106 (64.8)	na
Supervisor collected report at ICMV’s residing village	46 (28.1)	na
ICMV passes report to someone	40 (24.5)	na
ICMV submits report from residing village using MCBR application	na	132 (80.8)
ICMV submits from a different location to residing village where there is internet access	na	33 (20.3)
Distance required to go to place with internet access for MCBR in miles, *mean (SE)*	na	3.0 (1.7)
Time required to go to place with internet access for MCBR in minutes, *mean (SE)*	na	45.9 (19.9)
Out-of-pocket expenses associated with reporting		
Yes	26 (16.1)	33 (20.2)
No	137 (83.9)	130 (79.8)
Amount of out-of-pocket expense in MMK, *mean (SE)*	4661 (3445)	2401 (691)
Cost category for out-of-pocket expenses		
Courier charges for sending PBR reports	26 (100.0)	na
Cost for filling out PBR	2 (6.0)	na
Mobile top-up charges	na	31 (95.5)
Maintenance cost for mobile phone	na	2 (7.3)
Travel cost to go to other places to get internet access	na	3 (10.6)

### Facilitators and barriers to using MCBR

Consistent with questionnaire findings, focus group discussions with ICMVs revealed their preference for using and continuing to use MCBR, as they indicated that malaria reporting with MCBR is “easy”, “quick”, “effective” and “convenient”. Most ICMVs were happy and proud to use this modern method for malaria reporting and indicated that using the MCBR application improved their social status and trustworthiness in their community. This increase in social standing in the community underpinned the ICMVs perception that MCBR would allow them to test more malaria cases.

***Participant 1***: In the past, I needed to persuade a sick student with snacks to get tested with a RDT because s/he was reluctant to do so. Now I explain to them (students) that I will test them, and do the reporting to the national and regional level offices instantly. I also let them observe how I do the reporting with my mobile phone. They are eager to see this modernized reporting technology and do not hesitate anymore to get tested with RDT.

***Participant 2***: People think we are running high technology because we send our report to the malaria team by mobile phone, and it wasn’t so in previous years when we were sending our report by hand-written papers. They trust us more because we entered their information into the mobile phone and sent it (to the malaria team) in front of them.

***Participant 1***: I told them that all of their names (and information) could instantly reach the [township level office], [regional level office] and [national level office]. They trust us, comparing with the previous years.

***Participant 2***: … They know that I am using this mobile phone for our work and I am truly connected with respective Ministry. So, they trust me more.”

(Discussion with female ICMVs from Mandalay Region)

ICMVs liked that reporting only required a mobile phone and no other tools, although some expressed concern about loss or damage to phones. ICMVs also recognized that MCBR saved time associated with physical transportation of PBR, especially for those living in areas where transportation is difficult in the rainy season. Using MCBR was also more convenient and efficient for ICMV supervisors, who reported they could spare the human resources, time, and money which was otherwise required for collecting and managing PBR reports. ICMVs said this extra time enabled more time to be spent monitoring and analysing reported data, but this was not independently verified.

ICMV focus group discussion participants reported a lack of electricity and internet connectivity as a major barrier to using MCBR (also reported by 29 and 42% of surveyed ICMVs, respectively, Table 2). Problems with electricity supply and internet connectivity was also directly observed in 5/18 villages in field observations. In focus group discussions, almost all ICMVs reported experiencing data synchronization issues at least once and MCBR application errors are detailed in Additional file 3: Table S6. Sometimes, when ICMVs thought records had not been transmitted, they repeatedly re-sent case records resulting in duplicated records which supervisors or data managers had to manually remove from the DHIS2 server. ICMVs also reported that records occasionally disappeared from their mobile phones. Additional technological barriers to using MCBR identified in focus groups, survey responses and field observations, included poor performance of the handset, low e-literacy, and technical issues with the MCBR application itself (e.g. auto-logout and errors in stock management module). To overcome e-literacy and technological difficulties, ICMVs sought help from peers, their children who are familiar with mobile devices, or their supervisors. Although 91% of ICMVs reported they received adequate training for use of MCBR application, they also indicated a desire for additional training to improve their e-literacy (Additional file 3: Table S7).

Interviews with the stakeholders echoed the challenges reported by the ICMVs.

*“The first condition is that all villages must be connected and covered with internet network. Actually, the network coverage must cover the whole country. Not only the network coverage, we should provide technical support to volunteers effectively. We must provide all necessary equipment that is related to the availability of budget to volunteers and staff. We must replace the lost or damaged phone, or repair them in time. I mean logistic support. Furthermore, financial support is needed. We must provide phone credit. If a volunteer has to travel to a location that has internet network coverage for reporting, then we must provide travel cost for him/her. So many things to support. And the electricity. They can’t report (via MCBR) if the village has no electricity. We need to provide such things. The application must be stable as well. We also need to assess the usefulness and perfection of DHIS2 that we are recommended to use.”* (Interview with a Field Supervisor from an Implementation Partner).

### Data access and stakeholder utilization

Data collected by ICMVs was transmitted to the township, regional and national levels either electronically (MCBR) or delivered via ICMV supervisors (PBR) (diagram in Additional file 8 outlines the flow of malaria test data). At the township level, MCBR data was accessed monthly in the DHIS2 database by staff who monitored malaria epidemiology and organizational performance and produced summary reports which were sent to regional offices. Township-level staff, as well as state and regional level NMCP staff, checked the MCBR data in the DHIS2 database against PBR data in an Access database and corrected errors where possible.

During in-depth interviews programme staff reported they could only retrieve 50–75% of records reported through PBR data from the MCBR DHIS2 database, undermining the perceived reliability of MCBR data. Whilst an excess of PBR records compared to MCBR records (19.3%) was confirmed in secondary data analysis of test results from implementing partners (Table 1), the magnitude of the discrepancy was not as large as that estimated by these stakeholders.

Field office staff reported in interviews that correcting errors in the MCBR dataset in DHIS2 was difficult because searching by ICMV name or identification number was not possible. Implementing partner stakeholders identified in interviews that missing or inconsistent village codes or coordinates prevented MCBR data visualization on the DHIS2 dashboard map. This was exacerbated by ICMVs not being able to specifically attribute patient data to specific villages if ICMVs provided services outside the ICMV’s assigned village. Furthermore, implementing partner stakeholders explained in interviews that because MCBR has only recently been implemented, there is insufficient accumulated malaria case data to facilitate temporal analyses of malaria trends.

*“For analysis, they taught us how to produce dashboard and graphs (in DHIS2) during the training. But we haven’t tried it before because we only manage a township the data is not sufficient enough to present in the dashboard or by graphs. And we only started using MCBR last year. It has been just a year that we use it. So, there is not much data.”* (Interview with a Field Supervisor from an Implementation Partner).

The NMCP regional focal person reports information on malaria generated from the MCBR and PBR data to their higher-level state or regional staff such as State/Regional Health Director and Deputy Directors (see flow diagram of malaria test data in Additional file 8). However, field observations in the national NMCP office and interviews revealed that the NMCP continued to rely on PBR data for programme management, and MCBR data was not directly accessed by national level staff. In contrast, at the head offices of the implementing partners, MCBR data was compiled, managed, analysed, and used for donor reporting to visualize malaria trends including identification of outbreaks, and calculation of key malaria programme indicators.

*“We tend to look at the, try to look at the (malaria indicators) in different areas. So, we can do that, extracting the MCBR data or if we’re able to clean the data that’s going into the Access database, we can use that as well, too. And pair it with the population data to look at (malaria indicators) and we’ve done that in some cases, especially when there’s like unusual things happen, I can validate. This past year, there’s higher than expected seasonal malaria. So, we started looking very closely. And that’s when having this sort of very fine, detailed data can be important.”* (Interview with a Programme Manager from an Implementation Partner).

During the FGDs and interviews, ICMVs and stakeholders acknowledged there is no direct communication mechanism built-in to the MCBR application to provide feedback to the ICMVs regarding the delivery status of reported data, the number of tests performed and patient records reported, data quality issues like data errors and missing data; and technological problems encountered by the ICMVs.

“I want feedback from my supervisors whether they have received my report (sent through the MCBR) or not. If they receive my report, I would like to know how many cases they do receive. They don’t need to make a phone call to me for the report. They can just provide feedback through something like (Facebook) Messenger. I want to get a message how many cases were reported within a period. I want that kind of feedback form them (supervisors).”

“I want a corrective or supportive feedback from the supervisors based on the analysis of data that we submitted.”

“I need encouragement and psychological support (about the report) from the supervisors.”

(Discussion with female ICMVs from Kachin State)

*“There is no function in the MCBR to provide feedback to the volunteers. There could be data errors, missing data and delayed reporting from the volunteers. The volunteers might also have questions to ask to supervisors and issue popped up to be reported to the supervisors. In these cases, the MCBR couldn’t fulfil such requirements.”* (Interview with a Team Leader from NMCP).

Under the MCBR and PBR systems, feedback relies on supervisors reviewing data and communicating with ICMVs in person, by telephone call, or through Viber instant messaging.

*“The Viber group just serve as a counterchecking mechanism. If there is an issue, the ICMVs can take a photo of it and show that to us so we can respond. And we can provide feedback via Viber immediately. I mean for things like data errors. We use Viber for these purposes.”* (Interview with a Regional Officer from NMCP).

The stakeholders pointed out the requirement of establishing a good feedback system in the MCBR and expected to have such a feature in the next iterations of the application.

*“The ICMVs would like to be appreciated. Let’s say, they want to individually hear and know such things as how many people they have tested, how many of the records have been sent, and how many of them are malaria positive, how much the positivity has decreased. It will motivate them more. If not, they will not know how far they have gone and it will make them a little bit bad.”* (Interview with a Programme Manager from an Implementation Partner).

“***Interviewer***: Do you think we can add some feature in the (MCBR) application to provide feedback to the ICMVs? I don’t mean like the daily feedback but maybe monthly or something like this.

***Participant***: *Yeah, I think that’s an idea. I mean, the MCBR does have just sort of a summary of number of tests, and number of cases and things like that. But I think certainly there could be, there could be more and I’m open to ideas about what would be valuable from a volunteer’s perspective.”*

(Interview with a Programme Manager from an Implementation Partner)

Perspectives on the transition from PBR to MCBR were explored in focus group discussions and interviews. Although participants’ responses were not universal, most ICMVs were anticipating the value in transitioning from PBR to MCBR reporting in the malaria programme. Some stakeholders interviewed perceived the PBR could be replaced with MCBR in the near future, but others identified current barriers to transitioning to the MCBR, including issues around appropriate training for ICMVs.

“***Participant 1***: I suppose so (the MCBR can replace PBR) because it (MCBR) is easy and fast, I think. I hope that it (PBR) will be replaced.

***Participant 2***: I do not think so. There is no really educated and graduated person among the (ICMVs), some of the villagers are humble and they usually only know how to turn on the radio, and some cannot even distinguish between A and B, and, maybe, do not know them at all. I think this mobile (MCBR) will not last forever.

***Participant 3***: As for me, the project has planned to do so and so to keep going, is someone is facing difficulties then provides him/her more knowledge and more training. Hopefully, this will work because we have to keep going.

***Participant 4***: *I think, in this ever-improving computer age, only mobile phones will be used in the future.”*

(Discussion with male ICMVs from Sagaing Region)

*“I don’t ever think that we’re going to have 100 % of all volunteers all the time using the app, but I think we could get a 95 or 99. … I’ll say that Myanmar has challenges in terms of conflict areas, in terms of areas that are still very remote. And so, I think, you know, some of those things will take a little while, but overall, yes, I think it’s possible (that the PBR can be replaced totally with the MCBR). One of the reasons we wanted to have this iteration is to see if we’re there yet. We might not be there yet; I still think it’s possible. And I hope we’re close.”* (Interview with a Programme Manager from an Implementation Partner).

*“But in such an electronic system as the MCBR, if all the barriers we have discussed have been solved, the MCBR will become a surveillance system better than the Carbonless system.”* (Interview with a Team leader from NMCP).

### Cost analysis

The total cost, including both implementation plus ongoing annual costs, of the MCBR programme to 1527 ICMVs included in the roll-out over one-year was US$0.46 million, compared to US$0.29 million for the PBR programme (Table 4; Additional file 3: Tables S8–S10). The cost of expanding the MCBR to 20,000 ICMVs nationwide over a 3-year period was calculated to be US$14.13 million (reflecting the longevity of capital items purchased in the first year and associated reductions in cost per ICMV), compared to US$11.41 million for the PBR system (Table 4).

**Table 4:** One-off plus ongoing costs for implementation of MCBR and PBR for an ICMV and for reporting of a testing (in a year with 1527 ICMVs and 20,000 ICMVs, and 3 years implementation with 20,000 ICMVs)

Items	MCBR overall	PBR overall	Difference
Cost per ICMV for 1 year (for1527 ICMVs)	$301.55	$190.19	$111.36
Cost per ICMV for 1 year (for 20,000 ICMVs)	$272.01	$190.19	$81.82
Cost per ICMV for 3 years (for 20,000 ICMVs)	$235.55	$190.19	$45.36
Cost per test reported per year from1527 ICMVs	$9.41	$2.72	$6.69
Cost per test reported per year nationwide from 20,000 ICMVs	$2.55	$1.78	$0.77
Cost per test reported per 3 years nationwide from 20,000 ICMVs	$2.20	$1.78	$0.42
Total cost for 1 year (for 1527 ICMV)	$460462.86	$290418.28	$170044.58
Total cost for 1 year (for 20,000 ICMV)	$5440272.57	$3803775.75	$1636496.82
Total cost for 3 year (for 20,000 ICMV)	$14132978.52	$11411327.26	$2721651.26
Contribution by cost category			
HR cost (% contributed to the total cost)	32.72%	68.81	− 36.09%
Staff travel (% contributed to the total cost)	3.10%	7.16	− 4.06%
Phone credit + ICMV travel cost for MCBR reporting	26.00%	0.14%	25.86%
ICMV trainings and meetings	38.19%	23.89%	14.30%

All costs were calculated in United States Dollars (US$), with expenditure in Myanmar Kyats converted into US$ using the United Nations exchange rate applied in 2018. Overall costs represent the average cost across NMCP and three implementing partners

Thus, scale-up of the MCBR programme would be associated with a reduction in cost per ICMV, due to the sharing of some infrastructure, whereas the cost per ICMV for the PBR programme remains the same

## Discussion and conclusion

Surveillance of malaria using mobile phones has the potential to track and accelerate progress towards malaria elimination. Analysis of those malaria test records that were able to be retrieved found that MCBR led to more complete reporting of data compared to PBR. Surveys and focus group discussions with ICMVs indicated that MCBR enabled malaria case data to be shared in a timelier manner compared to PBR. MCBR was favoured by ICMVs and their supervisors, mainly due to its improved efficiency, its perceived ability to increase malaria testing and improve the status of ICMVs in their community. However, several technical, infrastructure and operational challenges associated with internet coverage, data synchronization, and e-literacy of ICMVs impacted MCBR’s capacity for real-time reporting. Additionally, stakeholders at different levels were not yet sufficiently confident to rely on MCBR data alone for programmatic decision-making because it was perceived to be incomplete.

Of the data that could be retrieved the quality of data collected and managed through the MCBR was at least as good as the PBR system but offered clear advantages in terms of timeliness and efficiency. Importantly, according to respondents, the direct data entry by ICMVs into the MCBR system improved the timely notification of malaria cases, with two-thirds of MCBR users reporting they notified RDT outcomes within 24 h (the first component of the 1-3-7 strategy for malaria elimination), compared to no PBR users. However, at the township-level, MCBR data is only accessed monthly and lacked a timely alert system to prompt action on malaria cases, meaning that timely malaria investigation and responses for some cases may not occur at township levels using the MCBR system. Other web-based systems previously evaluated in the region, including DHIS2, have incorporated an SMS alert system, which alerts the responsible focal person once a malaria case is reported [14, 23]. An e-mail alert system has been integrated in Malaria Case-Based Reporting and Surveillance (MCBRS), the next iteration of MCBR, in order to expedite timely notification of malaria cases to township or regional malaria focal persons of NMCP. However, the MCBRS could be extended to include prompts for ICMVs to follow-up malaria patients to ensure treatment compliance and to monitor any ongoing symptoms and emerging drug resistance, as well as to support the case and foci investigation of the 1-3-7 strategy for malaria elimination. As of February 2021, there were more than 3000 MCBRS users in more than 100 townships, with plans for further expansion. From 2021 onward, Myanmar NMCP plans to use the MCBRS application after pilot testing, conducting a readiness assessment of the NMCP server to host MCBRS data and finalizing a transition readiness plan of MCBRS in NMCP server. Myanmar NMCP also plans to provide nationwide MCBRS training to its State/Regional teams and ICMVs in a cascading approach, aiming to provide real time reporting from ICMVs throughout the country in 2023.

Despite the clear benefit of the MCBR application for data completeness and timely reporting, technical and operational issues, particularly around data synchronization and retrieval, impacted epidemiological reporting of MCBR data, and PBR data continues to be relied upon for evidence-based decision-making in malaria programmes in Myanmar.

Importantly, limitations around data visualization due to misclassification of the geographical origin of cases outside of an ICMV’s village prohibited adequate microstratification of case data at the village-level and prevented the use of MCBR data to inform the design of site-specific malaria interventions and programmes. NMCP and implementing partner stakeholders continue to consider PBR data more reliable than MCBR data, largely due to perceived concerns regarding missing data and an excess of PBR records relative to MCBR records, duplicated records in the MCBR system, and perceived system instabilities, including application bugs that caused syncing issues of MCBR in its early years of establishment. A consistent issue reported, which may explain a discrepant number of MCBR relative to PBR records, and underpinned data access and utilization barriers in offices, was internet connectivity and lack of electricity in rural areas. Mitigation of internet and electricity issues are beyond the direct control of the Ministry of Health and Sports and implementing partners, but reliable and sustainable connections are essential if the MCBR application is to be expanded nationwide or regionally. An assessment of surveillance systems conducted in 2015–2016 found that all GMS countries investigated (Cambodia, Laos, Myanmar, and Vietnam) had systems that suffered from lengthy paper-based record keeping and validation processes at each health administration level and at that time none of the countries had systems that could support rapid case-based reporting [8]. Whilst mobile phone applications and SMS alert systems for malaria surveillance have been pilot-tested in Thailand and Cambodia, to-date there have been no comprehensive evaluations of these interventions published, particularly with respect to qualitative findings on user acceptability and data access and usage [14, 19, 24, 25]. However, it is possible that the technical and operational issues identified in this evaluation, as well as the likely benefits of a system supporting rapid reporting of malaria cases, may be shared by other countries in the region.

One issue that can be addressed by MCBR implementers is the promotion of MCBR use, improving e-literacy and providing the additional training to support future rollouts and ongoing maintenance and fidelity of MCBR programmes. Stakeholders indicated a commitment to transitioning to a system whereby the planning, implementation and evaluation of malaria programmes relies exclusively on data from the MCBR system. However, for this to be achieved, the MCBR system and its supporting infrastructure must be improved and stabilized, so that stakeholders have complete trust in MCBR data reported by ICMVs. Data synchronization issues may be mitigated in areas where connectivity is known to be problematic by developing modified field protocols including routine schedules for travel to locations with good internet connectivity at least once a week to enable data synchronization.

A strength of this study was the representativeness of data used in secondary data analyses. The ICMV analyses were based on a representative multi-stage cluster sample, yielding findings which are representative of all MCBR implementation townships. Qualitative data collection was conducted with a broad range of participants from frontline MCBR application users as well as from data users at the township, state/regional and national levels yielding sufficient coverage of the salient ideologies (attitudes, beliefs, thoughts), behaviours and processes relating to the MCBR roll-out. Although this operational research was conducted in late 2019 and early 2020, prior to the evolution of the MCBR application into MCBRS, the key findings will be useful in optimizing mHealth reporting systems into the malaria elimination programme in Myanmar and the GMS more broadly.

A limitation was that in secondary data analyses it was not possible to match the MCBR and PBR record for each malaria case because there was no common identifier for the malaria test, or the patient, between the two reporting systems. Therefore, investigation of the considerable discrepancy in the number of records between MCBR and PBR was limited. The inability to match MCBR and PBR records is unlikely to have affect the conclusion that completeness of those MCBR records received was as high as those received through PBR. Furthermore, while secondary data analysis did not evaluate quality or adherence to the National Malaria Treatment Guideline by MCBR users per se, the quality of service provision was measured through proxy indicators such as effective stock management, malaria diagnosis, treatment, referral and notification of a malaria case within 24 h after diagnosis. In this evaluation, which was designed after the roll-out of the application, it was not possible to directly measure the timeliness of reporting using MCBR compared to PBR. Evaluation of the capacity for timely reporting was based on a combination of the reports of timeliness according to users and an estimation of time from finalization of data in the MCBR to synchronization, which was limited to malaria tests in a single state and did not address the issue of malaria test data that never reached the server. An additional limitation was that the stock module, favoured by ICMVs who reported using it, was dropped from the MCBR application in late 2019 and early 2020 during the transition to the updated MCBRS application. Incorporation of an improved stock module into MCBRS may improve supply chain of essential ICMV commodities and ensure the continued access of the community to malaria testing and treatment.

MCBR cost ~ 23% more compared to PBR (for 3 years and 20,000 ICMVs), with additional costs mainly attributable to one-off expenses (mobile phones, training) and costs not affected by increasing number of MCBR users (e.g. software development, server administration) which will reflect longer-term savings of MCBR. In the long-term, mobile phone applications for reporting malaria testing and cases could be an affordable, highly acceptable cornerstone of malaria monitoring and surveillance in the GMS, tracking progress towards, and World Health Organization certification of, malaria elimination targets (which requires a central computerized database of a national register of geospatially-specific malaria cases and foci) [26]. The MCBR proved to be a good starting point for transitioning malaria programmes from paper-based reporting to an electronic reporting system and has the capacity to improve malaria service provision in the community as well as providing timely, accurate data for malaria surveillance. Nevertheless, this study identified several technical and operational issues which need to be resolved to advance the MCBR system, and stakeholder’s confidence in it, and to inform the ongoing design of mHealth applications and their national or regional rollouts. In this COVID-19 era, the advantages of mHealth for malaria reporting has been highlighted; travel restrictions have impacted the flow of PBR, but not MCBR, in Myanmar. This highlights that the transition to mHealth for malaria reporting needs to be prioritized in order to meet the reporting needs of national programmes during a pandemic.

## Supplementary Information

**Additional file 1.** Carbonless register.

**Additional file 2.** Screenshots of MCBR application.

**Additional file 3.** Additional tables.

**Additional file 4.** Completed mERA reporting checklist.

**Additional file 5.** Completed STROBE checklist.

**Additional file 6.** Additional methods.

**Additional file 7.** Data collection tools.

**Additional file 8.** Diagram of malaria case data flow.

## Data Availability

With the exception of cost data, data cannot be made publicly available because it would breach compliance with the ethical framework of the Institutional Review Board 1, Myanmar Ministry of Health and Sports. De-identified individual participant data will be available after publication from the data custodian(s) to applicants who provide a sound proposal to The Institutional Review Board (1), Office Number (4), Ministry of Health and Sports, Nay Pyi Taw, Myanmar; (+95) 067 3431071 info@mohs.gov.mm contingent of their approval.

## Abbreviations

DHIS2: District Health Information Software 2
FGD: Focus Group Discussion
ICMV: Integrated community malaria volunteer
MCBR: Malaria Case-Based Reporting
MCBRS: Malaria Case-Based Reporting and Surveillance
mHealth: Mobile health
NMCP: National Malaria Control Programme
PBR: Paper-based reporting
RDT: Rapid diagnostic test
