# Supplementary material for: A mobile phone application for malaria case-based reporting to advance malaria surveillance in Myanmar: a mixed methods evaluation
Source: Malar J. 2021 Mar 26;20:167. doi: 10.1186/s12936-021-03701-6 (PMC7995396; doi:10.1186/s12936-021-03701-6)
Supplement: Supplementary file 2 — Additional file 2. Screenshots of MCBR application. [file 12936_2021_3701_MOESM2_ESM.pdf]

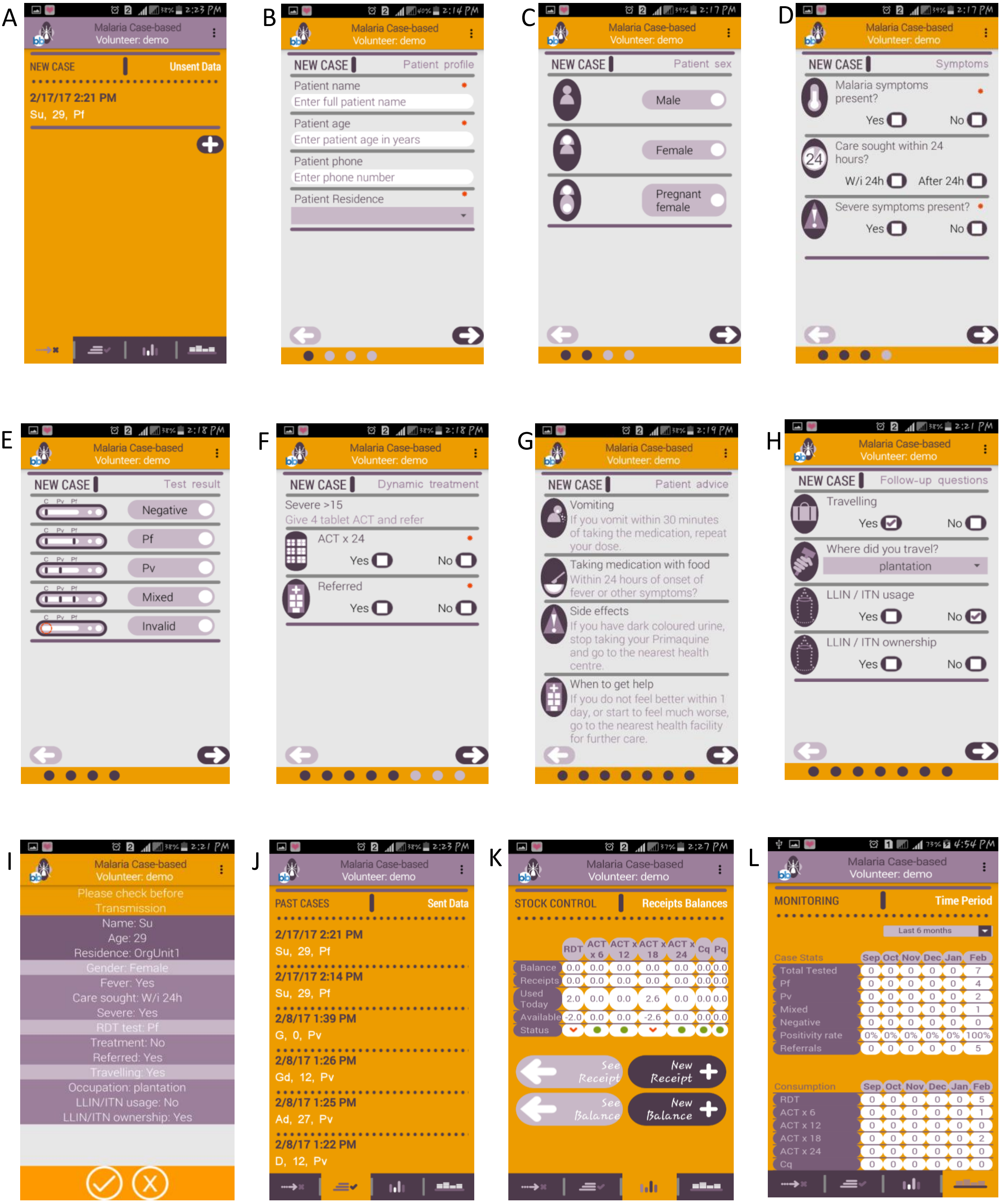
A
Malaria Case-based
Volunteer: demo
NEW CASE
Unsent Data
2/17/17 2:21 PM
Su, 29, Pf
B
Malaria Case-based
Volunteer: demo
NEW CASE
Patient profile
Patient name
Enter full patient name
Patient age
Enter patient age in years
Patient phone
Enter phone number
Patient Residence
C
Malaria Case-based
Volunteer: demo
NEW CASE
Patient sex
Male
Female
Pregnant female
D
Malaria Case-based
Volunteer: demo
NEW CASE
Symptoms
Malaria symptoms present?
Yes
No
Care sought within 24 hours?
24
W/i 24h
After 24h
Severe symptoms present?
Yes
No
E
Malaria Case-based
Volunteer: demo
NEW CASE
Test result
Negative
Pf
Pv
Mixed
Invalid
F
Malaria Case-based
Volunteer: demo
NEW CASE
Dynamic treatment
Severe >15
Give 4 tablet ACT and refer
ACT x 24
Yes
No
Referred
Yes
No
G
Malaria Case-based
Volunteer: demo
NEW CASE
Patient advice
Vomiting
If you vomit within 30 minutes of taking the medication, repeat your dose.
Taking medication with food
Within 24 hours of onset of fever or other symptoms?
Side effects
If you have dark coloured urine, stop taking your Primaquine and go to the nearest health centre.
When to get help
If you do not feel better within 1 day, or start to feel much worse, go to the nearest health facility for further care.
H
Malaria Case-based
Volunteer: demo
NEW CASE
Follow-up questions
Travelling
Yes
No
Where did you travel?
plantation
LLIN / ITN usage
Yes
No
LLIN / ITN ownership
Yes
No
I
Malaria Case-based
Volunteer: demo
Please check before Transmission
Name: Su
Age: 29
Residence: OrgUnit1
Gender: Female
Fever: Yes
Care sought: W/i 24h
Severe: Yes
RDT test: Pf
Treatment: No
Referred: Yes
Travelling: Yes
Occupation: plantation
LLIN/ITN usage: No
LLIN/ITN ownership: Yes
J
Malaria Case-based
Volunteer: demo
PAST CASES
Sent Data
2/17/17 2:21 PM
Su, 29, Pf
2/17/17 2:14 PM
Su, 29, Pf
2/8/17 1:39 PM
G, 0, Pv
2/8/17 1:26 PM
Gd, 12, Pv
2/8/17 1:25 PM
Ad, 27, Pv
2/8/17 1:22 PM
D, 12, Pv
K
Malaria Case-based
Volunteer: demo
STOCK CONTROL
Receipts Balances
RDT
ACT x 6
ACT x 12
ACT x 18
ACT x 24
Cq
Pq
Balance
Receipts
Used Today
Available
Status
See Receipt
New Receipt
See Balance
New Balance
L
Malaria Case-based
Volunteer: demo
MONITORING
Time Period
Last 6 months
Case Stats
Sep Oct Nov Dec Jan Feb
Total Tested
Pf
Pv
Mixed
Negative
Positivity rate
Referrals
Consumption
RDT
ACT x 6
ACT x 12
ACT x 18
ACT x 24
Cq
