# Supplementary material for: A mobile phone application for malaria case-based reporting to advance malaria surveillance in Myanmar: a mixed methods evaluation
Source: Malar J. 2021 Mar 26;20:167. doi: 10.1186/s12936-021-03701-6 (PMC7995396; doi:10.1186/s12936-021-03701-6)
Supplement: Supplementary file 3 — Additional file 3. Additional tables. [file 12936_2021_3701_MOESM3_ESM.docx]

**Table S1:** Township wise number of MCBR deployed volunteers by Implementing Partners (IPs) (as of April, 2019)

| **#** | **Implementing Partner** | **State/Region** | **Township** | **Total number of ICMV using MCBR+PBR** |
| --- | --- | --- | --- | --- |
| 1 | IOM | Mon | Bilin | 67 |
| 2 | IOM | Mon | Kyaikmaraw | 10 |
| 3 | IOM | Mon | Kyaikto | 50 |
| 4 | IOM | Mon | Mudon | 5 |
| 5 | IOM | Mon | Thanbyuzayat | 21 |
| 6 | IOM | Mon | Thaton | 34 |
| 7 | IOM | Mon | Ye | 25 |
| 8 | IOM | Sagaing | Banmauk | 21 |
| 9 | IOM | Sagaing | Hkamti | 32 |
| 10 | IOM | Sagaing | Kawlin | 20 |
| 11 | IOM | Sagaing | Wuntho | 9 |
|  | **IOM total** |  | **11** | **294** |
| 12 | HPA | Kachin | Chipwi | 10 |
| 13 | HPA | Kachin | Konkyan | 4 |
| 14 | HPA | Kachin | Laukkaing | 27 |
| 15 | HPA | Kachin | Waingmaw | 31 |
|  | **HPA total** |  | **4** | **72** |
| 16 | SC | Kayin | Hlaingbwe | 28 |
| 17 | SC | Magway | Ngape | 30 |
| 18 | SC | Magway | Saw | 79 |
| 19 | SC | Magway | Sidoktaya | 22 |
| 20 | SC | Sagaing | Homalin | 157 |
| 21 | SC | Shan (North) | Mongmit | 20 |
| 22 | SC | Shan (North) | Mabein | 24 |
|  | **SCSR total** |  | **7** | **360** |
| 23 | NMCP | Mon | Bilin | 30 |
| 24 | NMCP | Mon | Chaungzon | 27 |
| 25 | NMCP | Mon | Kyaikmaraw | 25 |
| 26 | NMCP | Mon | Kyaikto | 32 |
| 27 | NMCP | Mon | Mawlamyine | 30 |
| 28 | NMCP | Mon | Mudon | 30 |
| 29 | NMCP | Mon | Paung | 28 |
| 30 | NMCP | Mon | Thanbyuzayat | 27 |
| 31 | NMCP | Mon | Thaton | 31 |
| 32 | NMCP | Mon | Ye | 31 |
| 33 | NMCP | Mandalay | Pyinoolwin | 50 |
| 34 | NMCP | Mandalay | Yamethin | 45 |
| 35 | NMCP | Mandalay | Kyaukse | 20 |
| 36 | NMCP | Mandalay | Mogoke | 50 |
| 37 | NMCP | Mandalay | Thazi | 30 |
| 38 | NMCP | Yangon | Kungyangon | 39 |
| 39 | NMCP | Yangon | Hmawbi | 20 |
| 40 | NMCP | Yangon | Taikkyi | 40 |
| 41 | NMCP | Yangon | Hlegu | 36 |
| 42 | NMCP | Yangon | Kawhmu | 40 |
| 43 | NMCP | Magway | Minbu | 30 |
| 44 | NMCP | Magway | Minhla | 25 |
| 45 | NMCP | Magway | Myothit | 25 |
| 46 | NMCP | Magway | Sidoktaya | 35 |
| 47 | NMCP | Magway | Saw | 25 |
|  | **NMCP total** |  | **25** | **801** |
|  | **Grand total** |  | **47** | **1527** |

Table S2. Survey sample size and participation rate in each cluster

| **Organization** | **Selected township (cluster)** | **Targeted cluster size^a^** | **Eligible ICMVs** | **Participation rate (%)** |
| --- | --- | --- | --- | --- |
| IOM | Bilin | 30 | 25 | 83.3 |
|  | Thaton | 30 | 23 | 76.7 |
| HPA | Chipwi and Waingmaw | 23 | 20 | 87.0 |
| SC-SR | Hlaingbwe | 30 | 18 | 60.0 |
|  | Homalin | 30 | 26 | 86.7 |
| NMCP | Chaungzon | 27 | 25 | 92.6 |
|  | Kyaukse and Thazi | 30 | 28 | 93.3 |
| **Total** |  | **200** | **165** | **82.5** |

^a^Target cluster size includes the oversampling component for non-participation

Table S3. Sampling units and eligible ICMVs: Counts

| **Organization** | **Sampling unit (cluster)** | **ICMVs** | **Eligible ICMVs** | **Weighted ICMVs** |
| --- | --- | --- | --- | --- |
| IOM | 2 | 48 | 48 | 31 |
| HPA | 1 | 20 | 19 | 8 |
| SC-SR | 2 | 44 | 44 | 38 |
| NMCP | 2 | 53 | 52 | 86 |
| **Total** | **7** | **165** | **163** | **163** |

**Table S4.** Malaria case-based reporting by 163 surveyed ICMV

|  | **N (%)** |
| --- | --- |
| **Number of ICMV surveyed** | 163 |
| **Mostly used method of reporting malaria data** |  |
| PBR for all patients and MCBR for some of them | 21 (13.1) |
| MCBR for all patients and PBR for some of them | 11 (6.7) |
| Used PBR and MCBR for all patients | 128 (80.3) |

**Table S5**: Practice and perspectives of 163 ICMV survey and 38 observed ICMVs regarding stock management function of MCBR

|  | **N (%)**^a^ |
| --- | --- |
| **Number of ICMV surveyed** | 163 |
| **Do you use MCBR for malaria stock management?** |  |
| Yes | 70 (43.0) |
| No | 93 (57.1) |
| **MCBR is easier to report stock-outs^b^** |  |
| Strongly disagree | 0 |
| Disagree | 4 (6.3) |
| Neutral | 7 (10.5) |
| Agree | 33 (47.3) |
| Strongly agree | 25 (35.9) |
| **MCBR makes it easier to check stock balance ^a^** |  |
| Strongly disagree | 2 (2.4) |
| Disagree | 20 (29.0) |
| Neutral | 2 (3.0) |
| Agree | 31 (44.6) |
| Strongly agree | 15 (21.0) |
| **Number of ICMVs observed in the field** | 38 |
| **Issues with stock management** |  |
| Yes | 16 (42.1) |
| No | 22 (57.9) |
| **Stock management issues (Multiple response)** |  |
| Stock discrepancies between PBR and MCBR | 6 (37.5) |
| Errors in MCBR stock records | 8 (50.5) |
| Errors in PBR stock records | 4 (25.0) |
| Stock out | 2 (12.5) |
| Do not know how to use | 1 (6.2) |

^a^Denominators vary for each question due to the application of inverse proportional sampling weights

^b^Question asked only in those who use MCBR for stock management. In FGD, ICMVs mentioned that the module did not allow the inclusion of invalid and damaged RDTs that renders discrepancy between the stock balance in MCBR and on ground actual balance.

**Table S6:** Experience of MCBR use by 163 surveyed ICMVs

|  | **N (%)** |
| --- | --- |
| **Number of ICMV surveyed**  **MCBR application error experienced** | 163 |
| **Unable to load MCBR application** |  |
| Yes, frequently | 1 (7.0) |
| Yes, occasionally | 20 (12.5) |
| No, never | 141 (86.8) |
| **Application failure (crash) while using** |  |
| Yes, frequently | 4 (2.5) |
| Yes, occasionally | 21 (13.0) |
| No, never | 138 (84.5) |
| **Unable to enter data in MCBR application** |  |
| Yes, frequently | 1 (0.8) |
| Yes, occasionally | 8 (5.2) |
| No, never | 153 (94.0) |
| **Unable to send the entered data** |  |
| Yes, frequently | 16 (10.0) |
| Yes, occasionally | 64 (39.1) |
| No, never | 83 (50.9) |
| **Delays when using MCBR application** |  |
| Yes, frequently | 16 (10.1) |
| Yes, occasionally | 92 (57.0) |
| No, never | 53 (32.9) |

**Table S7.** Training and support needed for MCBR: Results from survey of 163 ICMVs

|  | **N (%)** |
| --- | --- |
| **Number of ICMV surveyed** | 163 |
| **Did you receive adequate training for use of MCBR?** |  |
| Yes | 148 (90.9) |
| No | 15 (9.1) |
| **What additional training would you like to receive?** |  |
| How to use MCBR application | 69 (42.5) |
| How to type Burmese font using Unicode system | 57(34.7) |
| Basic mobile phone usage | 51 (31.2) |
| Refresher training for MCBR application | 15 (9.1) |
| Stock management using MCBR application | 8 (4.9) |
| Any training on MCBR application (unspecified) | 3 (2.1) |
| Data retrieving using MCBR application | 2 (1.5) |
| Others | 3 (1.9) |

Table S8: One-off (capital) costs for development of reporting tools

| **Costs** | | **Unit cost^a^** | **Estimate lifetime in years or renewal frequency** | **Cost per year** | **% contribution** | | **Total cost** | |
| --- | --- | --- | --- | --- | --- | --- | --- | --- |
|  |  |  |  |  | **MCBR** | **PBR** | **MCBR** | **PBR** |
| MCBR Software development and DHIS2 configuration | | $ 69.24 | 3 | $ 57419.33 | 100 |  | $ 57419.33 | $ - |
| Hosting and administration of MCBR Server | | $ 12.67 | 3 | $ 10505.00 | 100 |  | $ 10505.00 | $ - |
| Printing of case register books | | $ 4.67 | 1 | $ 11610.67 |  | 100 | $ - | $ 11610.67 |
| IT Equipment | Sim-card | $ 1.00 | 2 | $ 1244.00 | 100 |  | $ 1244.00 | $ - |
|  | Mobile phone | $ 100.00 | 2 | $ 124400.00 | 100 |  | $ 124400.00 | $ - |
| PBR ToT - access database training | | $ 2400.00 | 1 | $ 2400.00 |  | 100 | $ - | $ 2400.00 |
| MCBR ToT training | | $ 15995.26 | 1 | $ 15995.26 | 100 |  | $ 15995.26 | $ - |
| Total cost for 1527 ICMVs | | | | | | | $ 209563.59 | $14010.67 |
| Total cost for 20000 ICMVs (This is the one-off cost and once invested, it will last 3 years.  Therefore, a year cost is equal to 3 years cost.) | | | | | | | $1093919.59 | $112625.94 |
| Cost per ICMV for 1 year (for 1527 ICMV, in 2018) | | | | | | | $84.23 | $5.63 |
| Cost per ICMV for 3 years (for 1527 ICMV) | | | | | | | $28.08 | $5.63 |
| Cost per ICMV for 1 year (for 20000 ICMV, in 2018) | | | | | | | $54.70 | $5.63 |
| Cost per ICMV for 3 years (for 20000 ICMV) | | | | | | | $18.23 | $5.63 |
| Number of tested and reported clients by 1527 ICMVs in 2018 | | | | | | | 48922 | 106743 |
| Cost per test reported per year from 1527 ICMVs in 2018 | | | | | | | $4.28 | $0.131 |
| Cost per test reported per 3 years^b^ from1527 ICMVs | | | | | | | $1.43 | $0.131 |
| Number of tested and reported clients nationwide in 2018^c^ | | | | | | |  | 2136943 |
| Cost per test reported per year nationwide in 2018 | | | | | | | $0.512 | $0.131 |
| Cost per test reported per 3 years^b^ nationwide | | | | | | | $0.171 | $0.131 |

^a^ 1 Unit = 1 ICMV;

^b^ Assumption is that the reported testing number will be the same over 3 years);

^c^ Data was provided by NMCP on 12 May 2020

Table S9: One-off (capital) costs for development of reporting tools over 3 years (with 1527 ICMVs)

| **Item** | **Cost (2018 US$)** |  | **Cost applies to** | **Estimate lifetime in years or renewal frequency** | **System** | **Total cost in first year for 1527 ICMVs (2018 US$)** | **Total cost over three years for 1527 ICMVs (2018 US$)** |
| --- | --- | --- | --- | --- | --- | --- | --- |
| MCBR Software development and DHIS2 configuration | 57419.33 |  | Shared resource for all ICMVs | 3 | MCBR | 57419.33 | 57419.33 |
| Hosting and administration of MCBR Server | 10505.00 |  | Shared resource for all ICMVs | 3 | MCBR | 10505.00 | 10505.00 |
| MCBR ToT training | 15995.26 |  | One-off for all ICMVs | 1 | MCBR | 15995.26 | 47985.78 |
| IT Equipment (sim card) | 1.00 |  | Each of the 1527 ICMVs | 2 | MCBR | 1244.00 | 3732.00 |
| IT Equipment (mobile phone) | 100.00 |  | Each of the 1527 ICMVs | 2 | MCBR | 124400.00 | 373200.00 |
| PBR ToT - access database training | 2400.00 |  | One-off for all ICMVs | 1 | PBR | 2400.00 | 7200.00 |
| Printing of case register books | 4.67 |  | Each of the 1527 ICMVs | 1 | PBR | 11610.67 | 34832.01 |

The total of one-off costs associated with the implementation of MCBR among 1527 ICMV in the first year was calculated to be $209563.59, or $84.23 per ICMV. The total one-off costs associated with PBR were $14010.67, or $5.63 per ICMV. The one-off costs reduced to $54.70 per ICMV assuming MCBR were expanding to 20000 ICMV over one year ($1093919.59 / 20000). However, the annual one-off cost for an ICMV remained at $5.63 (the total annual one-off cost = $ 112625.94 / 20000 ICMVs) for nationwide implementation of PBR given more training sessions and case register books are needed to cover all ICMVs. The costs associated with nationwide implementation over a 3-years period were also calculated, reflecting the expected lifetime for most of the capital items: the one-off cost for an ICMV was further reduced to $18.23 (yearly cost = 54.70/ 3 years) while the per ICMV cost for PBR remained the same at $5.63.

The one-off costs associated with reporting a malaria test conducted by an ICMV in the community were also calculated. Reporting a tested client in the first year (one-off) cost $4.28 ($209563.59 / 48922 tests) using the MCBR system and $0.13 ($14010.67 / 106743 tests) using the PBR system (Table S5). If all malaria testing was reported via the MCBR system nationwide^^[[1]](#footnote-1)^^, the per test reported one-off cost was reduced to $0.51 ($ 1093919.59 / 2136943 tests) and it further reduced to $0.17 ($ 0.51 / 3 years) over 3 years implementation while the cost for PBR channel remains the same at $ 0.13.

**Table S10:** Ongoing costs associated with MCBR and PBR

**Organization 1**

| **Description of expense** | | **Unit cost** | **No of units per year** | **Unit of measurement** | **Cost per year** | **% contribution** | | **Total cost** | | **Cost per ICMV per annum** | |
| --- | --- | --- | --- | --- | --- | --- | --- | --- | --- | --- | --- |
|  |  |  |  |  |  | **MCBR** | **PBR** | **MCBR** | **PBR** | **MCBR** | **PBR** |
| Human Resources (Management and Support Staff) | Position 1 | $ 1984 | 12 | person month | $ 23803 | 5 | 5 | $ 1190.14 | $ 1190.14 | $ 4.05 | $ 4.05 |
|  | Position 2 | $ 95 | 12 | person month | $ 1138 | 10 | 30 | $ 113.77 | $ 341.30 | $ 0.39 | $ 1.16 |
| Human Resources (Program Implementation Staff) | Position 1 | $ 79 | 12 | person month | $ 3344 | 20 | 80 | $ 668.82 | $ 2675.29 | $ 2.27 | $ 9.10 |
|  | Position 2 | $ 1408 | 12 | person month | $ 16895 | 10 | 20 | $ 1689.50 | $ 3379.01 | $ 5.75 | $ 11.49 |
|  | Position 3 (at township level) | $ 684 | 12 | person month | $ 8208 | 20 | 80 | $ 1641.59 | $ 6566.35 | $ 5.58 | $ 22.33 |
| ICMV incentive | | $ 33 | 4 | person month | $ 39200 | 20 | 20 | $ 7840.00 | $ 7840.00 | $ 26.67 | $ 26.67 |
| Field visits to villages for PBR data collection/ technical support and supervision for MCBR reporting by field staff | Per diem | $ 1.0 | 12 | per person per month | $ 3497 | 10 | 30 | $ 349.72 | $ 1049.15 | $ 1.19 | $ 3.57 |
|  | Travel | $ 0.4 | 12 | round trip | $ 1345 | 10 | 30 | $ 134.51 | $ 403.52 | $ 0.46 | $ 1.37 |
|  | Accommodation | $ 1.0 | 12 | per person per month | $ 3980 | 10 | 30 | $ 398.03 | $ 1194.09 | $ 1.35 | $ 4.06 |
| Phone credit for MCBR reporting | | $ 2 | 12 | per VHV per month | $ 7056 | 100 | - | $ 7056.00 | $ - | $ 24.00 | $ - |
| VHV's travel cost from his/her residence to a location that has mobile phone network coverage* | | $ 3 | 12 | round trip | $ 1176 | 100 | 30 | $ 1176.00 | $ 352.80 | $ 40.00 | $ 12.00 |
| Routine field monitoring visits of (YGN-Field-YGN) Program Team | Per diem | $ 0.8 | 12 | per person per month | $ 2798 | 10 | 10 | $ 279.77 | $ 279.77 | $ 0.95 | $ 0.95 |
|  | Travel | $ 0.9 | 12 | round trip | $ 3228 | 10 | 10 | $ 322.82 | $ 322.82 | $ 1.10 | $ 1.10 |
|  | Accommodation | $ 0.3 | 12 | per person per month | $ 1211 | 10 | 10 | $ 121.06 | $ 121.06 | $ 0.41 | $ 0.41 |
| VHV (initial) training for case reporting with mobile application- MCBR | | $ 25 | 1 | training | $ 7249.42 | 100 |  | $ 7249.42 | $ - | $ 24.66 | $ - |
| VHV training for case reporting with mobile application- PBR | Initial training | $ 45 | 1 | training | $ 13339.52 |  | 20 | $ - | $ 2667.90 | $ - | $ 9.07 |
|  | Refresher training | $ 96 | 1 | training | $ 28111 |  | 20 | $ - | $ 5622.11 | $ - | $ 19.12 |
| VHV meeting (at a town for data collection) | Meeting | $ 10 | 12 | meeting | $ 33642.40 | 20 | 80 | $ 6728.48 | $ 26913.92 | $ 22.89 | $ 91.54 |
| Total cost | |  |  |  |  |  |  | $ 36959.62 | $ 60919.25 | **$ 161.71** | **$ 218.01** |

**Organization 2**

| **Description of expense** | | **Unit cost** | **No of units per year** | **Unit of measurement** | **Cost per year** | **% contribution** | | **Total cost** | | **Cost per ICMV per annum** | |
| --- | --- | --- | --- | --- | --- | --- | --- | --- | --- | --- | --- |
|  |  |  |  |  |  | **MCBR** | **PBR** | **MCBR** | **PBR** | **MCBR** | **PBR** |
| Human Resources (Management and Support Staff) | Position 1 | $ 1335 | 12 | person month | $ 16016 | 5 | 5 | $ 800.79 | $ 800.79 | $ 11.12 | $ 11.12 |
|  | Position 2 | $ 336 | 12 | person month | $ 4026 | 10 | 30 | $ 402.63 | $ 1207.89 | $ 5.59 | $ 16.78 |
| Human Resources (Program Implementation Staff) | Position 1 | $ 559 | 12 | person month | $ 6704 | 20 | 80 | $ 1340.80 | $ 5363.20 | $ 18.62 | $ 74.49 |
|  | Position 2 | $ 2531 | 12 | person month | $ 30370 | 10 | 20 | $ 3037.02 | $ 6074.04 | $ 42.18 | $ 84.36 |
|  | Position 3 (at township level) | 0 | 12 | person month | $ - |  |  | $ - | $ - | $ - | $ - |
| ICMV incentive | | $ 33 | 4 | person month | $ 9600 | 20 | 20 | $ 1920.00 | $ 1920.00 | $ 26.67 | $ 26.67 |
| Field visits to villages for PBR data collection/ technical support and supervision for MCBR reporting by field staff | Per diem | $ 11 | 12 | per person per month | $ 9752.82 | 10 | 30 | $ 975.28 | $ 2925.85 | $ 13.55 | $ 40.64 |
|  | Travel | $ 3 | 12 | round trip | $ 2438.21 | 10 | 30 | $ 243.82 | $ 731.46 | $ 3.39 | $ 10.16 |
|  | Accommodation | $ 1 | 12 | per person per month | $ 487.64 | 10 | 30 | $ 48.76 | $ 146.29 | $ 0.68 | $ 2.03 |
| Phone credit for RDT provider case reporting | | $ 13 | 12 | per VHV per month | $ 11494.40 | 100 | - | $ 11494.40 | $ - | $ 159.64 | $ - |
| VHV's travel cost from his/her residence to a location that has mobile phone network coverage* | | $ 3 | 12 | round trip | $ 288 | 100 | - | $ 288.00 | $ - | $ 40.00 | $ - |
| Routine Field Monitoring Visits of (YGN-Field-YGN) Program Team | Per diem | $ 0.3 | 6 | per person per month | $ 135 | 10 | 10 | $ 13.55 | $ 13.55 | $ 0.19 | $ 0.19 |
|  | Travel | $ 1.1 | 6 | round trip | $ 484 | 10 | 10 | $ 48.38 | $ 48.38 | $ 0.67 | $ 0.67 |
|  | Accommodation | $ 0.4 | 6 | per person per month | $ 174 | 10 | 10 | $ 17.42 | $ 17.42 | $ 0.24 | $ 0.24 |
| VHV training for case reporting with mobile application- MCBR | Initial training | $ 37 | 1 | training | $ 2636.40 | 100 |  | $ 2636.40 | $ - | $ 36.62 | $ - |
|  | Refresher training | $ - | 1 | training | $ - | 100 |  | $ - | $ - |  |  |
| VHV (initial) training for PBR reporting | | $ 89 | 1 | training | $ 6377.64 |  | 20 | $ - | $ 1275.53 | $ - | $ 17.72 |
| Total cost | |  |  |  |  |  |  | $ 23267.25 | $ 20524.4 | **$ 359.16** | **$ 285.06** |

**Organization 3**

| **Description of expense** | | **Unit cost** | **No of units per year** | **Unit of measurement** | **Cost per year** | **% contribution** | | **Total cost** | | **Cost per ICMV per annum** | |
| --- | --- | --- | --- | --- | --- | --- | --- | --- | --- | --- | --- |
|  |  |  |  |  |  | **MCBR** | **PBR** | **MCBR** | **PBR** | **MCBR** | **PBR** |
| Human Resources (Management and Support Staff) | Position 1 | $ 1870 | 12 | person month | $ 22444 | 5 | 5 | $ 1122.21 | $ 1122.21 | $ 3.12 | $ 3.12 |
|  | Position 2 | $ 215 | 12 | person month | $ 2584 | 10 | 30 | $ 258.37 | $ 775.11 | $ 0.72 | $ 2.15 |
| Human Resources (Program Implementation Staff) | Position 1 | $ 2798 | 12 | person month | $ 33572 | 20 | 80 | $ 6714.40 | $ 26857.60 | $ 18.65 | $ 74.60 |
|  | Position 2 | $ 4119 | 12 | person month | $ 49428 | 10 | 20 | $ 4942.80 | $ 9885.60 | $ 13.73 | $ 27.46 |
|  | Position 3 (at township level) | 0 | 12 | person month | $ - | 20 | 80 | $ - | $ - | $ - | $ - |
| ICMV incentive | | $ 33 | 4 | person month | $ 48000.00 | 20 | 20 | $ 9600.00 | $ 9600.00 | $ 26.67 | $ 26.67 |
| Field visits to villages for PBR data collection/ technical support and supervision for MCBR reporting by field staff | Per diem | $ 0.6 | 12 | per person per month | $ 2431 | 10 | 30 | $ 243.11 | $ 729.32 | $ 0.68 | $ 2.03 |
|  | Travel | $ 0.4 | 12 | round trip | $ 1804 | 10 | 30 | $ 180.38 | $ 541.13 | $ 0.50 | $ 1.50 |
|  | Accommodation | $ 1.2 | 12 | per person per month | $ 5394 | 10 | 30 | $ 539.45 | $ 1618.34 | $ 1.50 | $ 4.50 |
| Phone credit for RDT provider case reporting | | $ 2 | 12 | per VHV per month | $ 8640 | 100 | - | $ 8640.00 | $ - | $ 24.00 | $ - |
| VHV's travel cost from his/her residence to a location that has mobile phone network coverage* | | $ 6.7 | 12 | round trip | $ 2880 | 100 | - | $ 2880.00 | $ - | $ 80.00 | $ - |
| Routine Field Monitoring Visit (YGN-Field-YGN) Program Team | Per diem | $ 0.1 | 12 | per person per month | $ 539 | 10 | 10 | $ 53.94 | $ 53.94 | $ 0.15 | $ 0.15 |
|  | Travel | $ 0.1 | 12 | round trip | $ 601 | 10 | 10 | $ 60.13 | $ 60.13 | $ 0.17 | $ 0.17 |
|  | Accommodation | $ 0.3 | 12 | per person per month | $ 1347 | 10 | 10 | $ 134.68 | $ 134.68 | $ 0.37 | $ 0.37 |
| VHV initial training for case reporting with mobile application- MCBR | | $ 37 | 1 | training | $ 13258.54 | 100 |  | $ 13258.54 | $ - | $ 36.83 | $ - |
| VHV refresher training for case reporting - PBR | | $ 40 | 1 | meeting | $ 14269.55 |  | 20 | $ - | $ 2853.91 | $ - | $ 7.93 |
| VHV meeting (at a town and data collection) | | $ 18 | 4 | meeting | $ 26032.31 | 20 | 80 | $ 5206.46 | $ 20825.84 | $ 14.46 | $ 57.85 |
| Total cost | |  |  |  |  |  |  | $ 53834.47 | $ 75057.82 | **$ 221.54** | **$ 208.49** |

Note - Total number of ICMVs in SC SR is 360 * Assumption – only 10 % (36 ICMVs) needs travel support

**Organization 4**

| **Description of expense** | **Unit cost** | **Units per year** | **Unit of measurement** | **Cost per year** | **% contribution** | | **Total cost** | | **Cost per ICMV per annum** | |
| --- | --- | --- | --- | --- | --- | --- | --- | --- | --- | --- |
|  |  |  |  |  | **MCBR** | **PBR** | **MCBR** | **PBR** | **MCBR** | **PBR** |
| ICMV incentive | $ 33.33 | 4 | person month | $ 106800 | 20 | 20 | $ 21360 | $ 21360 | $ 26.67 | $ 26.67 |
| Phone credit for RDT provider case reporting |  |  | per VHV per month | 0 | 100 |  | $ 9845.97 | $ - | $ 12.29 | $ - |
| VHV (initial) training for case reporting with mobile application - MCBR |  |  | training | 0 | 100 |  | $ 70409.82 | $ - | $ 87.90 | $ - |
| Total cost |  |  |  |  |  |  | $ 101615.79 | $ 21360.00 | **$ 126.86** | **$ 26.67** |

Ongoing costs associated with MCBR and PBR

The ongoing costs for each IP (NMCP, IOM, HPA and SC SR) were calculated separately given the cost of implementing MCBR and PBR were significantly different among IPs. The ongoing costs included human resources costs (staff salaries and ICMV incentives), travel associated costs for supervision and data collection, training costs, and other direct costs for ICMVs such as phone credit for ICMV’s mobile phones and travel cost from ICMV’s residence to a location that has mobile phone network coverage.

Overall, human resources cost contributed 32.72% in MCBR while it was 68.81% in PBR given IP staff had to spend more time in PBR compared to MCBR. Similarly, staff travel cost contribution in MCBR was only 3.1% while in PBR was 7.16% as staff from HPA and SC SR needed to visit villages for PBR data collection. On the other hand, 26% of the total cost the MCBR system was associated with ICMV phone credit and cost of travel from ICMV’s residence to a location that has mobile phone network coverage, while these costs in PBR system was almost nil (0.14%).

1. According to NMCP, a total of 2,136,943 tests (NMCP trained ICMVs: 458,804 tests + IP trained ICMVs: 1,678,139 tests) were reported. [↑](#footnote-ref-1)
