# Supplementary material for: A mobile phone application for malaria case-based reporting to advance malaria surveillance in Myanmar: a mixed methods evaluation
Source: Malar J. 2021 Mar 26;20:167. doi: 10.1186/s12936-021-03701-6 (PMC7995396; doi:10.1186/s12936-021-03701-6)
Supplement: Supplementary file 5 — Additional file 5. Completed STROBE checklist. [file 12936_2021_3701_MOESM5_ESM.docx]

STROBE Statement—checklist of items that should be included in reports of observational studies

|  | Item No. | Recommendation | Page  No. | Relevant text from manuscript | GNH Remarks |
| --- | --- | --- | --- | --- | --- |
| **Title and abstract** | 1 | (*a*) Indicate the study’s design with a commonly used term in the title or the abstract | 1 | a Mixed Methods Evaluation |  |
|  |  | (*b*) Provide in the abstract an informative and balanced summary of what was done and what was found | 2-3 | Abstract  (Study setting, Population, Outcome measures (Measurements), Limitations, Conclusion are not described.) |  |
| Introduction | | | |  |  |
| Background/rationale | 2 | Explain the scientific background and rationale for the investigation being reported | 4-5 | Background |  |
| Objectives | 3 | State specific objectives, including any prespecified hypotheses | 5 | Introduction (ln. 103– 108) | (We report a…… system.) |
| Methods | | | |  |  |
| Study design | 4 | Present key elements of study design early in the paper | 6-8 | Study design, data collection and analyses (ln. 132 – 166), Additional files 6 and 7 | The mixed methods ….….. end of 2019 (2,488). |
| Setting | 5 | Describe the setting, locations, and relevant dates, including periods of recruitment, exposure, follow-up, and data collection | 6 | MCBR application and study setting, Figure 1 and Supplementary table 1 |  |
| Participants | 6 | 1. *Cohort study*—Give the eligibility criteria, and the sources and methods of selection of participants. Describe methods of follow-up   *Case-control study*—Give the eligibility criteria, and the sources and methods of case ascertainment and control selection. Give the rationale for the choice of cases and controls  *Cross-sectional study*—Give the eligibility criteria, and the sources and methods of selection of participants | 7-9 | **Main manuscript**  Study design, data collection and analyses (ln. 131 – 200)  **Additional file 7**   - ICMV questionnaires > Sampling method (ln. 61 – 63) - Focus group discussions, key informant interviews and in-depth interviews > Participant recruitment (ln. 107 – 109) - Field observations of ICMVs (ln. 150 – 161) | (To achieve …… managing malaria reporting data.)  (A list of …. both the MCBR application and PBR.)  (FGDs were conducted ……team members.)  (ICMVs were selected ……. before the observation.) |
|  |  | (*b*) *Cohort study*—For matched studies, give matching criteria and number of exposed and unexposed  *Case-control study*—For matched studies, give matching criteria and the number of controls per case |  |  |  |
| Variables | 7 | Clearly define all outcomes, exposures, predictors, potential confounders, and effect modifiers. Give diagnostic criteria, if applicable | 2 | **Manuscript**  Methods (ln. 32 – 35) | (We undertook ……system in Myanmar.)  The outcome variables are not clearly described. |
| Data sources/ measurement | 8* | For each variable of interest, give sources of data and details of methods of assessment (measurement). Describe comparability of assessment methods if there is more than one group | 6 | **Manuscript**  Study design, data collection and analyses (ln. 132 – 200)  **Additional file 6**  Secondary data analysis of malaria test report data  (ln. 25 – 40) |  |
| Bias | 9 | Describe any efforts to address potential sources of bias |  | The bias is not described. |  |
| Study size | 10 | Explain how the study size was arrived at | 7 | **Manuscript**  Study design, data collection and analyses (ln. 161 – 166)  **Additional file 6**  Sampling method (ln. 49 – 59) |  |

Continued on next page

| Quantitative variables | 11 | Explain how quantitative variables were handled in the analyses. If applicable, describe which groupings were chosen and why |  | **Additional file 6**   - Secondary data analysis of malaria test report data - Analysis of questionnaire responses - Cost analysis |  |
| --- | --- | --- | --- | --- | --- |
| Statistical methods | 12 | (*a*) Describe all statistical methods, including those used to control for confounding |  | **Additional file 6**   - Secondary data analysis of malaria test report data - Analysis of questionnaire responses - Cost analysis |  |
|  |  | (*b*) Describe any methods used to examine subgroups and interactions |  | There is no description for examination for subgroups. |  |
|  |  | (*c*) Explain how missing data were addressed |  | There is no description for how missing data were addressed. |  |
|  |  | (*d*) *Cohort study*—If applicable, explain how loss to follow-up was addressed  *Case-control study*—If applicable, explain how matching of cases and controls was addressed  *Cross-sectional study*—If applicable, describe analytical methods taking account of sampling strategy |  |  |  |
|  |  | (*e*) Describe any sensitivity analyses |  | There is no description for sensitivity analysis. |  |
| Results | | | | |  |
| Participants | 13* | (a) Report numbers of individuals at each stage of study—eg numbers potentially eligible, examined for eligibility, confirmed eligible, included in the study, completing follow-up, and analysed | 9 | **Manuscript**   - MCBR and PBR usability and adherence to standard guidelines (ln. 229)   **Additional file 6**   - ICMV questionnaires (ln. 61 – 66) and Additional table M1 - Focus group discussions, key informant Interviews and in-depth Interviews (ln. 107 – 109) - Field observations of ICMVs (ln. 147 – 152) | Sampling method: (A list of ….. in their villages.)  Participant recruitment: (FGD were …… team members.)  (Researchers conducted …… (ABER) in 2018.) |
|  |  | (b) Give reasons for non-participation at each stage |  | There is no description of reasons for non-participation. |  |
|  |  | (c) Consider use of a flow diagram |  | There is no diagram for “Participants”. |  |
| Descriptive data | 14* | (a) Give characteristics of study participants (eg demographic, clinical, social) and information on exposures and potential confounders |  | There is no description on characteristics of study participants. |  |
|  |  | (b) Indicate number of participants with missing data for each variable of interest |  | There is no description on number of participant or records with missing. |  |
|  |  | (c) *Cohort study*—Summarise follow-up time (eg, average and total amount) |  |  |  |
| Outcome data | 15* | *Cohort study*—Report numbers of outcome events or summary measures over time |  |  |  |
|  |  | *Case-control study—*Report numbers in each exposure category, or summary measures of exposure |  |  |  |
|  |  | *Cross-sectional study—*Report numbers of outcome events or summary measures | 7 – 9  10  11  11 – 12  13 – 16  16 | - Data quality of MCBR and PBR systems (ln. 204 – 211) and Table 1 - MCBR and PBR usability and adherence to standard guidelines (ln. 229), Supplementary table 2 and Figure 2 - Timely reporting (ln. 253 – 266), Table 2 and Table 3 - Facilitators and barriers MCBR usage (ln. 248 – 251) and Supplementary table S3, (ln. 260 – 262) and Supplementary table S4 - Data access and stakeholder utilisation - Cost analysis (ln. 357 – 363), Table 4, Supplementary table S5 and S6 |  |
| Main results | 16 | (*a*) Give unadjusted estimates and, if applicable, confounder-adjusted estimates and their precision (eg, 95% confidence interval). Make clear which confounders were adjusted for and why they were included | 9-10 | Data quality of MCBR and PBR systems (ln. 204 – 226) and Table 1 |  |
|  |  | (*b*) Report category boundaries when continuous variables were categorized |  |  |  |
|  |  | (*c*) If relevant, consider translating estimates of relative risk into absolute risk for a meaningful time period |  |  |  |

Continued on next page

| Other analyses | 17 | Report other analyses done—eg analyses of subgroups and interactions, and sensitivity analyses |  | Supplementary tables, Additional file 3 |  |
| --- | --- | --- | --- | --- | --- |
| Discussion | | | | |  |
| Key results | 18 | Summarise key results with reference to study objectives | 18 - 19 | Discussion and conclusion (ln. 424 – 435 |  |
| Limitations | 19 | Discuss limitations of the study, taking into account sources of potential bias or imprecision. Discuss both direction and magnitude of any potential bias | 21 | Discussion and conclusion (ln. 492 – 513) |  |
| Interpretation | 20 | Give a cautious overall interpretation of results considering objectives, limitations, multiplicity of analyses, results from similar studies, and other relevant evidence | 22 | Discussion and conclusion (ln. 515 – 432) |  |
| Generalisability | 21 | Discuss the generalisability (external validity) of the study results | 21 | Discussion and conclusion (ln. 483 – 513) |  |
| Other information | |  | | |  |
| Funding | 22 | Give the source of funding and the role of the funders for the present study and, if applicable, for the original study on which the present article is based | 25 | Funding |  |

*Give information separately for cases and controls in case-control studies and, if applicable, for exposed and unexposed groups in cohort and cross-sectional studies.

**Note:** An Explanation and Elaboration article discusses each checklist item and gives methodological background and published examples of transparent reporting. The STROBE checklist is best used in conjunction with this article (freely available on the Web sites of PLoS Medicine at http://www.plosmedicine.org/, Annals of Internal Medicine at http://www.annals.org/, and Epidemiology at http://www.epidem.com/). Information on the STROBE Initiative is available at www.strobe-statement.org.
