# Supplementary material for: A mobile phone application for malaria case-based reporting to advance malaria surveillance in Myanmar: a mixed methods evaluation
Source: Malar J. 2021 Mar 26;20:167. doi: 10.1186/s12936-021-03701-6 (PMC7995396; doi:10.1186/s12936-021-03701-6)
Supplement: Supplementary file 6 — Additional file 6. Additional methods. [file 12936_2021_3701_MOESM6_ESM.docx]

**ICMV Questionnaire**

| **General background Information** | | |
| --- | --- | --- |
| 0.1 | Name of interviewer: |  |
| 0.2 | Date (DD/MM/YYYY): |  |
| 0.3 | Start time: |  |
| 0.4 | End time: |  |
| 0.5 | Township name: |  |
| 0.6 | Name of organization | 1. IOM 2. HPA 3. SC-SR 4. NMCP |
| **Background information of ICMV** | | |
| 0.7 | Completed age (in years): |  |
| 0.8 | Gender | 1. Male 2. Female |
| 0.9 | Highest education level | 1. No formal education 2. Primary school level 3. Secondary school level 4. High school level 5. Degree holder 6. Others (specify) |
| 0.10 | How long have you worked as a Village Health Volunteer? | ­­­______years ______ months |
| 0.11 | Type of mobile phone using for MCBR | 1. Samsung J series 2. Samsung A series 3. Samsung S series 4. Xiaomi 5. Other (specify) |
| 0.12 | Is the mobile phone using for MCBR provided by respective organization? | 1. Yes 2. No |

| **Section 1: Education level and e-literacy of village health volunteer.** | | |
| --- | --- | --- |
| 1.1 | How long have you been using a mobile phone? | ______years ______ months |
| 1.2 | Purpose for using mobile phone **(Select all that apply)** | 1. For reporting malaria cases 2. For communicating with friends/relatives via phone calls/messaging 3. For social networking 4. For banking and shopping 5. For seeking general knowledge 6. Other (Specify) |
| 1.3 | Do you know how to use mobile data on phone? | 1. Yes 2. No |
| 1.4 | Do you know how to get access to mobile applications that you want to use? | 1. Yes 2. No |
| 1.5 | Do you know basic messaging on your phone? | 1. Yes 2. No |
| 1.6 | Do you know file sharing on your phone? | 1. Yes 2. No |
| **Section 2: Village/worksite characteristics** | | |
| 2.1 | Approximately how many households are in your village/worksite(s)? | ___ households |
| 2.2 | What is the approximate total population size of your village/worksite(s)? | ___ persons |
| 2.3 | Is there mobile phone signal in your village/worksite (s)? (Any SIM card) | 1. Yes 2. No |
| 2.3.1 | If yes, is the mobile phone signal good at your village/worksite(s)? | 1. Yes 2. No |
| 2.4 | Is there Internet access at your village/worksite(s)? | 1. Yes 2. No |
| 2.4.1 | If yes, is the internet access good at your village/worksite(s)? | 1. Yes 2. No |
| **Section 3: Malaria testing** | | |
| 3.1 | On average, how many malaria rapid diagnostic tests do you perform in one month? | ­­___ tests |
| 3.2 | On average, how many days per month do you perform malaria rapid diagnostic tests? | ___ days |
| 3.3 | In general, how are you notified about possible cases of malaria in your village/worksite(s)? **(Select all that apply)** | 1. Phone call from patient 2. Phone call/referral from patient’s family member or friend 3. Patient’s visit to volunteer 4. House visit by volunteer 5. Mass testing 6. Other (Specify): |
| **Section 4: Malaria Case Based Reporting** | | |
| 4.1 | How do you currently record and report malaria cases in your community? | 1. PBR only **(go to 4.2)** 2. MCBR only **(go to 4.1.1)** 3. Both MCBR and PBR **(go to 4.1.2)** |
| 4.1.1 | If you are currently using MCBR only, did you previously use paper-based reporting? | 1. Yes 2. No |
| 4.1.2 | If you are currently using both MCBR and PBR, which method do you use most? | 1. PBR for all patients and occasionally MCBR 2. MCBR for all patients and occasionally PBR 3. Equally MCBR and PBR 4. Other (Specify) |
| 4.2 | How frequently do you submit PBR to SR? | 1. Daily 2. Weekly 3. Fortnightly 4. Monthly 5. Other (Specify) |
| 4.3 | How do you submit PBR to SRs? **(Select all that apply)** | 1. Collected by supervisor 2. ICMV go and submit to supervisor by him/herself 3. ICMV submit to supervisor via someone 4. Submit at monthly meetings 5. Submit at quarterly meetings 6. Other (specify) |
| 4.3.1 | Are there any out-of-pocket expenses associated with this? | 1. Yes 2. No **(Go to 4.4)** |
| 4.3.2 | If yes, please mention cost categories. **(Select all that apply)** | 1. Cost for filling out PBR 2. Courier charges for sending PBR 3. Other (specify) |
| 4.3.3 | If yes, how much do you have to spend each month on average? | ________ kyats |
| 4.4 | How frequently do you submit MCBR to SR? | 1. After each testing 2. Weekly 3. Fortnightly 4. Monthly 5. Compile and submit after more than one month 6. Other (Specify) |
| 4.5 | How do you submit MCBR to SRs? | 1. Submit from own village using mobile phone 2. Need to go to other places to get internet access 3. Other (specify) |
| 4.5.1 | Are there any out-of-pocket expenses associated with this? | 1. Yes 2. No **(Go to 5.1)** |
| 4.5.2 | If yes, please mention cost categories. **(Select all that apply)** | 1. Maintenance cost for mobile phone 2. Mobile top-up charges 3. Travel cost to go to other places to get internet access 4. Other (specify) |
| 4.5.3 | If yes, how much do you have to spend each month on average? | ________ kyats |
| **Section 5: Ease of use of MCBR and PBR** | | |
| 5.1 | How easy is it to use is the MCBR? | 1. Very difficult 2. Difficult 3. Neither difficult nor easy 4. Easy 5. Very easy |
| 5.2 | How easy is it to use is the PBR? | 1. Very difficult 2. Difficult 3. Neither difficult nor easy 4. Easy 5. Very easy |
| 5.3 | Which one is easier for you to use: MCBR or PBR? | 1. MCBR is easier 2. PBR is easier 3. They are similar |
| **Section 6: Benefits and difficulties of use in the field** | | |
| 6.1 | What are the benefits to using MCBR? **(Select all that apply)** | 1. Timely reporting 2. Can report at any time because it can be done with my mobile 3. Doesn’t need other tools as it only needs a mobile 4. Can communicate with my supervisors easily using the mobile phone provided for MCBR 5. Other (specify) |
| 6.2 | What are the benefits to using PBR? **(Select all that apply)** | 1. It is familiar and doesn’t need to learn new things 2. No technological difficulties 3. Easy to see previous records 4. Easy to make corrections if needed 5. Other (specify) |
| 6.3 | What are the main difficulties in using MCBR in your daily work? **(Select all that apply)** | 1. No difficulties at all 2. Small screen 3. Poor performance of mobile phone 4. Inaccessibility to internet 5. Poor internet connection 6. Myanmar typing 7. Not familiar to use mobile device 8. Not having enough time to do ICMV work 9. Need to charge mobile frequently as the MCBR app consumes power 10. Other (specify) |
| 6.4 | What are the main difficulties you face using PBR during your daily work? **(Select all that apply)** | 1. No difficulties at all 2. Small spaces in the register to fill in 3. Difficult to make corrections if there are mistakes in filling the PBR 4. Carbon copies are not clear 5. Village is far from reporting office 6. Losing/damaging paper records 7. Bulky/not convenient to carry 8. Not having enough time to do ICMV work 9. Other (specify) |
| 6.5 | If you do not have internet in your village/worksite(s) how far do you need to travel to use the Internet? **(No need to answer if you have internet in your village)** | _____ (miles) |
| 6.6 | How much time do you need to spend for that travel? | _____ (minutes) |
| 6.7 | Do you have adequate access to power to enable use of MCBR? | 1. Yes 2. No |
| 6.8 | Have you encountered issues with phone credit to enable consistent use of MCBR? | 1. Yes 2. No |
| 6.9 | Have you had any out-of-pocket costs associated with your use of MCBR? | 1. Yes 2. No |
| **Section 7: Functionality of MCBR** | | |
| 7.1 | Do you experience MCBR app cannot be loaded? | 1. Yes, frequently, 2. Yes, occasionally 3. No, never |
| 7.2 | Do you experience an application failure (crash) when using the MCBR? | 1. Yes, frequently, 2. Yes, occasionally 3. No, never |
| 7.3 | Do you experience not able to enter data in MCBR app? | 1. Yes, frequently, 2. Yes, occasionally 3. No, never |
| 7.4 | Do you experience data entered cannot be sent? | 1. Yes, frequently, 2. Yes, occasionally 3. No, never |
| 7.5 | Do you experience any other technical difficulties in using MCBR? | 1. Yes 2. No **(Go to 7.6)** |
| 7.5.1 | If yes, please mention. |  |
| 7.6 | Do you experience delays when using the MCBR? | 1. Yes, frequently, 2. Yes, occasionally 3. No, never **(Go to 7.7)** |
| 7.6.1 | If yes, please explain. |  |
| 7.7 | How would you rate the overall functionality of MCBR? | 1. Very poor 2. Poor 3. Acceptable 4. Good 5. Very good |
| 7.8 | MCBR app does not have a problem in uploading completed data to server (cloud). | 1. Strongly disagree 2. Disagree 3. Neutral 4. Agree 5. Strongly agree |
| 7.9 | I am feeling insecure and worried that mobile phone will be lost, stolen, damaged as I have to carry it with me. | 1. Strongly disagree 2. Disagree 3. Neutral 4. Agree 5. Strongly agree |
| **Section 8: Utility of PBR and MCBR in context of malaria case management procedures** | | |
| 8.1 | Compared to PBR, the MCBR makes it easier for me to record malaria cases. | 1. Strongly disagree 2. Disagree 3. Neutral 4. Agree 5. Strongly agree |
| 8.2 | Compared to PBR, the MCBR makes it easier to report positive cases of malaria in 24 hours. | 1. Strongly disagree 2. Disagree 3. Neutral 4. Agree 5. Strongly agree |
| 8.3 | Compared to PBR, the MCBR makes it easier to make referrals of severe malaria cases. | 1. Strongly disagree 2. Disagree 3. Neutral 4. Agree 5. Strongly agree |
| 8.4 | Compared to PBR, the MCBR allows me to test more community members for malaria. | 1. Strongly disagree 2. Disagree 3. Neutral 4. Agree 5. Strongly agree |
| **Section 9: Utility of PBR and MCBR in managing stocks** | | |
| 9.1 | Do you use MCBR for stock management? | 1. Yes 2. No **(Go to 10.1)** |
| 9.2 | Compared to PBR, the MCBR makes it easier to report stock-outs. | 1. Strongly disagree 2. Disagree 3. Neutral 4. Agree 5. Strongly agree |
| 9.3 | Compared to PBR, the MCBR makes it easier to check stock balance. | 1. Strongly disagree 2. Disagree 3. Neutral 4. Agree 5. Strongly agree |
| **Section 10: Training and support for MCBR** | | |
| 10.1 | Did you receive adequate training for use of MCBR? | 1. Yes 2. No |
| 10.2 | What additional training would you like to receive? | 1. I don’t need any more training 2. Basic mobile phone usage 3. Unicode 4. How to use MCBR apps 5. Other (specify) |
| **Section 11: Perceived value of the MCBR** | | |
| 11.1 | Using MCBR app helps collect all information needed for reporting a malaria patient. | 1. Strongly disagree 2. Disagree 3. Neutral 4. Agree 5. Strongly agree |
| 11.2 | MCBR apps improves my job performance. | 1. Strongly disagree 2. Disagree 3. Neutral 4. Agree 5. Strongly agree |
| 11.3 | MCBR app helps me to transfer/share data with my supervisors/SR quicker than paper-based collection. | 1. Strongly disagree 2. Disagree 3. Neutral 4. Agree 5. Strongly agree |
| 11.4 | Given the choice, I prefer to use MCBR over PBR. | 1. Strongly disagree 2. Disagree 3. Neutral 4. Agree 5. Strongly agree |
| 11.5 | I am likely to continue using the MCBR in my community. | 1. Strongly disagree 2. Disagree 3. Neutral 4. Agree 5. Strongly agree |

**ငှက်ဖျားရောဂါဆိုင်ရာ သတင်းအချက်အလက်များပေးပို့ရာတွင် မိုဘိုင်းအပလီကေရှင်း (MCBR) သုံးခြင်းနှင့် ကာဗွန်လက်စ် ငှက်ဖျားလချုပ်များသုံးခြင်း (PBR) တို့၏ ထိရောက်မှုကို နှိုင်းယှဉ်လေ့လာခြင်း**

လူထုအခြေပြု ဘက်စုံစေတနာ့ဝန်ထမ်း (ငှက်ဖျား)လုပ်သား (**ICMV)**များအားစစ်တမ်းကောက်ယူခြင်း

| အခြေခံအချက်အလက်များ | | | |  |
| --- | --- | --- | --- | --- |
| 0.1 | တွေ့ဆုံမေးမြန်းသူ အမည် | |  | |
| 0.2 | ရက်စွဲ (ရက်/လ/နှစ်) | |  | |
| 0.3 | စတင်ချိန် | |  | |
| 0.4 | ပြီးဆုံးချိန် | |  | |
| 0.5 | မြို့နယ် | |  | |
| 0.6 | အဖွဲ့အစည်းအမည် | | 1. IOM 2. HPA 3. SC-SR 4. NMCP | |
| **လူထုအခြေပြု ဘက်စုံစေတနာ့ဝန်ထမ်း (ငှက်ဖျား)လုပ်သား၏ အခြေခံအချက်အလက်များ** | | | | |
| 0.7 | | ပြည့်ပြီးအသက် (နှစ်) |  | |
| 0.8 | | လိင် | 1. ကျား 2. မ | |
| 0.9 | | အမြင့်ဆုံးပညာအရည်အချင်း | 1. အစိုးရကျောင်းမတက်ဖူးပါ 2. မူလတန်းအောင် 3. အလယ်တန်းအောင် 4. အထက်တန်းအောင် 5. ဘွဲ့ရ 6. အခြား (ဖော်ပြပါ)   ................................................... | |
| 0.10 | | ငှက်ဖျားစေတနာ့ဝန်ထမ်းလုပ်နေတာ ဘယ်လောက်ကြာပြီလဲ? **(မည်သည့်အဖွဲ့အစည်းတွင်မဆို)** | ...............နှစ် ...............လ | |
| 0.11 | | MCBR အတွက်အသုံးပြုနေသော မိုဘိုင်းဖုန်း အမျိုးအစား | 1. Samsung J 2. Samsung A 3. Samsung S 4. Xiaomi 5. အခြား (ဖော်ပြပါ)   ................................................... | |
| 0.12 | | MCBR အတွက်အသုံးပြုနေသော မိုဘိုင်းဖုန်းမှာ အဖွဲ့အစည်းမှထောက်ပံ့ထားသော မိုဘိုင်းဖုန်း ဟုတ်ပါသလား? | 1. ဟုတ်ပါသည်။ 2. မဟုတ်ပါ။ | |

| **အပိုင်း (၁) လူထုအခြေပြု ဘက်စုံစေတနာ့ဝန်ထမ်း (ငှက်ဖျား)လုပ်သား၏ e-literacy** | | |
| --- | --- | --- |
| 1.1 | မိုဘိုင်းဖုန်းစတင်အသုံးပြုတာ ဘယ်လောက်ကြာပြီလဲ? **(ကိုယ်ပိုင်ဖုန်းအပါအဝင်)** | ...............နှစ် ...............လ |
| 1.2 | မိုဘိုင်းဖုန်း (ကိုယ်ပိုင်ဖုန်းအပါအဝင်) အသုံးပြု ရတဲ့ရည်ရွယ်ချက်ကို ဖော်ပြပါ။  **(တစ်ခုထက်ပို၍ ဖော်ပြနိုင်သည်။)** | 1. **ငှက်ဖျားလူနာသတင်းပေးပို့ရန်** 2. **ဆွေမျိုးများ/သူငယ်ချင်းများကို ဖုန်းခေါ်ဆိုရန်/စာတိုပို့ရန်** 3. **လူမှုကွန်ယက်အသုံးပြုရန်** 4. **ဘဏ်အသုံးပြုရန်နှင့် ဈေးဝယ်ရန်** 5. **အထွေထွေဗဟုသုတရှာမှီးရန်** 6. **အခြား (ဖော်ပြပါ)**   ................................................... |
| 1.3 | မိုဘိုင်းဖုန်းမှာ မိုဘိုင်းဒေတာ အဖွင့်အပိတ် ဘယ်လိုလုပ်ရသလဲ သိပါသလား? | 1. သိပါသည်။ 2. မသိပါ။ |
| 1.4 | သင်အသုံးပြုလိုတဲ့ မိုဘိုင်းအပလီကေးရှင်းကို ဘယ်လိုဝင်ရလဲဆိုတာ သိပါသလား? | 1. သိပါသည်။ 2. မသိပါ။ |
| 1.5 | သင့်မိုဘိုင်းဖုန်းကိုသုံးပြီး စာတိုပို့တာကို ဘယ်လိုလုပ်ရလဲဆိုတာ သိပါသလား? | 1. သိပါသည်။ 2. မသိပါ။ |
| 1.6 | သင့်မိုဘိုင်းဖုန်းကိုသုံးပြီး ဖိုင်ဝေမျှတာကို ဘယ်လိုလုပ်ရလဲဆိုတာ သိပါသလား?  (ဥပမာ-Zapya အသုံးပြုပြီး သီချင်း၊ ဗီဒီယိုဖိုင်တွေ ဝေမျှတာမျိုး) | 1. သိပါသည်။ 2. မသိပါ။ |
| **အပိုင်း (၂) တာဝန်ယူထားသောကျေးရွာ/လုပ်ငန်းခွင်နှင့်ပတ်သက်သောအချက်အလက်များ** | | |
| 2.1 | သင်တာဝန်ယူထားသော ကျေးရွာ/ လုပ်ငန်းခွင်(များ)တွင် စုစုပေါင်းအိမ်ခြေ ဘယ်လောက်ရှိသလဲ? | ...............အိမ် |
| 2.2 | သင်တာဝန်ယူထားသော ကျေးရွာ/ လုပ်ငန်းခွင်(များ)တွင် စုစုပေါင်းလူဦးရေ ဘယ်လောက်ရှိသလဲ? | ...............ဦး |
| 2.3 | သင်တာဝန်ယူထားသော ကျေးရွာ/ လုပ်ငန်းခွင်(များ)တွင်မိုဘိုင်းဖုန်း လိုင်းမိလား? **(မည်သည့် Sim card နှင့်မဆို)** | 1. မိပါသည်။ 2. မမိပါ။ **(2.4 သို့သွားပါ)** |
| 2.3.1 | လိုင်းမိတယ်ဆိုရင် လိုင်းကောင်းလား? | 1. ကောင်းပါသည်။ 2. မကောင်းပါ။ |
| 2.4 | သင်တာဝန်ယူထားသော ကျေးရွာ/ လုပ်ငန်းခွင်(များ)တွင် အင်တာနက်အသုံးပြုလို့ရလား? | 1. ရပါသည်။ 2. မရပါ။ **(3.1 သို့သွားပါ)** |
| 2.4.1 | ရတယ်ဆိုရင် အင်တာနက်လိုင်းကောင်းလား? | 1. ကောင်းပါသည်။ 2. မကောင်းပါ။ |
| **အပိုင်း (၃) ငှက်ဖျားသွေးဖောက်စစ်ဆေးခြင်း** | | |
| 3.1 | တစ်လကို ငှက်ဖျားသွေးဖောက်ကိရိယာ (အာရ်ဒီတီ) သုံးပြီး ပျမ်းမျှဘယ်နှစ်ကြိမ်လောက် သွေးစစ်ပေး ရလေ့ရှိလဲ? | ...............ကြိမ် |
| 3.2 | အာရ်ဒီတီဖြင့် ငှက်ဖျားသွေးဖောက်စစ်ဆေးခြင်း ကို တစ်လမှာ ပျမ်းမျှဘယ်နှစ်ရက်လောက် လုပ်ရလေ့ရှိလဲ? | ...............ရက် |
| 3.3 | သင်တာဝန်ယူထားသော ကျေးရွာ/လုပ်ငန်းခွင်(များ)မှာ ငှက်ဖျားသံသယရှိသူများက သင့်ကို/သင်မှ ဘယ်လိုဆက်သွယ်လဲ?  **(တစ်ခုထက်ပို၍ ဖော်ပြနိုင်သည်။)** | 1. လူနာမှ ဖုန်းဆက်ခေါ်သည်။ 2. လူနာ၏မိသားစုဝင်/မိတ်ဆွေများမှ ဖုန်းဆက်အကြောင်းကြားသည် (သို့မဟုတ်) လွှဲအပ်သည်။ 3. လူနာကိုယ်တိုင် လူထုအခြေပြု ဘက်စုံစေတနာ့ဝန်ထမ်း (ငှက်ဖျား) လုပ်သားထံသို့ လာသည်။ 4. လူထုအခြေပြု ဘက်စုံစေတနာ့ဝန်ထမ်း (ငှက်ဖျား)လုပ်သားမှ အိမ်တိုင်ယာရောက် လိုက်လံကြည့်ရှုသည်။ 5. အုပ်စုလိုက် သွေးဖောက်စစ်ဆေးသည်။ 6. အခြား (ဖော်ပြပါ)   ................................................... |
| **အပိုင်း (၄) ငှက်ဖျားလူနာများအား သတင်းပို့ခြင်း** | | |
| 4.1 | သင့်ရပ်ရွာထဲက ငှက်ဖျားလူနာတွေကို ဘယ်လိုနည်းနဲ့ မှတ်တမ်းပြုစုပြီး သတင်းပေးပို့နေလဲ? | 1. PBR တစ်ခုတည်း သုံး၍ **(4.2 သို့ သွားပါ)** 2. MCBR တစ်ခုတည်း သုံး၍ **(4.1.1 သို့ သွားပါ)** 3. PBR နှင့် MCBR နှစ်မျိုးလုံးသုံး၍ **(4.1.2 သို့ သွားပါ)** |
| 4.1.1 | MCBR တစ်မျိုးထဲသုံးတယ်ဆိုရင် အရင် က PBR သုံးပြီး သတင်းပေးပို့ခဲ့ဖူးလား? | 1. သုံးခဲ့ဖူးပါသည်။ 2. မသုံးခဲ့ဖူးပါ။ |
| 4.1.2 | PBR နဲ့ MCBR နှစ်မျိုးလုံးသုံးတယ်ဆိုရင် ဘယ်ဟာကို ပိုသုံးဖြစ်သလဲ? | 1. PBR ကို ပိုသုံးဖြစ်သည်။ 2. MCBR ကို ပိုသုံးဖြစ်သည်။ 3. နှစ်မျိုးလုံး အတူတူသုံးသည်။ 4. အခြား (ဖော်ပြပါ)   ................................................... |
| 4.2 | PBR ကို သက်ဆိုင်ရာအဖွဲ့အစည်းဆီကို ဘယ်လောက်တစ်ခါ ပေးပို့သလဲ? | 1. နေ့စဉ် 2. အပတ်စဉ် 3. နှစ်ပတ်တစ်ခါ 4. လစဉ် 5. အခြား (ဖော်ပြပါ)   ................................................... |
| 4.3 | PBR ကို သက်ဆိုင်ရာအဖွဲ့အစည်းဆီကို ဘယ်လိုပေးပို့သလဲ?  **(တစ်ခုထက်ပို၍ ဖော်ပြနိုင်သည်။)** | 1. ကြီးကြပ်သူမှလာရောက်ကောက်ယူသည်။ 2. မိမိကိုယ်တိုင် ကြီးကြပ်သူထံသွားပို့ရသည်။ 3. ကြီးကြပ်သူထံ လူကြုံဖြင့်ပို့သည်။ 4. လစဉ်အစည်းအဝေးများတွင် ပေးပို့သည်။ 5. သုံးလပတ်အစည်းအဝေးများတွင် ပေးပို့သည်။ 6. အခြား (ဖော်ပြပါ)   ................................................... |
| 4.3.1 | PBR ပေးပို့တာနဲ့ပတ်သက်ပြီး (အဖွဲ့အစည်းထောက်ပံ့ငွေမဟုတ်ပဲ) မိမိကိုယ်ပိုင်ငွေဖြင့် အိတ်စိုက်အသုံးပြုရတာ ရှိသလား? | 1. ရှိပါသည်။ 2. မရှိပါ။ **(4.4 သို့သွားပါ)** |
| 4.3.2 | ရှိပါက ကုန်ကျစရိတ်အမျိုးအစားများကို ဖော်ပြပါ။  **(တစ်ခုထက်ပို၍ ဖော်ပြနိုင်သည်။)** | 1. စာရင်းဖြည့်သွင်းရာတွင် ကုန်ကျခြင်း 2. စာရွက်စာတမ်းများပေးပို့ရာတွင် ကုန်ကျခြင်း 3. အခြား (ဖော်ပြပါ)   ................................................... |
| 4.3.3 | ရှိပါက လစဉ်ကုန်ကျစရိတ်မည်မျှ ရှိသလဲ? | ................................................... ကျပ် |
| 4.4 | MCBR ကို သက်ဆိုင်ရာအဖွဲ့အစည်းဆီကို ဘယ်လောက်တစ်ခါ ပေးပို့သလဲ? | 1. သွေးဖောက်စစ်ဆေးပြီးတိုင်း 2. တစ်ပတ်တစ်ခါစု၍ပို့သည် 3. နှစ်ပတ်တစ်ခါစု၍ပို့သည် 4. တစ်လတစ်ခါစု၍ပို့သည် 5. တစ်လကျော်မှစု၍ ပို့သည် 6. အခြား (ဖော်ပြပါ)   ................................................... |
| 4.5 | MCBR ကို သက်ဆိုင်ရာအဖွဲ့အစည်းဆီကို ဘယ်လိုပေးပို့သလဲ? | 1. မိမိရွာတွင်သာ မိုဘိုင်းဖုန်းဖြင့်ပေးပို့သည်။ 2. အင်တာနက်ဆက်သွယ်မှုရရှိရန် အခြားနေရာသို့ သွား၍ပို့ရသည်။ 3. အခြား (ဖော်ပြပါ)   ................................................... |
| 4.5.1 | MCBR ပေးပို့တာနဲ့ပတ်သက်ပြီး (အဖွဲ့အစည်းထောက်ပံ့ငွေမဟုတ်ပဲ) မိမိကိုယ်ပိုင်ငွေဖြင့် အိတ်စိုက်အသုံးပြုရတာရှိသလား? | 1. ရှိပါသည်။ 2. မရှိပါ။ **(5.1 သို့သွားပါ)** |
| 4.5.2 | ရှိပါက ကုန်ကျစရိတ်အမျိုးအစားများကို ဖော်ပြပါ။  **(တစ်ခုထက်ပို၍ ဖော်ပြနိုင်သည်။)** | 1. မိုဘိုင်းဖုန်းပြင်ဆင်စရိတ် 2. ဖုန်းဘေဖြည့်သည့်စရိတ် 3. ဆက်သွယ်မှုရရန် အခြားနေရာသို့ သွားရသည့် စရိတ် 4. အခြား (ဖော်ပြပါ)   ................................................... |
| 4.5.3 | ရှိပါက လစဉ်ကုန်ကျစရိတ် မည်မျှရှိသလဲ? | ................................................... ကျပ် |
| **အပိုင်း (၅) MCBR နှင့် PBR တို့၏ အသုံးပြုရလွယ်ကူမှု** | | |
| 5.1 | MCBR သုံးရတာ လွယ်သလား? | 1. အလွန်ခက်သည်။ 2. ခက်သည်။ 3. လွယ်လည်းမလွယ် ခက်လည်းမခက်။ 4. လွယ်သည်။ 5. အလွန်လွယ်သည်။ |
| 5.2 | PBR သုံးရတာ လွယ်သလား? | 1. အလွန်ခက်သည်။ 2. ခက်သည်။ 3. လွယ်လည်းမလွယ် ခက်လည်းမခက်။ 4. လွယ်သည်။ 5. အလွန်လွယ်သည်။ |
| 5.3 | PBR နှင့် MCBR နှိုင်းယှဉ်လျှင် မည်သည့်စနစ် ကို သုံးရတာပိုလွယ်သလဲ? | 1. MCBR က ပိုလွယ်ပါသည်။ 2. PBR က ပိုလွယ်ပါသည်။ 3. အတူတူပါပဲ။ |
| **အပိုင်း (၆) လုပ်ငန်းခွင်တွင်ကြုံတွေ့ရသော အကျိုးကျေးဇူးနှင့် အခက်အခဲများ** | | |
| 6.1 | MCBR အသုံးပြုခြင်းရဲ့ အကျိုးကျေးဇူးတွေက ဘာတွေလဲ?  **(တစ်ခုထက်ပို၍ ဖြေဆိုနိုင်သည်။)** | 1. အချိန်မီသတင်းပေးပို့နိုင်ခြင်း 2. မိမိဖုန်းအားအသုံးပြု၍ အချိန်မရွေး (နေ့ညမရွေး) သတင်းပေးပို့နိုင်ခြင်း 3. မိုဘိုင်းဖုန်းတစ်လုံးရှိရုံဖြင့် သတင်းပေးပို့နိုင် သဖြင့် ကရိကထမများခြင်း 4. MCBR အသုံးပြုရန် အဖွဲ့အစည်းမှပေးအပ်ထား သောမိုဘိုင်းဖုန်းကို အသုံးပြု၍ ကြီးကြပ်သူများ နှင့် အလွယ်တကူဆက်သွယ်နိုင်ခြင်း 5. အခြား (ဖော်ပြပါ)   .................................................. |
| 6.2 | PBR အသုံးပြုခြင်းရဲ့ အကျိုးကျေးဇူးတွေက ဘာတွေလဲ?  **(တစ်ခုထက်ပို၍ ဖြေဆိုနိုင်သည်။)** | 1. သုံးနေကျပုံစံဖြစ်သောကြောင့် အထူးလေ့လာရန် မလိုအပ်ခြင်း 2. နည်းပညာအခက်အခဲမရှိခြင်း 3. မှတ်တမ်းများကို အလွယ်တကူ ပြန်လည်ကြည့်ရှုနိုင်ခြင်း 4. မှတ်တမ်းများကို အလွယ်တကူပြင်ဆင်နိုင်ခြင်း 5. အခြား (ဖော်ပြပါ)   ................................................... |
| 6.3 | MCBR သုံးရာမှာ အမြဲလိုလိုကြုံရတဲ့ အဓိကအခက်အခဲတွေက ဘာတွေလဲ? **(တစ်ခုထက်ပို၍ ဖြေဆိုနိုင်သည်။)** | 1. မည်သည့်အခက်အခဲမှ မရှိပါ 2. မိုဘိုင်းဖုန်းမျက်နှာပြင်သေးခြင်း 3. မိုဘိုင်းဖုန်း၏ စွမ်းဆောင်ရည် (RAM) နိမ့်ခြင်း 4. အင်တာနက်လိုင်းမရခြင်း 5. အင်တာနက်လိုင်းမကောင်းခြင်း 6. မြန်မာစာရိုက်ရခက်ခြင်း 7. မိုဘိုင်းဖုန်းကို အကျွမ်းတဝင် မသုံးတတ်ခြင်း 8. အခြားသော လူထုအခြေပြု ဘက်စုံ စေတနာ့ဝန်ထမ်း (ငှက်ဖျား)လုပ်သား အလုပ်များဆောင်ရွက်ရန် အချိန်မရှိခြင်း 9. MCBR အပလီကေးရှင်းသည် မိုဘိုင်းဖုန်း၏ ဘက်ထရီကို မြန်မြန်ကုန်စေသောကြောင့် ဖုန်းကို ခဏခဏ အားပြန်သွင်းရခြင်း 10. အခြား (ဖော်ပြပါ)   ................................................... |
| 6.4 | PBR သုံးရာမှာ အမြဲလိုလိုကြုံရတဲ့ အဓိကအခက်အခဲတွေကဘာတွေလဲ?  **(တစ်ခုထက်ပို၍ ဖြေဆိုနိုင်သည်။)** | 1. မည်သည့်အခက်အခဲမှ မရှိပါ 2. စာရင်းဖြည့်သွင်းရန်အကွက်များသေးငယ်ခြင်း 3. မှားယွင်းဖြည့်သွင်းမိသော စာရင်းများအား ပြင်ဆင်ရန်ခက်ခဲခြင်း 4. အောက်ခံမိတ္တူများတွင် ကောင်းစွာမထင်ခြင်း 5. ရွာနှင့် သတင်းပို့ရသောရုံး ဝေးလံစွာတည်ရှိခြင်း 6. ကာဗွန်လက်စာရွက်များ ပျက်စီး/ပျောက်ဆုံးခြင်း 7. စာရွက်များ သယ်ယူရာတွင် ခက်ခဲခြင်း၊ ဝန်ကျယ်ခြင်း 8. အခြားသော လူထုအခြေပြု ဘက်စုံစေတနာ့ဝန်ထမ်း (ငှက်ဖျား)လုပ်သား အလုပ်များဆောင်ရွက်ရန် အချိန်မရှိခြင်း 9. အခြား (ဖော်ပြပါ)   ................................................... |
| 6.5 | သင်တာဝန်ယူထားသော ကျေးရွာ/လုပ်ငန်းခွင်(များ)မှာ အင်တာနက်မရဘူးဆိုရင် အင်တာနက်အသုံးပြုဖို့ ဘယ်လောက်ဝေးဝေး အထိ သွားရလဲ?  **(အကယ်၍ ရွာတွင်အင်တာနက်ရသည်ဆိုပါက ဤမေးခွန်းအားဖြေဆိုရန် မလိုပါ။)** | ...............မိုင် |
| 6.6 | အဲဒီလိုသွားဖို့ စုစုပေါင်းအချိန်ဘယ်လောက် ကုန်သလဲ? | ..................နာရီ |
| 6.7 | MCBR အသုံးပြုဖို့ ဖုန်းအားသွင်းရန်အတွက် လျှပ်စစ်ဓာတ်အား လုံလုံလောက်လောက် ရသလား? | 1. ရပါသည်။ 2. မရပါ။ |
| 6.8 | ဖုန်းဘေမလောက်လို့ MCBR ကို တသမတ်တည်းမသုံးနိုင်တာမျိုး ရှိခဲ့ဖူးသလား? | 1. ရှိခဲ့ပါသည်။ 2. မရှိခဲ့ပါ။ |
| 6.9 | MCBR ကို ကိုယ့်ဘာသာကိုယ် ပိုက်ဆံစိုက်ပြီး သုံးခဲ့ရတာရှိသလား? | 1. ရှိပါသည်။ 2. မရှိပါ။ |
| **အပိုင်း (၇) MCBR ၏ သတင်းပေးပို့ရာတွင်လုပ်ဆောင်နိုင်စွမ်း** | | |
| 7.1 | MCBR အပလီကေးရှင်းဖွင့်မရတာမျိုး ကြုံဖူးလား? | 1. မကြာခဏကြုံဖူးသည်။ 2. တခါတရံကြုံဖူးသည်။ 3. မကြုံဖူးပါ။ |
| 7.2 | MCBR သုံးနေရင်းနဲ့ အပလီကေးရှင်း သူ့အလိုလို ပိတ်သွားတာ ကြုံဖူးလား? | 1. မကြာခဏကြုံဖူးသည်။ 2. တခါတရံကြုံဖူးသည်။ 3. မကြုံဖူးပါ။ |
| 7.3 | MCBR အပလီကေးရှင်းမှာ ဒေတာသွင်းမရတာမျိုး ကြုံဖူးလား? | 1. မကြာခဏကြုံဖူးသည်။ 2. တခါတရံကြုံဖူးသည်။ 3. မကြုံဖူးပါ။ |
| 7.4 | MCBR အပလီကေးရှင်းမှာ ဒေတာသွင်းပြီး ပို့လွှတ် မရတာမျိုး ကြုံဖူးလား? | 1. မကြာခဏကြုံဖူးသည်။ 2. တခါတရံကြုံဖူးသည်။ 3. မကြုံဖူးပါ။ |
| 7.5 | MCBR အပလီကေးရှင်းနဲ့ပတ်သက်ပြီး ကြုံတွေ့ရတဲ့ အခြားနည်းပညာအခက်အခဲ ရှိလား? | 1. ရှိပါသည်။ 2. မရှိပါ။ **(7.6 သို့သွားပါ)** |
| 7.5.1 | အခြားနည်းပညာအခက်အခဲများကြုံတွေ့ရခြင်း ရှိပါက ဖော်ပြပါ။ |  |
| 7.6 | MCBR အသုံးပြု၍ သတင်းပို့ရာမှာ နှောင့်နှေးခြင်း များကြုံတွေ့ဖူးသလား? | 1. မကြာခဏကြုံဖူးသည်။ 2. တခါတရံကြုံဖူးသည်။ 3. မကြုံဖူးပါ။ **(7.7 သို့သွားပါ)** |
| 7.6.1 | အကယ်၍ နှောင့်နှေးခြင်းများကြုံခဲ့ဖူးပါက ရှင်းပြပါ။ |  |
| 7.7 | သင့်အနေနဲ့ MCBR ၏ သတင်းပေးပို့ရာ၌ လုပ်ဆောင်နိုင်စွမ်းကို ဘယ်လိုအကဲဖြတ်မလဲ? | 1. အလွန်ညံ့ 2. ညံ့ 3. အသင့်အတင့် 4. ကောင်း 5. အလွန်ကောင်း |
| 7.8 | MCBR အပလီကေးရှင်းမှ ဖြည့်သွင်းပြီးဒေတာများကို ပို့လွှတ်ရာတွင် ပြဿနာတစ်စုံတရာ မရှိပါ။ | 1. လုံးဝသဘောမတူပါ။ 2. သဘောမတူပါ။ 3. သဘောတူ/မတူ မသိပါ။ 4. သဘောတူပါသည်။ 5. လုံးဝသဘောတူပါသည်။ |
| 7.9 | မိုဘိုင်းဖုန်းကို ကိုယ်နှင့်မကွာယူဆောင်သွားရ သောကြောင့် တချို့နေရာဒေသများသို့ သွားရာတွင် ဖုန်း လုယက်/ခိုးယူခံရခြင်း၊ ပျောက်ဆုံးခြင်း၊ သဘာဝဘေးအန္တရာယ် ကြောင့်ပျက်စီးခြင်းများအတွက် စိုးရိမ်နေရပါသည်။ | 1. လုံးဝသဘောမတူပါ။ 2. သဘောမတူပါ။ 3. သဘောတူ/မတူ မသိပါ။ 4. သဘောတူပါသည်။ 5. လုံးဝသဘောတူပါသည်။ |
| **အပိုင်း (၈) ငှက်ဖျားပိုးတွေ့လူနာများအား ရှာဖွေကုသညွှန်းပို့အစီရင်ခံရာတွင် PBR နှင့် MCBR တို့၏ အသုံးဝင်ပုံ** | | |
| 8.1 | PBR နှင့်နှိုင်းယှဉ်လျှင် MCBR အသုံးပြုပြီး ငှက်ဖျားလူနာများကို စာရင်းသွင်းရတာပိုလွယ်သည်။ | 1. လုံးဝသဘောမတူပါ။ 2. သဘောမတူပါ။ 3. သဘောတူ/မတူ မသိပါ။ 4. သဘောတူပါသည်။ 5. လုံးဝသဘောတူပါသည်။ |
| 8.2 | PBR နှင့်နှိုင်းယှဉ်လျှင် MCBR အသုံးပြုပြီး ငှက်ဖျားပိုးတွေ့လူနာများကို (၂၄) နာရီအတွင်း သတင်းပို့ရတာ ပိုလွယ်သည်။ | 1. လုံးဝသဘောမတူပါ။ 2. သဘောမတူပါ။ 3. သဘောတူ/မတူ မသိပါ။ 4. သဘောတူပါသည်။ 5. လုံးဝသဘောတူပါသည်။ |
| 8.3 | PBR နှင့်နှိုင်းယှဉ်လျှင် MCBR အသုံးပြုပြီး ပြင်းထန်ငှက်ဖျားလူနာများကို ညွှန်းပို့ရတာပိုလွယ်သည်။ | 1. လုံးဝသဘောမတူပါ။ 2. သဘောမတူပါ။ 3. သဘောတူ/မတူ မသိပါ။ 4. သဘောတူပါသည်။ 5. လုံးဝသဘောတူပါသည်။ |
| 8.4 | PBR နှင့်နှိုင်းယှဉ်လျှင် MCBR အသုံးပြုခြင်းကြောင့်  ငှက်ဖျားသွေးဖောက်စစ်ဆေးခြင်းများကို ပိုမိုလုပ်ဆောင်နိုင်သည်။ | 1. လုံးဝသဘောမတူပါ။ 2. သဘောမတူပါ။ 3. သဘောတူ/မတူ မသိပါ။ 4. သဘောတူပါသည်။ 5. လုံးဝသဘောတူပါသည်။ |
| **အပိုင်း (၉) ဆေးနှင့်ဆေးပစ္စည်းများစီမံခန့်ခွဲရာတွင် PBR နှင့် MCBR တို့၏ အသုံးဝင်ပုံ** | | |
| 9.1 | MCBR ကို ဆေးနှင့်ဆေးပစ္စည်းများ စီမံခန့်ခွဲရန် အသုံးပြုပါသလား? | 1. အသုံးပြုပါသည်။ 2. အသုံးမပြုပါ။ **(10.1 သို့သွားပါ)** |
| 9.2 | PBR နှင့်နှိုင်းယှဉ်လျှင် MCBR ကိုအသုံးပြုပြီး ဆေးနှင့်ဆေးပစ္စည်းပြတ်လပ်တာကို သတင်းပို့ရတာ ပိုလွယ်သည်။ | 1. လုံးဝသဘောမတူပါ။ 2. သဘောမတူပါ။ 3. သဘောတူ/မတူ မသိပါ။ 4. သဘောတူပါသည်။ 5. လုံးဝသဘောတူပါသည်။ |
| 9.3 | PBR နှင့်နှိုင်းယှဉ်လျှင် MCBR ကိုအသုံးပြုပြီး ဆေးနှင့်ဆေးပစ္စည်းလက်ကျန် စစ်ဆေးရတာ ပိုလွယ်သည်။ | 1. လုံးဝသဘောမတူပါ။ 2. သဘောမတူပါ။ 3. သဘောတူ/မတူ မသိပါ။ 4. သဘောတူပါသည်။ 5. လုံးဝသဘောတူပါသည်။ |
| **အပိုင်း (၁၀) MCBR နှင့်ပတ်သက်သော သင်တန်းနှင့်အထောက်အပံ့များ** | | |
| 10.1 | MCBR အသုံးပြုပုံနဲ့ပတ်သက်ပြီး သင်တန်းလုံလုံလောက်လောက် ရခဲ့လား? | 1. ရခဲ့ပါသည်။ 2. မရခဲ့ပါ။ |
| 10.2 | MCBRအသုံးပြုပုံနဲ့ပတ်သက်ပြီး နောက်ထပ်ဘာသင်တန်းတွေထပ်ရချင်လဲ? | 1. မည်သည့်သင်တန်းမှ မလိုအပ်တော့ပါ 2. အခြေခံမိုဘိုင်းဖုန်း အသုံးပြုနည်း 3. ယူနီကုဒ်စနစ်/မြန်မာစာရိုက်စနစ် 4. MCBR အပလီကေးရှင်း အသုံးပြုပုံ 5. အခြား (ဖော်ပြပါ)   ................................................... |
| **အပိုင်း (၁၁) MCBR နှင့်ပတ်သက်သော ခံယူချက်များ** | | |
| 11.1 | MCBR အပလီကေးရှင်းသုံးခြင်းသည် ငှက်ဖျားလူနာသတင်းပို့ရာတွင် လိုအပ်သော အချက်အလက် အားလုံးကို ကောက်ယူနိုင်ရန် အထောက်အကူပြုသည်။ | 1. လုံးဝသဘောမတူပါ။ 2. သဘောမတူပါ။ 3. သဘောတူ/မတူ မသိပါ။ 4. သဘောတူပါသည်။ 5. လုံးဝသဘောတူပါသည်။ |
| 11.2 | MCBR အပလီကေးရှင်းသည် ကျွန်ုပ်၏ လူထုအခြေပြု ဘက်စုံစေတနာ့ဝန်ထမ်း (ငှက်ဖျား)လုပ်သား လုပ်ငန်းစွမ်းဆောင်ရည်ကို တိုးတက်ကောင်းမွန် စေသည်။ | 1. လုံးဝသဘောမတူပါ။ 2. သဘောမတူပါ။ 3. သဘောတူ/မတူ မသိပါ။ 4. သဘောတူပါသည်။ 5. လုံးဝသဘောတူပါသည်။ |
| 11.3 | MCBR သုံးခြင်းကြောင့် စာရင်းအင်းများကို ကြီးကြပ်သူ/သက်ဆိုင်ရာအဖွဲ့အစည်းထံသို့ PBR သုံးခြင်းထက် ပိုမိုလျင်မြန်စွာ ပေးပို့နိုင်သည်။ | 1. လုံးဝသဘောမတူပါ။ 2. သဘောမတူပါ။ 3. သဘောတူ/မတူ မသိပါ။ 4. သဘောတူပါသည်။ 5. လုံးဝသဘောတူပါသည်။ |
| 11.4 | ရွေးလို့ရမယ်ဆိုရင် PBR အစား MCBR ကို ပိုသုံးချင်ပါသည်။ | 1. လုံးဝသဘောမတူပါ။ 2. သဘောမတူပါ။ 3. သဘောတူ/မတူ မသိပါ။ 4. သဘောတူပါသည်။ 5. လုံးဝသဘောတူပါသည်။ |
| 11.5 | ကျွန်ုပ်အနေဖြင့် MCBR ကို ဆက်ပြီးအသုံးပြုဖြစ်မယ်လို့ ထင်ပါသည်။ | 1. လုံးဝသဘောမတူပါ။ 2. သဘောမတူပါ။ 3. သဘောတူ/မတူ မသိပါ။ 4. သဘောတူပါသည်။ 5. လုံးဝသဘောတူပါသည်။ |

**Appendix E: Focus Group Discussion Guide: ICMVs**

**Is it ok to audio record this conversation?** a. Yes b. No

| **Responsible persons** | **Responsibility** |
| --- | --- |
| Facilitator (F) | Lead the overall process and facilitate discussion to obtain enriched data using an ethical approach |
| Note taker (NT) | Note-taking, audio recording and supplementary facilitation |
| Translator | Translation of facilitator and participants discussion where necessary. |

| **Background Information** | | |
| --- | --- | --- |
| 0.1 | Name of F: |  |
| 0.2 | Name of NT: |  |
| 0.3 | Name of Translator: |  |
| 0.4 | Date (DD/MM/YYYY): |  |
| 0.5 | Start time: |  |
| 0.6 | End time: |  |
| 0.7 | Location (venue): |  |
| 0.8 | Archival code |  |
| 0.9 | Sex of participants (group) |  |
| 1.0 | Age range of participants |  |

**Themes, questions and probes**

| **No** | **Themes** | **Questions and probes** |
| --- | --- | --- |
| 1 | Current malaria situation | - What is the burden of malaria in your working village/area?   - - Is it increasing or decreasing in your areas? - How was it impacted on your village/community? - How did you manage to help the malaria patients? - Do you think malaria is still a priority health issue in your village/community?   - - Why do you think so? |
| 2 | Malaria control measures and available malaria provider(s) in the respective community | - What are the available services/interventions for malaria in your community?   - - Do you think are they enough to combat malaria?     - If not, what interventions are you going to suggest? - Where does your community get the malaria interventions/services recently? Who are the malaria service provider(s)? Is it only you? Are there more malaria service providers?   - - Do you think this/these providers fulfil the malaria service gap in the community?     - If not, why and what’s your suggestion to meet the needs?     - Are they also overlapping each other in the malaria service provision? |
| 3 | Malaria elimination | - Have you heard ‘malaria elimination’?   - If yes, what does it mean to you?   - What do you think your role will be in the malaria elimination program? |
| 4 | Reporting of malaria cases | - Can you tell me your experience of the reporting of malaria cases since you have been working as a volunteer? Please give examples.   - How have you reported the cases?   - Only by paper based reporting?     - If so, what are the barriers and enablers of PBR in the past? Please give examples. - Have you ever noticed any barriers and/or enablers to timely reporting of malaria cases in the community currently?   - - What are they? Please give examples.     - How do we enhance the enablers in the future? Please give examples.     - How do we overcome the barriers in the future? Please give examples.     - What kinds of support are necessary to overcome the barriers and from whom? |
| 5. | Stock management | - As a volunteer, can you explore experience of stock management? - How did you manage? Are there any problems in particular, working as an ICMV? - How has the carbonless paper helped (or not) in your stock management? Please give examples. - How can we overcome the stock management issues and which type of supports are needed, and from whom? - Any suggestions for future ICMV stock management? |
| 6 | General views and perspectives on the current PBR and MCBR | - Are you still willing to continue using PBR?   - If so, why? - Do you know MCBR? Please describe it.   - What is the difference between MCBR compared to PBR? Please give examples. - Do you think MCBR is an effective system for reporting malaria cases   - - Why? And How? Please give examples.     - What are the enablers of using MCBR application? - Do you intended to regularly use MCBR instead of PBR?   - - If yes, what influences your decision to use MCBR over PBR - What are the specific barriers and enablers of using MCBR? Please give examples.   - Ease of use   - Data lost   - Sync error   - Bill for the use of online reporting   - If yes, why?   - If not, why? |
| 7 | MCBR application | - How do you think the MCBR could be improved in future? - What features and/or functions do you want to add or remove into/from MCBR? Please explain one by one. - How can we best put MCBR application in the context of ICMV? - Do you think MCBR should extend to reporting of other ICMV diseases? Why? - If we expand MCBR for reporting of other diseases, what kind of supports for ICMV will be needed? And from whom? Why? |
| 8 | Training and support for MCBR | - To what extent were you satisfied with the training you received on how to use the MCBR? - What further training would be of benefit. - What supports should be provided to volunteers if the volunteers have to use only MCBR for reporting. Please explain detail.   - Financial, in kind and/or moral supports |
| 9 | Overall | - Overall, do you think PBR can be replaced by MCBR for reporting of malaria cases   - Why do you think so? |

This is the end of my questions. Do you have anything else you would like to say about this research?

Do you have any questions for me?

Thank you very much for your participation.

**End of session**

**ငှက်ဖျားရောဂါဆိုင်ရာ သတင်းအချက်အလက်များပေးပို့ရာတွင် မိုဘိုင်းအပလီကေရှင်း (MCBR) သုံးခြင်းနှင့် ကာဗွန်လက်စ် ငှက်ဖျားလချုပ်များသုံးခြင်း (PBR) တို့၏ ထိရောက်မှုကို နှိုင်းယှဉ်လေ့လာခြင်း**

**လူထုအခြေပြု ဘက်စုံစေတနာ့ဝန်ထမ်း (ငှက်ဖျား)လုပ်သား (ICMV)များနှင့် ဦးတည်အုပ်စုဖွဲ့ဆွေးနွေးခြင်းလမ်းညွှန်**

ယခု ဆွေးနွေးပွဲကို အသံသွင်းထားရန် ခွင့်ပြုပါသလား? (1) ပြုပါသည်။ (2) မပြုပါ။

| **သုတေသနအဖွဲ့ဝင်** | **အခန်းကဏ္ဍ** |
| --- | --- |
| ဦးဆောင်ဆွေးနွေးသူ | ဦးတည်အုပ်စုဖွဲ့ဆွေးနွေးခြင်းတစ်ခုလုံးကို ဦးဆောင်ကြီးကြပ်ရန်၊ လိုအပ်သည့် အချက်အလက်များ ရရှိရန်အတွက် ကျင့်ဝတ်နှင့်အညီ ကူညီဆွေးနွေးရန်။ |
| မှတ်တမ်းတင်သူ | မှတ်စုရေးခြင်း၊ အသံသွင်းခြင်းနှင့် အခြားလိုအပ်သည့်နေရာများ၌ ကူညီ အားဖြည့်ရန်။ |
| စကားပြန် | ဦးဆောင်ဆွေးနွေးသူနှင့် လူထုအခြေပြု ဘက်စုံစေတနာ့ဝန်ထမ်း (ငှက်ဖျား) လုပ်သားများ အကြား လိုအပ်သလိုဘာသာြပန်ဆိုပေးရန်။ |

| **အခြေခံအချက်အလက်များ** | | |
| --- | --- | --- |
| 0.1 | ဦးဆောင်ဆွေးနွေးသူအမည် |  |
| 0.2 | မှတ်တမ်းတင်သူအမည် |  |
| 0.3 | စကားပြန်အမည် |  |
| 0.4 | ရက်စွဲ (ရက်/လ/နှစ်) |  |
| 0.5 | စတင်ချိန် |  |
| 0.6 | ပြီးဆုံးချိန် |  |
| 0.7 | နေရာ |  |
| 0.8 | မော်ကွန်းအမှတ်စဉ် |  |
| 0.9 | ဖြေဆိုသည့်အုပ်စု၏လိင် (ကျား/မ) |  |
| 0.10 | ဖြေဆိုသူများ၏ အသက်အပိုင်းအခြား |  |

**Themes, questions and probes**

| **(၁) လက်ရှိငှက်ဖျားအခြေအနေ** |
| --- |
| - 1. သင်အလုပ်လုပ်နေသောကျေးရွာ/ဒေသမှာ ငှက်ဖျားအခြေအနေဘယ်လိုရှိသလဲ?  1. အရင်ကထက် များလာသလား၊ နည်းသွားသလား    1. ငှက်ဖျားဖြစ်ပွားမှုများလာခြင်း(သို့မဟုတ်)နည်းလာခြင်းကသင့်ကျေးရွာ/အရပ်ဒေသအပေါ်မှာ ဘယ်လို အကျိုးသက်ရောက်မှုရှိသလဲ?    2. ငှက်ဖျားပိုးတွေ့လူနာများကို သင်ဘယ်လိုကူညီဆောင်ရွက်ပေးလဲ?    3. သင့်ကျေးရွာ/အရပ်ဒေသမှာငှက်ဖျားသည်အရေးကြီးသည့်ကျန်းမာရေးပြဿနာတစ်ရပ်လား? 2. ဘာကြောင့်လဲ? |
| **(၂) ငှက်ဖျားတိုက်ဖျက်ရေးလုပ်ငန်းစဉ်များနှင့် သက်ဆိုင်ရာဒေသတွင်းရှိ ငှက်ဖျားနှင့်ပတ်သက်သော ကျန်းမာရေးဝန်ဆောင်မှုများ** |
| - 1. သင့်ရပ်ရွာဒေသမှာ ငှက်ဖျားနှင့်ပတ်သက်တဲ့ ဘာဝန်ဆောင်မှုတွေရှိသလဲ?  1. အဲဒီဝန်ဆောင်မှုတွေက ငှက်ဖျားရောဂါတိုက်ဖျက်ရေးမှာ လုံလောက်ပြီဟုသင်ထင်ပါသလား? 2. အကယ်၍ မလုံလောက်သေးဘူးဆိုရင် ဘာတွေထပ်လုပ်ဖို့ အကြံပေးချင်လဲ?    1. ဒေသခံတွေအနေနဲ့ ငှက်ဖျားနဲ့ပတ်သက်တဲ့ ဝန်ဆောင်မှုတွေကို ဘယ်နေရာက ရနေသလဲ? ဤဒေသမှာ ငှက်ဖျားနဲ့ပတ်သက်ပြီး ကျန်းမာရေးဝန်ဆောင်မှုပေးနေသူတွေက ဘယ်သူတွေလဲ? သင်တစ်ယောက်ပဲ ရှိတာလား? အခြားရော ဘယ်သူတွေရှိသေးလဲ? 3. ဒီဒေသမှာ လက်ရှိဝန်ဆောင်မှုပေးသူတွေနဲ့ လုံလောက်ပြီလို့ သင်ထင်သလား? 4. မလုံလောက်သေးဘူးဆိုရင် လိုအပ်ချက်တွေပြည့်မီဖို့ ဘာတွေလုပ်သင့်တယ်လို့ သင်ထင်သလဲ? 5. ငှက်ဖျားနဲ့ပတ်သက်တဲ့ ဝန်ဆောင်မှုပေးတဲ့နေရာမှာ တစ်ယောက်နဲ့တစ်ယောက် လုပ်ငန်းပုံစံခြင်း ထပ်နေတာရှိသလား? |
| **(၃) ငှက်ဖျားကင်းဝေးရေး** |
| - 1. ငှက်ဖျားကင်းဝေးရေးဆိုတာ ကြားဖူးလား?   2. ကြားဖူးသည်ဆိုပါက ဘယ်လိုနားလည်ထားသလဲ?   3. ငှက်ဖျားကင်းဝေးရေးဆောင်ရွက်ရာမှာ သင့်အခန်းကဏ္ဍကဘာလို့ထင်လဲ? |
| **(၄) ငှက်ဖျားပိုးတွေ့လူနာများအား သတင်းပို့ခြင်း** |
| - 1. စေတနာ့ဝန်ထမ်းတစ်ယောက်အနေနဲ့ ငှက်ဖျားရောဂါဆိုင်ရာ သတင်းအချက်အလက်များပေးပို့ခဲ့တဲ့ အတွေ့အကြုံတွေကို ပြောပြပေးနိုင်မလား? ဥပမာပေး ပေးပါ။  1. သင်ဘယ်လို သတင်းပို့ခဲ့သလဲ? 2. ကာဗွန်လက်စ်ငှက်ဖျားလချုပ်နဲ့ပဲ ပို့ခဲ့တာလား? အရင်က ကာဗွန်လက်စ်ငှက်ဖျားလချုပ်ပဲသုံးခဲ့တုန်းက ဘာအခက်အခဲတွေရှိခဲ့လဲ? ဥပမာပေး ပေးပါ။    1. လောလောဆယ် ငှက်ဖျားပိုးတွေ့လူနာတွေကို အချိန်နဲ့တပြေးညီသတင်းပို့ရာမှာ ဘာအခက်အခဲတွေ သတိထားမိသလဲ? 3. အဲဒီအခက်အခဲတွေက ဘာတွေလဲ? ဥပမာပေး ပေးပါ။ 4. အခက်အခဲတွေကို ဘယ်လိုကျော်လွှားမလဲ? အခက်အခဲတွေကိုကျော်လွှားဖို့ ဘယ်သူတွေဆီက ဘာအထောက်အပံ့တွေ လိုမလဲ? 5. အနာဂတ်မှာ ပိုကောင်းအောင် ဘာတွေလုပ်သင့်လဲ? ဥပမာပေး ပေးပါ။ |
| **(၅) ဆေးနှင့်ဆေးပစ္စည်းများစီမံခန့်ခွဲခြင်း** |
| - 1. လူထုအခြေပြု ဘက်စုံစေတနာ့ဝန်ထမ်း (ငှက်ဖျား)လုပ်သားတစ်ယောက်အနေနဲ့ ဆေးနှင့်ဆေးပစ္စည်းများ စီမံခန့်ခွဲတဲ့ အတွေ့အကြုံကို ပြောပြပေးပါ။   2. ဆေးနှင့်ဆေးပစ္စည်းများကို သင် ဘယ်လို စီမံခန့်ခွဲခဲ့လဲ? လူထုအခြေပြု ဘက်စုံစေတနာ့ဝန်ထမ်း (ငှက်ဖျား)လုပ်သားတစ်ယောက်အဖြစ် လုပ်ကိုင်နေစဉ် ဆေးနှင့်ဆေးပစ္စည်းနဲ့ပတ်သက်ပြီး အခက်အခဲ ပြဿနာတစ်စုံတစ်ရာ ကြုံတွေ့ခဲ့ရဖူးလား?   3. ကာဗွန်လက်စ်ငှက်ဖျားလချုပ်က သင် ဆေးနှင့်ဆေးပစ္စည်းများစီမံခန့်ခွဲရာမှာ ဘယ်လို အကူအညီပေးသလဲ? ဥပမာ ပေး ပေးပါ။   4. ဆေးနှင့်ဆေးပစ္စည်းစီမံခန့်ခွဲတာနဲ့ပတ်သက်တဲ့ ပြဿနာတွေကို ဘယ်လိုဖြေရှင်းနိုင်မလဲ? အဲဒီလို ဖြေရှင်းနိုင်ဖို့ ဘယ်သူ့ဆီက ဘယ်လိုအထောက်အပံ့တွေလိုမလဲ?   5. အနာဂတ်မှာ လူထုအခြေပြု ဘက်စုံစေတနာ့ဝန်ထမ်း (ငှက်ဖျား)လုပ်သားတွေရဲ့ ဆေးနှင့်ဆေးပစ္စည်း စီမံခန့်ခွဲတာနဲ့ပတ်သက်ပြီး ဘာများအကြံပြုချင်တာရှိသလဲ? |
| **(၆) ယခုလက်ရှိအသုံးပြုနေသော MCBR အပလီကေးရှင်းနှင့် ကာဗွန်လက်စ်ငှက်ဖျားလချုပ်အပေါ် ယေဘူယျ အမြင်** |
| - 1. ကာဗွန်လက်စ်ငှက်ဖျားလချုပ်ကို ဆက်လက်အသုံးပြုလိုပါသလား?   2. ဘာကြောင့်လဲ?   3. MCBR ဆိုတာကို သိပါသလား? ကျေးဇူးပြု၍ ၎င်း MCBR အကြောင်းကိုဖော်ြပပါ။  1. MCBR နှင့် ကာဗွန်လက်စ်ငှက်ဖျားလချုပ် ဘာကွာြခားသလဲ? ဥပမာပေး ပေးပါ။    1. ငှက်ဖျားရောဂါဆိုင်ရာသတင်းအချက်အလက်များပေးပို့ရာမှာ MCBR သည် ထိရောက်မှုရှိသော စနစ်တစ်ခု ဖြစ်သည်ဟု သင်ထင်ပါသလား? 2. ဘာကြောင့်လဲ? ဘယ်လိုကြောင့်လဲ? ဥပမာပေး ပေးပါ။ 3. MCBR အသုံးပြုခြင်းရဲ့ အားသာချက်တွေကဘာတွေလဲ?    1. ကာဗွန်လက်စ်ငှက်ဖျားလချုပ်အစား MCBRကို ပုံမှန်သုံးမည်ဟု သင် ရည်မှန်းထားပါသလား? 4. ရည်မှန်းထားသည်ဆိုပါက ဘာအကြောင်းတွေကြောင့် ကာဗွန်လက်ငှက်ဖျားလချုပ်အစား MCBR ကို အစားထိုးသုံးစွဲမည်ဟု ဆုံးဖြတ်ခဲ့သလဲ?    1. MCBR သုံးစွဲရာမှာကြုံတွေ့ရတဲ့ အခက်အခဲနှင့် အားသာချက်တွေကဘာတွေလဲ? ဥပမာပေး ပေးပါ။ 5. သုံးစွဲရလွယ်ကူခြင်း 6. သတင်းအချက်အလက်များပျောက်ဆုံးခြင်း 7. ဖြည့်သွင်းပြီးအချက်အလက်များပို့လွှတ်ရာ၌ ပြဿနာရှိခြင်း 8. သတင်းပို့ရန် ဖုန်းဘေလ်လိုအပ်ချက်    - ဘာကြောင့်လဲ? ရှင်းပြပါ။ |
| **(၇)** **MCBR အပလီကေးရှင်း** |
| - 1. နောင်တွင် MCBR အပလီကေးရှင်းကိုပိုမိုကောင်းမွန်အောင်မည်ကဲ့သို့ဆောင်ရွက်သင့်သည်ဟု သင်ထင် သလဲ?   2. MCBR အပလီကေးရှင်းတွင် မည်သည့်လုပ်ဆောင်ချက်များ ထပ်ဖြည့်/ဖယ်ထုတ်သင့်သည်ဟု သင်ထင် သလဲ? တစ်ခုချင်းရှင်းပြပေးပါ။   3. MCBR အပလီကေးရှင်းကို လူထုအခြေပြု ဘက်စုံစေတနာ့ဝန်ထမ်း (ငှက်ဖျား)လုပ်သားတွေအတွက် ဘယ်လိုအကောင်းဆုံးအသုံးပြုနိုင်မလဲ?   4. MCBR အပလီကေးရှင်းကို ၎င်းတို့ရဲ့ ငှက်ဖျားမဟုတ်တဲ့ အခြား ရောဂါတွေကို သတင်းပို့ရာမှာအသုံးပြုဖို့ တိုးချဲ့သင့်တယ်လို့ထင်ပါသလား? ဘာကြောင့်လဲ?   5. အကယ်၍ MCBR အပလီကေးရှင်းကို ငှက်ဖျားမဟုတ်တဲ့အခြားရောဂါတွေကို သတင်းပို့ရာမှာအသုံး ပြုဖို့ တိုးချဲ့မယ်ဆိုရင် လူထုအခြေပြု ဘက်စုံစေတနာ့ဝန်ထမ်း (ငှက်ဖျား)လုပ်သားတွေအတွက် ဘယ်သူတွေဆီက ဘာအထောက်အပံ့တွေလိုမလဲ? ဘာကြောင့်လဲ? |
| **(၈)** **MCBR အတွက် သင်တန်းနှင့် အထောက်အပံ့များ** |
| - 1. သင် တက်ရောက်ခဲ့တဲ့MCBRအသုံးပြုနည်းသင်တန်းအပေါ် ဘယ်အတိုင်းအတာအထိ ကျေနပ်မှု ရှိသလဲ?   2. နောင်တွင် MCBR အတွက် ဘယ်လိုသင်တန်းတွေပေးရင် အကျိုးရှိမယ်လို့ ထင်သလဲ?   3. ငှက်ဖျားရောဂါဆိုင်ရာ သတင်းအချက်အလက်များပေးပို့ရာမှာ MCBR တစ်မျိုးထဲအသုံးပြုမယ်ဆိုရင် လူထုအခြေပြု ဘက်စုံစေတနာ့ဝန်ထမ်း (ငှက်ဖျား)လုပ်သားတွေကို ဘာအထောက်အပံ့တွေ ပေးသင့်သလဲ? အသေးစိတ်ရှင်းပြပါ။   4. ဥပမာ-ငွေကြေးအထောက်အပံ့၊ ငွေကြေးမဟုတ်သောအခြားအထောက်အပံ့များ စသည်ဖြင့် |
| **(၉) ခြုံငုံသုံးသပ်ခြင်း** |
| - 1. အားလုံးကိုခြုံငုံသုံးသပ်ရင် ငှက်ဖျားရောဂါဆိုင်ရာသတင်းအချက်အလက်များပေးပို့ရာမှာ MCBR အပလီ ကေးရှင်းက ကာဗွန်လက်စ်ငှက်ဖျားလချုပ်ကို အစားထိုးနိုင်လိမ့်မယ်လို့ ထင်ပါသလား? ဘာကြောင့်လဲ? |

မေးခွန်းများမေးမြန်းခြင်းပြီးပါပြီ။ ဤသုတေသနနှင့်ပတ်သက်၍ မေးမြန်းလိုသည်များရှိပါသလား?

အခြားမေးမြန်းလိုသည်များ ရှိပါသလား?

အချိန်ပေး၍ ပါဝင်ဖြေဆိုပေးသည့်အတွက် ကျေးဇူးအထူးတင်ရှိပါသည်။

**Appendix F: In-depth Interview Guide: Implementing Partners**

**Is it ok to audio record this conversation?** a. Yes b. No

| **Background Information** | | |  |
| --- | --- | --- | --- |
| 0.1 | Name of interviewer: | ____________________ |  |
| 0.2 | Date (DD/MM/YYYY): | ________ |  |
| 0.3 | Start time: | ________ |  |
| 0.4 | End time: | ________ |  |
| 0.5 | Job level (indicate): | Higher level/ Middle level policy maker |  |
| 0.6 | Organization (indicate): | SCI/HPA/IOM/ NMCP |  |
| 0.7 | How long have you worked in your current position (in years)? | ________ |  |
| 0.8 | How many years of work experience in malaria sector do you have (in total)? | ________ |  |
| **Section 1: Roles and responsibilities** | | |  |
|  | Describe your current role and key responsibilities |  |  |
| **Section 2: Current malaria situation** | | |  |
|  | What’s the situation of malaria in your assigned territory? |  |  |
|  | Why do you think that the burden of malaria in your area is going up/down or plateauing? |  |  |
| **Section 3: Reporting in malaria program** | | |  |
|  | Which reporting systems are being used for malaria cases and malaria interventions from ICMVs? (PBR, MCBR, ph call, social media, viber, etc.) |  |  |
|  | Can you please elaborate pros and cons of using each reporting system for the program? (Pros and Cons) |  |  |
|  | Which reporting system do you think is the most effective? |  |  |
|  | Do you think it is possible for this system to be scaled-up for use in Myanmar? |  |  |
| **Section 4: Views and perspectives on paper-based reporting (PBR)** | | |  |
|  | Can you describe the paper based reporting system for malaria case reporting? |  |  |
|  | Do you think PBR is an effective method of malaria surveillance in an elimination setting? Why? If not, why? |  |  |
|  | How do you receive and access the data collected by ICMVs using PBR? |  |  |
|  | How do you analyse the data collected by PBR? |  |  |
|  | What are barriers / challenges you experience in using PBR? |  |  |
|  | How do you think we can mitigate these barriers? |  |  |
|  | What barriers / challenges do you experience in implementing / managing the PBR reporting system? |  |  |
|  | How do you think we can mitigate these barriers? |  |  |
|  | What barriers / challenges do you think ICMVs using PBR would encounter? |  |  |
|  | How do you think we can mitigate these barriers? |  |  |
|  | What are some of the facilitators that enable ICMVs to use PBR? |  |  |
|  | What are reasons for why you are using PBR systems compared to other systems such as MCBR? |  |  |
|  | What are the advantages to using in PBR systems compared to MCBR?  For Case Detection vs. Reporting |  |  |
|  | Are you happy with the data quality of the PBR systems?  Why/ Why not? |  |  |
| **Section 5: Views and perspectives on MCBR** | | |  |
|  | Can you describe the MCBR system for malaria case reporting? |  |  |
|  | Do you think MCBR is an effective method of malaria surveillance in an elimination setting? Why? If not, why? |  |  |
|  | How do you receive and access the data collected by VHVs using MCBR? |  |  |
|  | How do you analyse the data collected by MCBR? |  |  |
|  | What are barriers / challenges you experience in using MCBR? |  |  |
|  | How do you think we can mitigate these barriers? |  |  |
|  | What barriers / challenges do you experience in implementing / managing the MCBR reporting system? |  |  |
|  | How do you think we can mitigate these barriers? |  |  |
|  | What barriers / challenges do you think VHVs using the MCBR would encounter? |  |  |
|  | How do you think we can mitigate these barriers? |  |  |
|  | What are some of the facilitators that enable VHVs to use MCBR? |  |  |
|  | What are reasons for why you (MoH / IP Stakeholders) are using MCBR systems compared to other to systems such as PBR? |  |  |
|  | What are the advantages to using in MCBR systems compared to PBR?  For Case Detection vs. Reporting |  |  |
|  | What is the most useful part of the MCBR system? Why? |  |  |
|  | To what extent does the MCBR improve your access to reporting data? |  |  |
|  | Are you happy with the data quality of the MCBR systems?  Why/ Why not? |  |  |
| **Section 6: Usability of MCBR** | | |  |
|  | How do you think the MCBR system could be improved? |  |  |
|  | How do you currently use the data from MCBR /DHIS2 to inform decision making around funding/resources allocation? |  |  |
|  | What is the most challenging part of accessing and/or using data gathered through MCBR/ DHIS2? Why? |  |  |
|  | What type of capability could be added to the MCBR software to help you with your work? |  |  |
|  | What concerns do you have in using/scaling up MCBR? |  |  |
| **Section 7: Supports needed for applying MCBR in malaria program** | | |  |
|  | Which supports are needed for ICMVs to expand the MCBR as a routine and effective reporting channel?  (technical and training, financial, technological, political, advocacy, administrative, moral etc)  Are they been fulfilled?  If not, do you see any plan to fulfil those requirements?  If not, how can we best fulfil those requirements in time? |  |  |
|  | Which supports are needed for data assistants, M&E staff and/or any data staff who have responsibility to compile, enter and analyse data from MCBR? (technical and training, financial, technological, political, advocacy, administrative, moral etc)  Are they been fulfilled?  If not, do you see any plan to fulfil those requirements?  If not, how can we best fulfil those requirements in time? |  |  |
|  | Which supports are needed for policy makers and/or decision makers to access and utilize data from MCBR? (technical and training, financial, technological, political, advocacy, administrative, moral etc)  Are they been fulfilled?  If not, do you see any plan to fulfil those requirements?  If not, how can we best fulfil those requirements in time? |  |  |
| **Section 8: Sustainability in the context of ICMV** | | |  |
|  | Do you think MCBR application is sustainable in the context of ICMV if necessary improvements are being made and/or necessary supports are provided? Why? Why not? |  |  |
|  | What are the key factors for the sustainability of MCBR in reporting in malaria program? How do we address them to sustain the MCBR in long run? |  |  |
|  | Do you think NMCP will be able to maintainthe operation of MCBR without support from Save the Children? Why? Please discuss detail including the following discussion points:  Server maintenance, server expansion, safeguarding data. |  |  |
|  | What kinds of adaptations need to be made to the application and overall system to enable NMCP to maintain operation of MCBR? |  |  |
|  | What kinds of resources are needed to enable NMCP to maintain operation of MCBR? |  |  |
|  | Can we use app-based reporting channel for reporting of non-malaria ICMV diseases? |  |  |
|  | What kinds of adaptations need to be made to the application and overall system to enable reporting of non-malaria disesase covered by ICMV? |  |  |
|  | What kind of resources are needed to enable reporting of non-malaria disesase covered by ICMV? |  |  |
|  | Who will be using the app-based reporting channel (BHS, Midwife, etc.)? Please discuss. |  |  |
|  | What kinds of adaptations need to be made to the application and overall system to enable non-ICMV personnel (e.g. BHS, midwives) to use the application? |  |  |
|  | What kind of resources are needed to enable non-ICMV personnel (e.g. BHS, midwives) to use the application? |  |  |
| **Section 9: Overall** | | |  |
|  | Do you think PBR can be totally replaced by MCBR for reporting of malaria cases in Myanmar malaria elimination program? Why /Why not?  If yes, how can we best transition from PBR to MCBR? What are the factors that need to be taken care off in the transition? |  |  |
|  | Do you have any other suggestions/ comments regarding the reporting of malaria cases? If yes, please make comments. |  |  |

Do you have any questions for me?

Thank you very much for your participation.

**End of session**

**ငှက်ဖျားရောဂါဆိုင်ရာ သတင်းအချက်အလက်များပေးပို့ရာတွင် မိုဘိုင်းအပလီကေရှင်း (MCBR) သုံးခြင်းနှင့် ကာဗွန်လက်စ် ငှက်ဖျားလချုပ်များသုံးခြင်း (PBR) တို့၏ ထိရောက်မှုကို နှိုင်းယှဉ်လေ့လာခြင်း**

**အားကစားနှင့်ကျန်းမာရေးဝန်ကြီးဌာနမှအရာရှိများနှင့် မိတ်ဘက်အဖွဲ့အစည်းများမှဝန်ထမ်းများအား တစ်ဦးချင်းအသေးစိတ်ဆွေးနွေးမေးမြန်းခြင်းလမ်းညွှန်**

ယခု ဆွေးနွေးပွဲကို အသံသွင်းထားရန် ခွင့်ပြုပါသလား? (1) ပြုပါသည်။ (2) မပြုပါ။

| **အခြေခံအချက်အလက်များ** | | |
| --- | --- | --- |
| 0.1 | တွေ့ဆုံမေးမြန်းသူ၏အမည် |  |
| 0.2 | ရက်စွဲ (ရက်/လ/နှစ်) |  |
| 0.3 | စတင်ချိန် |  |
| 0.4 | ပြီးဆုံးချိန် |  |
| 0.5 | ရာထူးအဆင့် (ဖော်ြပပါ): | အမြင့်တန်း/ အလယ်တန်း အဆင့် အကောင်အထည် ဖော် ဆောင်ရွက်သူ |
| 0.6 | အဖွဲ့အစည်းအမည် (ဖော်ြပပါ) | SCI/HPA/IOM/NMCP |
| 0.7 | သင် ယခုရာထူးမှာတာဝန်ထမ်းဆောင်နေတာ ဘယ်လောက်ကြာပြီ လဲ? | ..................နှစ် |
| 0.8 | သင့်မှာ ငှက်ဖျားနဲ့ပတ်သက်ပြီး လုပ်ငန်းအတွေ့အကြုံစုစုပေါင်း ဘယ်လောက်ရှိသလဲ? | ..................နှစ် |

| **(၁) အခန်းကဏ္ဍနှင့်တာဝန်ယူမှု** |
| --- |
| - 1. သင့်ရဲ့ လက်ရှိ အခန်းကဏ္ဍနဲ့ တာဝန်ယူရတဲ့အပိုင်းတွေကို ပြောပြပေးပါ။ |
| **(၂) ယခုလက်ရှိ ငှက်ဖျားအခြေအနေ** |
| - 1. သင် တာဝန်ကျတဲ့ဒေသရဲ့ ငှက်ဖျားအခြေအနေကို ပြောပြပေးပါ။   2. ဘာကြောင့် သင်တာဝန်ကျတဲ့ဒေသမှာငှက်ဖျားရောဂါအခြေအနေကောင်းမွန်လာ/ဆိုးရွားလာ/ဒီအတိုင်းပဲ တန့်နေတယ်လို့ ထင်သလဲ? |
| **(၃) ငှက်ဖျားရောဂါဆိုင်ရာ သတင်းအချက်အလက်များပေးပို့ခြင်း** |
| - 1. လူထုအခြေပြု ဘက်စုံစေတနာ့ဝန်ထမ်း (ငှက်ဖျား)လုပ်သားများထံမှ ငှက်ဖျားရောဂါဆိုင်ရာ သတင်း အချက်အလက်တွေရဖို့ ဘယ်စနစ်တွေအသုံးပြုနေလဲ? (ကာဗွန်လက်စ်ငှက်ဖျားလချုပ်၊ MCBR၊ ဖုန်းခေါ် ခြင်း၊ လူမှုကွန်ယက်များ၊ Viber အစရှိသဖြင့်)   2. သတင်းအချက်အလက်ပို့တဲ့စနစ်တစ်ခုချင်းစီရဲ့ ကောင်းကျိုးဆိုးပြစ်တွေကိုနည်းနည်းလောက်အကျယ်ချဲ့ ပြီး ပြောပြပေးမလား?   3. ဘယ် သတင်းအချက်အလက်ပို့တဲ့စနစ်က စွမ်းဆောင်ရည်အကောင်းဆုံးလို့ထင်သလဲ?   4. အဲဒီစနစ်ကို မြန်မာတစ်နိုင်ငံလုံးမှာ တိုးချဲ့အသုံးပြုဖို့ ဖြစ်နိုင်မယ်ထင်သလား? |
| **(၄) ကာဗွန်လက်စ်ငှက်ဖျားလချုပ်စနစ်အပေါ်အမြင်နှင့်ယူဆချက်များ** |
| - 1. ကာဗွန်လက်စ်ငှက်ဖျားလချုပ်စနစ် အကြောင်း ပြောပြပေးပါ။   2. ကာဗွန်လက်စ်ငှက်ဖျားလချုပ်ဟာ မြန်မာနိုင်ငံရဲ့ငှက်ဖျားကင်းဝေးရေးလုပ်ငန်းမှာ ထိရောက်တဲ့ငှက်ဖျား စောင့်ကြပ်ကြည့်ရှုရေးနည်းလမ်းတစ်ခုဖြစ်တယ်လို့ သင့်အနေနဲ့ ထင်မြင်ယူဆပါသလား? ဘာကြောင့်လဲ?   3. ကာဗွန်လက်စ်ငှက်ဖျားလချုပ်နဲ့ကောက်ယူထားတဲ့အချက်အလက်တွေ ကို သင် ဘယ်လိုနည်းနဲ့ ရယူသိရှိ နိုင်သလဲ?   4. ကာဗွန်လက်စ်ငှက်ဖျားလချုပ်နဲ့ကောက်ယူထားတဲ့အချက်အလက်တွေကို သင် ဘယ်လိုခွဲခြမ်းလေ့လာ သုံးသပ်သလဲ?   5. ကာဗွန်လက်စ်ငှက်ဖျားလချုပ်စနစ်ကို သင် အသုံးပြုတဲ့နေရာမှာရင်ဆိုင်ရတဲ့ အတားအဆီးနဲ့စိန်ခေါ်မှုတွေ က ဘာတွေလဲ?  1. အဲဒီအတားအဆီးတွေကို ဘယ်လိုကျော်လွှားနိုင်မယ်လို့ သင်ထင်သလဲ?    1. ကာဗွန်လက်စ်ငှက်ဖျားလချုပ်စနစ်ကို သင် အကောင်အထည်ဖော်ဆောင်၊ စီမံခန့်ခွဲတဲ့နေရာမှာ ရင်ဆိုင်ရ တဲ့ အတားအဆီးနဲ့စိန်ခေါ်မှုတွေက ဘာတွေလဲ? 2. အဲဒီအတားအဆီးတွေကို ဘယ်လိုကျော်လွှားနိုင်မယ်လို့ သင်ထင်သလဲ?    1. ကာဗွန်လက်စ်ငှက်ဖျားလချုပ်သုံးတဲ့နေရာမှာ လူထုအခြေပြု ဘက်စုံစေတနာ့ဝန်ထမ်း (ငှက်ဖျား) လုပ်သားတွေကြုံတွေ့ရတဲ့ အတားအဆီးနဲ့စိန်ခေါ်မှုတွေကဘာတွေလဲ? အားသာချက်တွေကရောဘာ တွေလဲ? တစ်ခုချင်းရှင်းပြပါ။ 3. အဲဒီအတားအဆီးတွေကို ဘယ်လိုကျော်လွှားနိုင်မယ်လို့ သင်ထင်သလဲ?    1. သင့်အဖွဲ့အစည်းအနေနဲ့ MCBR ကဲ့သို့သောအခြားစနစ်တွေအစား ကာဗွန်လက်စ်ငှက်ဖျားလချုပ် စနစ်ကို အသုံးပြုနေရတဲ့ အကြောင်းရင်းကဘာလဲ?    2. MCBR နဲ့နှိုင်းယှဉ်ရင် ကာဗွန်လက်စ်ငှက်ဖျားလချုပ်စနစ်ရဲ့ အားသာချက်က ဘာတွေရှိလဲ? 4. For Case Detection vs. Reporting    1. ကာဗွန်လက်စ်ငှက်ဖျားလချုပ်စနစ်ရဲ့ သတင်းအချက်အလက် အရည်အသွေးကို သင့်အနေနဲ့ ကျေနပ် အားရမှုရှိပါသလား? ဘာကြောင့်လဲ? |
| **(၅) MCBR စနစ်အပေါ်အမြင်နှင့်ယူဆချက်များ** |
| - 1. MCBR စနစ် အကြောင်း ပြောပြပေးပါ။   2. MCBR ဟာ မြန်မာနိုင်ငံရဲ့ ငှက်ဖျားကင်းဝေးရေး လုပ်ငန်းမှာ ထိရောက်တဲ့ ငှက်ဖျားစောင့်ကြပ်ကြည့်ရှုရေး နည်းလမ်း တစ်ခုဖြစ်တယ်လို့ သင့်အနေနဲ့ ထင်မြင်ယူဆပါသလား? ဘာကြောင့်လဲ?   3. MCBR နဲ့ကောက်ယူထားတဲ့အချက်အလက်တွေ ကို သင် ဘယ်လိုနည်းနဲ့ ရယူသိရှိနိုင်သလဲ?   4. MCBR နဲ့ ကောက်ယူထားတဲ့ အချက်အလက်တွေကို သင် ဘယ်လိုခွဲခြမ်းလေ့လာသုံးသပ်သလဲ?   5. MCBR စနစ်ကို အသုံးပြုတဲ့နေရာမှာ သင် ရင်ဆိုင်ရတဲ့ အတားအဆီးနဲ့ စိန်ခေါ်မှုတွေက ဘာတွေလဲ?  1. အဲဒီအတားအဆီးတွေကို သင် ဘယ်လိုကျော်လွှားနိုင်မယ်လို့ သင်ထင်သလဲ?    1. MCBR စနစ်ကို အကောင်အထည်ဖော်ဆောင်၊ စီမံခန့်ခွဲတဲ့နေရာမှာ သင် ရင်ဆိုင်ရတဲ့ အတားအဆီးနဲ့ စိန်ခေါ်မှုတွေက ဘာတွေလဲ? 2. အဲဒီအတားအဆီးတွေကို သင် ဘယ်လိုကျော်လွှားနိုင်မယ်လို့ သင်ထင်သလဲ?    1. MCBR သုံးတဲ့နေရာမှာ လူထုအခြေပြု ဘက်စုံစေတနာ့ဝန်ထမ်း (ငှက်ဖျား)လုပ်သားတွေကြုံတွေ့ရတဲ့ အတားအဆီးနဲ့ စိန်ခေါ်မှုတွေက ဘာတွေလဲ? အားသာချက်တွေကရော ဘာတွေလဲ? တစ်ခုချင်းရှင်းပြပါ။ 3. အဲဒီအတားအဆီးတွေကို ဘယ်လိုကျော်လွှားနိုင်မယ်လို့ သင်ထင်သလဲ?    1. သင့်အဖွဲ့အစည်းအနေနဲ့ ကာဗွန်လက်စ်ငှက်ဖျားလချုပ်ကဲ့သို့သော အခြားစနစ်တွေအစား MCBR စနစ်ကို အသုံးပြုနေရတဲ့ အကြောင်းရင်းကဘာလဲ?    2. ကာဗွန်လက်စ်ငှက်ဖျားလချုပ်စနစ်နဲ့နှိုင်းယှဉ်ရင် MCBR ရဲ့ အားသာချက်က ဘာတွေရှိလဲ? 4. For Case Detection vs. Reporting    1. MCBR စနစ်ရဲ့ အသုံးအဝင်ဆုံးအပိုင်းက ဘာလဲ? ဘာကြောင့်လဲ?    2. ပေးပို့ထားတဲ့ သတင်းအချက်အလက်တွေကို သင် ရယူရာမှာ MCBR က ဘယ်အတိုင်းအတာအထိ တိုးတက်ကောင်းမွန်စေလဲ?    3. MCBR စနစ်ရဲ့ သတင်းအချက်အလက်အရည်အသွေးကို သင့်အနေနဲ့ ကျေနပ်အားရမှု ရှိပါသလား? ဘာကြောင့်လဲ? |
| **(၆) MCBR အပလီကေးရှင်း၏အသုံးဝင်ပုံ** |
| - 1. MCBR စနစ်ကို တိုးတက်ကောင်းမွန်အောင် ဘာတွေဆောင်ရွက် သင့်တယ်လို့ သင်ထင်သလဲ?   2. MCBR (သို့မဟုတ်) DHIS2 တို့မှရရှိသည့် စာရင်းဇယားအချက်အလက်များကို ငွေကြေးရယူသုံးစွဲမှုနှင့် အရင်းမြစ်များစီမံခန့်ခွဲမှုဆုံးဖြတ်ချက်များ ချမှတ်ရာတွင် ဘယ်လိုအသုံးပြုနေသလဲ?   3. MCBR/DHIS2 စနစ်မှ အချက်အလက်များကို ထုတ်ယူအသုံးပြုရာမှာ အကြီးမားဆုံးစိန်ခေါ်မှုက ဘာလဲ? ဘာကြောင့်လဲ?   4. သင့်အလုပ်တွေကို ပိုမိုကူညီနိုင်ဖို့ MCBR အပလီကေးရှင်းမှာ ဘာစွမ်းဆောင်ချက်တွေ ထပ်ထည့်ပေးလို့ ရမလဲ?   5. MCBR အသုံးပြုခြင်း၊ လုပ်ငန်းတိုးချဲ့ခြင်းတို့နဲ့ပတ်သက်ပြီး သင့်မှာ စိုးရိမ်နေတာမျိုးရှိသလား? အသေး စိတ်ဖော်ပြပေးပါ။ |
| **(၇) MCBR ကို အသုံးပြုရန်အတွက် လိုအပ်သည့်အထောက်အပံ့များ** |
| - 1. MCBR ကို ထိရောက်မှုရှိသော သတင်းပို့စနစ်တစ်ခုအဖြစ် ပုံမှန်အသုံးပြုနိုင်ရန် လူထုအခြေပြု ဘက်စုံစေတနာ့ဝန်ထမ်း (ငှက်ဖျား) လုပ်သားတွေကို ဘာအထောက်အပံ့တွေပေးဖို့လိုမလဲ?   (နည်းပညာနှင့်သင်တန်းများ၊ ငွေကြေး၊ နည်းပညာ၊ မူဝါဒပိုင်းဆိုင်ရာ၊ အသိပေးတင်ပြခြင်း၊ အုပ်ချုပ်ရေး ပိုင်းဆိုင်ရာ၊ စိတ်ဓာတ်ပိုင်းဆိုင်ရာ အထောက်အပံ့များ၊ အစရှိသဖြင့်)   1. ၎င်းအထောက်အပံ့များ ပြည့်စုံနေပြီလား? 2. မပြည့်စုံသေးဘူးဆိုရင် အဆိုပါလိုအပ်ချက်များကိုဖြည့်ဆည်းရန် အစီအစဉ်ချမှတ်ထားတာများ သင် သိသလား? 3. အဆိုပါလိုအပ်ချက်တွေကို အချိန်မီ ဘယ်လိုဖြည့်ဆည်းနိုင်မလဲ?    1. MCBR မှသတင်းအချက်အလက်များကို စုစည်း ထုတ်နှုတ် ဆန်းစစ်နေရသော data assistant များ၊ M&E ဝန်ထမ်းများနှင့် အခြား ဆက်စပ်ဝန်ထမ်းတွေကို ဘာအထောက်အပံ့တွေပေးဖို့လိုမလဲ?   (နည်းပညာနှင့်သင်တန်းများ၊ ငွေကြေး၊ နည်းပညာ၊ မူဝါဒပိုင်းဆိုင်ရာ၊ အသိပေးတင်ပြခြင်း၊ အုပ်ချုပ်ရေး ပိုင်းဆိုင်ရာ၊ စိတ်ဓာတ်ပိုင်းဆိုင်ရာ အထောက်အပံ့များ၊ အစရှိသဖြင့်)   1. ၎င်းအထောက်အပံ့များ ပြည့်စုံနေပြီလား? 2. မပြည့်စုံသေးဘူးဆိုရင် အဆိုပါလိုအပ်ချက်များကိုဖြည့်ဆည်းရန် အစီအစဉ်ချမှတ်ထားတာများ သင် သိသလား? 3. အဆိုပါလိုအပ်ချက်တွေကို အချိန်မီ ဘယ်လိုဖြည့်ဆည်းနိုင်မလဲ?    1. MCBR မှ သတင်းအချက်အလက်များကို အသုံးချ၍ မူဝါဒများ၊ ဆုံးဖြတ်ချက်များ ချမှတ်နေရသော အရာရှိတွေကို ဘာအထောက်အပံ့တွေပေးဖို့လိုမလဲ?   (နည်းပညာနှင့်သင်တန်းများ၊ ငွေကြေး၊ နည်းပညာ၊ မူဝါဒပိုင်းဆိုင်ရာ၊ အသိပေးတင်ပြခြင်း၊ အုပ်ချုပ်ရေး ပိုင်းဆိုင်ရာ၊ စိတ်ဓာတ်ပိုင်းဆိုင်ရာ အထောက်အပံ့များ၊ အစရှိသဖြင့်)   1. ၎င်းအထောက်အပံ့များ ပြည့်စုံနေပြီလား? 2. မပြည့်စုံသေးဘူးဆိုရင် အဆိုပါလိုအပ်ချက်များကိုဖြည့်ဆည်းရန် အစီအစဉ်ချမှတ်ထားတာများ သင် သိသလား? 3. အဆိုပါလိုအပ်ချက်များကို အချိန်မီ ဘယ်လိုဖြည့်ဆည်းနိုင်မလဲ? |
| **(၈) MCBR အပလီကေးရှင်း၏ ရေရှည်တည်တံ့မှု** |
| 8.1. လိုအပ်တဲ့ပြုပြင်မှုတွေပြုလုပ်မယ်၊ လိုအပ်တဲ့အထောက်အပံ့တွေလည်း ပေးမယ်ဆိုရင် MCBR အပလီကေးရှင်းကို လူထုအခြေပြု ဘက်စုံစေတနာ့ဝန်ထမ်း (ငှက်ဖျား)လုပ်သားတွေအသုံးပြုတာဟာ ရေရှည်တည်တံ့မယ်လို့ သင်ထင်ပါသလား? ဘာကြောင့်လဲ?  8.2. MCBR အပလီကေးရှင်းကို ငှက်ဖျားသတင်းအချက်အလက်များပေးပို့ရာမှာ ရေရှည်တည်တံ့ဖို့ အဓိကကျတဲ့ အချက်တွေကဘာတွေလဲ? MCBR အပလီကေးရှင်းကိုရေရှည်တည်တံ့ဖို့ အဲဒီအချက်တွေကို ဘယ်လိုဦးတည်စဉ်းစားမလဲ?  8..3. MCBR အပလီကေးရှင်း ရေရှည်တည်တံ့ဖို့ ဘယ်သူတွေမှာတာဝန်ရှိလဲ? အမျိုးသားငှက်ဖျားရောဂါ တိုက်ဖျက်ရေးစီမံချက်အနေနဲ့ အနာဂတ်မှာ MCBR အပလီကေးရှင်းကိုလက်လွှဲရယူပြီး စီမံခန့်ခွဲဖို့ ဖြစ်နိုင်မယ်လို့ ထင်ပါသလား? ဘာကြောင့်လဲ? ကျေးဇူးပြု၍ ဆွေးနွေးပေးပါ။  8.4. အပလီကေးရှင်းအသုံးပြုပြီးသတင်းပို့တာကို လူထုအခြေပြု ဘက်စုံစေတနာ့ဝန်ထမ်း (ငှက်ဖျား)လုပ်သားအစီအစဉ်မှာပါတဲ့ ငှက်ဖျားမဟုတ်တဲ့တခြားရောဂါတွေအတွက်ရော အသုံးပြုနိုင်မလား?  8.5. အပလီကေးရှင်းအသုံးပြုပြီး သတင်းပို့တာကို ဘယ်သူတွေအသုံးပြုဖို့လိုမလဲ (ကျန်းမာရေးဝန်ထမ်း၊ သားဖွားဆရာမ၊ စသည်ဖြင့်)? ကျေးဇူးပြု၍ ဆွေးနွေးပေးပါ။  8.6. အပလီကေးရှင်းအသုံးပြုပြီး သတင်းပို့နိုင်ဖို့ အပလီကေးရှင်းနဲ့ စနစ်ပိုင်းဆိုင်ရာမှာ ဘာပြုပြင်ပြောင်းလဲ မှုတွေလုပ်ဖို့လိုမလဲ? ကျေးဇူးပြု၍ ဆွေးနွေးပေးပါ။  8.7. အပလီကေးရှင်းအသုံးပြုပြီး သတင်းပို့နိုင်ဖို့ ဘယ်လို အရင်းအမြစ်တွေလိုအပ်မလဲ? ကျေးဇူးပြု၍ ဆွေးနွေးပေးပါ။ |
| **(၉)ခြုံငုံသုံးသပ်ခြင်း** |
| - 1. မြန်မာနိုင်ငံငှက်ဖျားကင်းဝေးရေးလုပ်ငန်းအတွက် ငှက်ဖျားသတင်း အချက်အလက်များ ပေးပို့ရာမှာ MCBR က ကာဗွန်လက်စ် ငှက်ဖျားလချုပ်ကို လုံးဝ အစားထိုးနိုင်မယ်လို့ သင်ထင်ပါသလား? ဘာကြောင့်လဲ?   2. အစားထိုးနိုင်မယ်ဆိုရင် ကာဗွန်လက်စ်ငှက်ဖျားလချုပ်စနစ်ကနေ MCBR စနစ်ကိုကူးပြောင်းရာမှာ အကောင်းဆုံးဖြစ်ဖို့ဘာတွေလုပ်ဖို့လိုမလဲ? အဲဒီလိုကူးပြောင်းရာမှာသတိထားရမယ့်အချက်တွေက ဘာ တွေရှိလဲ?   3. ငှက်ဖျားရောဂါဆိုင်ရာသတင်းအချက်အလက်များပေးပို့ခြင်းနဲ့ပတ်သက်ပြီး ဖြည့်စွက်အကြံပြုဆွေးနွေးလို တာများ ရှိသေးသလား? |

မေးခွန်းများမေးမြန်းခြင်းပြီးပါပြီ။ ဤသုတေသနနှင့်ပတ်သက်၍ မေးမြန်းလိုသည်များရှိပါသလား?

အခြားမေးမြန်းလိုသည်များ ရှိပါသလား?

အချိန်ပေး၍ ပါဝင်ဖြေဆိုပေးသည့်အတွက် ကျေးဇူးအထူးတင်ရှိပါသည်။

**Appendix G: Key Informant Interview Guide: Ministry of Health and Sport**

**Is it ok to audio record this conversation?** a. Yes b. No

| **Background Information** | | | |  |
| --- | --- | --- | --- | --- |
| 0.1 | Name of interviewer: | ____________________ | |  |
| 0.2 | Date (DD/MM/YYYY): | ________ | |  |
| 0.3 | Start time: | ________ | |  |
| 0.4 | End time: | ________ | |  |
| 0.5 | Job level (indicate): | Higher level / Middle level  National/State/Regional policy maker | |  |
| 0.6 | Organization (optional) | ____________________ | |  |
| 0.7 | How long have you worked in your current position (in terms of year)? | ________ | |  |
| 0.8 | How many years of work experience in malaria sector do you have (in total)? | ________ | |  |
| **Section 1: Roles and responsibilities** | | | |  |
|  | Describe your current role and key responsibilities | |  |  |
| **Section 2: Current malaria situation** | | | |  |
|  | What’s the situation of malaria in your assigned territory? | |  |  |
|  | Why do you think that the burden of malaria in your area is going up/down or plateauing? | |  |  |
| **Section 3: Views and perspectives on paper-based reporting (PBR)** | | | |  |
|  | Can you describe the PBR system for malaria case reporting? | |  |  |
|  | How do you receive and access the data collected by PBR? Please give examples. | |  |  |
|  | How do you analyse the data collected by PBR? | |  |  |
|  | Do you think PBR is an effective method of malaria surveillance in an elimination setting like Myanmar? Why? If not, why? | |  |  |
|  | What are barriers / challenges in managing the PBR systems? | |  |  |
|  | What are the advantages to using in PBR systems? | |  |  |
|  | Are you happy with the data quality of the PBR systems?  Why/ Why not? | |  |  |
| **Section 4: Views and perspectives on MCBR** | | | |  |
|  | Can you describe the MCBR system for malaria case reporting? | |  |  |
|  | How do you receive and access the data collected by MCBR? | |  |  |
|  | How do you analyse the data collected by MCBR? Please give examples. | |  |  |
|  | Do you think MCBR is an effective method of malaria surveillance in an elimination setting like Myanmar? Why? If not, why? | |  |  |
|  | What are barriers / challenges in managing the MCBR? | |  |  |
|  | What are the advantages to using in MCBR system? | |  |  |
|  | To what extent does the MCBR improve your access to reporting data? | |  |  |
|  | Are you happy with the data quality of the MCBR systems?  Why/ Why not? | |  |  |
|  | What impact do you perceive that using the MCBR will have on malaria elimination in Myanmar? | |  |  |
| **Section 5: Usability of MCBR** | | | |  |
|  | How do you think the MCBR system could be improved? | |  |  |
|  | How do you currently use the data from MCBR /DHIS2 to inform decision making around funding/resources allocation? | |  |  |
|  | What is the most challenging part of accessing and/or using data gathered through MCBR/ DHIS2? Why? | |  |  |
|  | What concerns do you have in using/scaling up MCBR? Please elaborate | |  |  |
|  | How do you ensure that the data collected by ICMVs is complete and accurate? | |  |  |
|  | How is the data collected by MCBR monitored and how are failures reported and addressed? | |  |  |
| **Section 6: Barriers and enablers to applying MCBR** | | | |  |
|  | What are the barriers and enablers of implementing and managing the MCBR as a reporting system in the malaria program? | |  |  |
|  | Barriers  How can we mitigate/solve those barriers? | |  |  |
|  | Enablers | |  |  |
| **Section 7: Funding and cost-effectiveness for MCBR** | | | |  |
|  | Is MCBR more cost effective than PBR? Why? Why not? | |  |  |
|  | How is MCBR funded currently? | |  |  |
|  | If MCBR is to be scaled-up and rolled-out nationally, how will it be funded? | |  |  |
|  | Do you foresee any potential problems for MCBR funding? | |  |  |
| **Section 8: Supports needed for applying MCBR in malaria program** | | | |  |
|  | Which supports are needed for ICMV to expand the MCBR as a routine and effective reporting channel?  (technical and training, financial, technological, political, advocacy, administrative, moral etc)  Are they been fulfilled?  If not, do you see any plan to fulfil those requirements?  If not, how can we best fulfil those requirements in time? | |  |  |
|  | Which supports are needed for data assistants, M&E staff and/or any data staff who have responsibility to compile, enter and analyse data from MCBR? (technical and training, financial, technological, political, advocacy, administrative, moral etc)  Are they been fulfilled?  If not, do you see any plan to fulfil those requirements?  If not, how can we best fulfil those requirements in time? | |  |  |
|  | Which supports are needed for policy makers and/or decision makers to access and utilize data from MCBR? (technical and training, financial, technological, political, advocacy, administrative, moral etc)  Are they been fulfilled?  If not, do you see any plan to fulfil those requirements?  If not, how can we best fulfil those requirements in time? | |  |  |
| **Section 9: Sustainability in the context of ICMV** | | | |  |
|  | Do you think MCBR application is sustainable in the context of ICMV if necessary improvements are made and/or necessary supports are provided? Why? Why not? | |  |  |
|  | What are the key factors that will determine sustainability of MCBR for malaria reporting and surveillance in the malaria program? How do we address them to sustain the MCBR in long run? | |  |  |
|  | Who is responsible for sustaining the MCBR? Do you think NMCP can maintain the operation of MCBR without support from Save the Children? Why? Please discuss in detail including the following discussion points:  Server maintenance, server expansion, safeguarding data | |  |  |
|  | What kinds of adaptations need to be made to the application and overall system to enable NMCP to take maintain operation of MCBR? | |  |  |
|  | What kinds of resources are needed to enable NMCP to maintain operation of MCBR? | |  |  |
|  | Can we use app-based reporting channel for reporting of non-malaria ICMV diseases? | |  |  |
|  | What kinds of adaptations need to be made to the application and overall system to enable reporting of non-malaria disesase covered by ICMV? | |  |  |
|  | What kind of resources are needed to enable reporting of non-malaria disesase covered by ICMV? | |  |  |
|  | Who will be using the app-based reporting channel (BHS, Midwife, etc.)? Please discuss. | |  |  |
|  | What kinds of adaptations need to be made to the application and overall system to enable non-ICMV personnel (e.g. BHS, midwives) to use the application? | |  |  |
|  | What kind of resources are needed to enable non-ICMV personnel (e.g. BHS, midwives) to use the application? | |  |  |
| **Section 10: Overall** | | | |  |
|  | Do you think PBR can be totally replaced by MCBR for reporting of malaria cases in Myanmar malaria elimination program? Why /Why not?  If yes, how can we best transition from PBR to MCBR? What are the factors that need to be taken care off in the transition? | |  |  |
|  | Do you have any other suggestions/ comments regarding the reporting of malaria cases? If yes, please make comments. | |  |  |

Do you have any questions for me?

Thank you very much for your participation.

**End of session**

ငှက်ဖျားရောဂါဆိုင်ရာသတင်းအချက်အလက်များပေးပို့ရာတွင် မိုဘိုင်းအပလီကေးရှင်းသုံးခြင်းနှင့် ကာဗွန်လက်စ်ငှက်ဖျားလချုပ်သုံးခြင်းတို့ကို နှိုင်းယှဉ်လေ့လာခြင်း

(ကျန်းမာရေးနှင့်အားကစားဝန်ကြီးဌာနမှအရာရှိများအားမေးမြန်းမည့်လမ်းညွှန်)

ယခုအင်တာဗျူးကို အသံသွင်းထားရန် ခွင့်ပြုပါသလား? (1) ပြုပါသည်။ (2) မပြုပါ။

| **အခြေခံအချက်အလက်များ** | | |
| --- | --- | --- |
| 0.1 | တွေ့ဆုံမေးမြန်းသူ၏အမည် |  |
| 0.2 | ရက်စွဲ (ရက်/လ/နှစ်) |  |
| 0.3 | စတင်ချိန် |  |
| 0.4 | ပြီးဆုံးချိန် |  |
| 0.5 | ရာထူးအဆင့် (ဖော်ြပပါ): | အကြီးတန်း/အလယ်အလတ်တန်း  ဗဟို/တိုင်းဒေသကြီး (သို့မဟုတ်) ပြည်နယ်အဆင့် မူဝါဒရေးဆွဲသူ |
| 0.6 | အဖွဲ့အစည်းအမည် (မဖြေလိုကချန်ထားနိုင်သည်။) |  |
| 0.7 | သင် ယခုရာထူးမှာတာဝန်ထမ်းဆောင်နေတာ ဘယ်လောက်ကြာပြီလဲ? | ..................နှစ် |
| 0.8 | သင့်မှာ ငှက်ဖျားနဲ့ပတ်သက်ပြီး လုပ်ငန်းအတွေ့အကြုံစုစုပေါင်း ဘယ်လောက်ရှိသလဲ? | ..................နှစ် |

| **(၁) အခန်းကဏ္ဍနှင့်တာဝန်ယူမှု** |
| --- |
| - 1. သင့်ရဲ့ လက်ရှိ အခန်းကဏ္ဍနဲ့ တာဝန်ယူရတဲ့အပိုင်းတွေကို ပြောပြပေးပါ။ |
| **(၂) ယခုလက်ရှိ ငှက်ဖျားအခြေအနေ** |
| - 1. သင် တာဝန်ကျတဲ့ဒေသရဲ့ ငှက်ဖျားအခြေအနေကို ပြောပြပေးပါ။   2. ဘာကြောင့် သင်တာဝန်ကျတဲ့ဒေသမှာငှက်ဖျားရောဂါအခြေအနေကောင်းမွန်လာ/ဆိုးရွားလာ/ဒီအတိုင်းပဲ တန့်နေတယ်လို့ ထင်သလဲ? |
| **(၃) ကာဗွန်လက်စ်ငှက်ဖျားလချုပ်စနစ်အပေါ်အမြင်နှင့်ယူဆချက်များ** |
| - 1. ကာဗွန်လက်စ်ငှက်ဖျားလချုပ်စနစ် အကြောင်း ပြောပြပေးပါ။ (သမိုင်းကြောင်း၊ အသုံးဝင်မှု စသည်ဖြင့်)   2. ကာဗွန်လက်စ်ငှက်ဖျားလချုပ်နဲ့ကောက်ယူထားတဲ့အချက်အလက်တွေ ကို သင် ဘယ်လိုနည်းနဲ့ ရယူသိရှိ နိုင်သလဲ? ဥပမာပေးပေးပါ။   3. ကာဗွန်လက်စ်ငှက်ဖျားလချုပ်နဲ့ကောက်ယူထားတဲ့အချက်အလက်တွေကို သင် ဘယ်လိုခွဲခြမ်းလေ့လာ သုံးသပ်သလဲ?   4. ကာဗွန်လက်စ်ငှက်ဖျားလချုပ်ဟာ မြန်မာနိုင်ငံရဲ့ ငှက်ဖျားကင်းဝေးရေး လုပ်ငန်းမှာ ထိရောက်တဲ့ ငှက်ဖျားစောင့်ကြပ်ကြည့်ရှုရေးနည်းလမ်း တစ်ခုဖြစ်တယ်လို့ သင့်အနေနဲ့ ထင်မြင်ယူဆပါသလား? ဘာကြောင့်လဲ?   5. ကာဗွန်လက်စ်ငှက်ဖျားလချုပ် စနစ်ကို စီမံတဲ့နေရာမှာ ရင်ဆိုင်ရတဲ့ အတားအဆီးနဲ့ စိန်ခေါ်မှုတွေက ဘာတွေလဲ?   6. ကာဗွန်လက်စ်ငှက်ဖျားလချုပ်စနစ်ရဲ့ အားသာချက်တွေက ဘာတွေလဲ?   7. ကာဗွန်လက်စ်ငှက်ဖျားလချုပ်စနစ်ရဲ့ သတင်းအချက်အလက်အရည်အသွေးကို သင့်အနေနဲ့ ကျေနပ် အားရမှုရှိပါသလား? ဘာကြောင့်လဲ? |
| **(၄) MCBR စနစ်အပေါ်အမြင်နှင့် ယူဆချက်များ** |
| - 1. MCBR စနစ် အကြောင်း ပြောပြပေးပါ။ (သမိုင်းကြောင်း၊ အသုံးဝင်မှု စသည်ဖြင့်)   2. MCBR နဲ့ကောက်ယူထားတဲ့အချက်အလက်တွေ ကို သင် ဘယ်လိုနည်းနဲ့ ရယူသိရှိနိုင်သလဲ?   3. MCBR နဲ့ ကောက်ယူထားတဲ့ အချက်အလက်တွေကို သင် ဘယ်လိုခွဲခြမ်းလေ့လာသုံးသပ်သလဲ? ဥပမာ ပေး ပေးပါ။   4. MCBR ဟာ မြန်မာနိုင်ငံရဲ့ ငှက်ဖျားကင်းဝေးရေး လုပ်ငန်းမှာ ထိရောက်တဲ့ ငှက်ဖျားစောင့်ကြပ်ကြည့်ရှုရေး နည်းလမ်း တစ်ခုဖြစ်တယ်လို့ သင့်အနေနဲ့ ထင်မြင်ယူဆပါသလား? ဘာကြောင့်လဲ?   5. MCBR စနစ်ကို စီမံတဲ့နေရာမှာ ရင်ဆိုင်ရတဲ့ အတားအဆီးနဲ့ စိန်ခေါ်မှုတွေက ဘာတွေလဲ?   6. MCBR စနစ်ရဲ့ အားသာချက်တွေက ဘာတွေလဲ?   7. ပေးပို့ထားတဲ့အချက်အလက်တွေကို သင် ရယူရာမှာ MCBR က ဘယ်အတိုင်းအတာအထိ တိုးတက် ကောင်းမွန်စေလဲ?   8. MCBR စနစ်ရဲ့ သတင်းအချက်အလက်အရည်အသွေးကို သင့်အနေနဲ့ ကျေနပ်အားရမှု ရှိပါသလား? ဘာကြောင့်လဲ?   9. MCBR အသုံးပြုခြင်းက မြန်မာနိုင်ငံရဲ့ ငှက်ဖျားကင်းဝေးရေးလုပ်ငန်း ဆောင်ရွက်ရာမှာ ဘယ်လောက် အထိ အကျိုးသက်ရောက်မှုရှိစေမယ် လို့ သင်ယုံကြည်သလဲ? |
| **(၅) MCBR အပလီကေးရှင်း၏အသုံးဝင်ပုံ** |
| - 1. MCBR စနစ်ကို တိုးတက်ကောင်းမွန်အောင် ဘာတွေဆောင်ရွက် သင့်တယ်လို့ သင်ထင်သလဲ?   2. MCBR (သို့မဟုတ်) DHIS2 တို့မှရရှိသည့် စာရင်းဇယားအချက်အလက်များကို ငွေကြေးရယူသုံးစွဲမှုနှင့် အရင်းမြစ်များစီမံခန့်ခွဲမှုဆုံးဖြတ်ချက်များ ချမှတ်ရာတွင် ဘယ်လိုအသုံးပြုနေသလဲ?   3. MCBR/DHIS2 စနစ်မှ အချက်အလက်များကို ထုတ်ယူအသုံးပြုရာမှာ အကြီးမားဆုံးစိန်ခေါ်မှုက ဘာလဲ? ဘာကြောင့်လဲ?   4. MCBR အသုံးပြုခြင်း၊ လုပ်ငန်းတိုးချဲ့ခြင်းတို့နဲ့ပတ်သက်ပြီး သင့်မှာ စိုးရိမ်နေတာမျိုးရှိသလား? အသေး စိတ်ဖော်ပြပေးပါ။   5. လူထုအခြေပြု ဘက်စုံစေတနာ့ဝန်ထမ်း (ငှက်ဖျား)လုပ်သားတစ်ယောက်ကောက်ယူထားတဲ့ အချက် အလက်တွေက ပြည့်စုံတိကျမှုရှိတယ်ဆိုတာကို သင့်အနေနဲ့ ဘယ်လိုသေချာစေမလဲ?   6. MCBR သုံးပြီးကောက်ထားတဲ့ အချက်အလက်တွေကို ဘယ်လို စောင့်ကြည့်လဲ? ချို့ယွင်းချက်တွေတွေ့ရင် ဘယ်လို သတင်းပို့သလဲ? ဘယ်လိုအရေးယူဆောင်ရွက်သလဲ? |
| **(၆) MCBR အသုံးပြုခြင်း၏အားသာချက်နှင့် အခက်အခဲများ** |
| - 1. ငှက်ဖျားရောဂါတိုက်ဖျက်ရေးစီမံကိန်းတွင် MCBR အား စာရင်းဇယားများအစီရင်ခံတင်ပြခြင်းစနစ်တစ်ခု အဖြစ် အကောင်အထည်ဖော်စီမံခန့်ခွဲရာ၌ ကြုံတွေ့ရသည့်အားသာချက်နှင့် အခက်အခဲများကို ဖော်ပြပါ။   2. အခက်အခဲများကို မည်ကဲ့သို့ လျော့နည်းပပျောက်အောင် ဆောင်ရွက်မည်နည်း?   3. အားသာချက်များ |
| **(၇) MCBR အတွက် ငွေကြေးအထောက်အပံ့နှင့် ကုန်ကျစားရိတ်နှင့်ထိရောက်ကာမိမှု (cost-effectiveness)** |
| - 1. MCBR အသုံးပြုခြင်းက ကာဗွန်လက်စ်ငှက်ဖျားလချုပ်အသုံးပြုခြင်းနှင့်နှိုင်းယှဉ်လျှင် ပိုပြီး ကုန်ကျစားရိတ် နှင့် ထိရောက်ကာမိမှု (cost-effectiveness) ရှိပါသလား?   2. အခုလက်ရှိ MCBR အတွက်ငွေကြေးအထောက်အပံ့ဘယ်လိုရရှိနေလဲ?   3. MCBR ကို တစ်နိုင်ငံလုံးအတိုင်းအတာနဲ့ တိုးချဲ့အသုံးပြုမယ်ဆိုရင် ဘယ်လို ငွေကြေးအထောက်အပံ့ လိုမလဲ?   4. MCBR အတွက် ငွေကြေးအထောက်အပံ့နဲ့ပတ်သက်ပြီး ဖြစ်လာနိုင်ချေရှိတဲ့ ပြဿနာတစ်စုံတစ်ရာကိုများ ကြိုတင်မှန်းဆမိတာရှိသလား? |
| **(၈) MCBR ကို အသုံးပြုရန်အတွက် လိုအပ်သည့်အထောက်အပံ့များ** |
| - 1. MCBR ကို ထိရောက်မှုရှိသော သတင်းပို့စနစ်တစ်ခုအဖြစ် ပုံမှန်အသုံးပြုနိုင်ရန် လူထုအခြေပြု ဘက်စုံစေတနာ့ဝန်ထမ်း (ငှက်ဖျား) လုပ်သားတွေကို ဘာအထောက်အပံ့တွေပေးဖို့လိုမလဲ?   (နည်းပညာနှင့်သင်တန်းများ၊ ငွေကြေး၊ နည်းပညာ၊ မူဝါဒပိုင်းဆိုင်ရာ၊ အသိပေးတင်ပြခြင်း၊ အုပ်ချုပ်ရေး ပိုင်းဆိုင်ရာ၊ စိတ်ဓာတ်ပိုင်းဆိုင်ရာ အထောက်အပံ့များ၊ အစရှိသဖြင့်)   1. ၎င်းအထောက်အပံ့များ ပြည့်စုံနေပြီလား? 2. မပြည့်စုံသေးဘူးဆိုရင် အဆိုပါလိုအပ်ချက်များကိုဖြည့်ဆည်းရန် အစီအစဉ်ချမှတ်ထားတာများ သင် သိသလား? 3. အဆိုပါလိုအပ်ချက်တွေကို အချိန်မီ ဘယ်လိုဖြည့်ဆည်းနိုင်မလဲ?    1. MCBR မှသတင်းအချက်အလက်များကို စုစည်း ထုတ်နှုတ် ဆန်းစစ်နေရသော data assistant များ၊ M&E ဝန်ထမ်းများနှင့် အခြား ဆက်စပ်ဝန်ထမ်းတွေကို ဘာအထောက်အပံ့တွေပေးဖို့လိုမလဲ?   (နည်းပညာနှင့်သင်တန်းများ၊ ငွေကြေး၊ နည်းပညာ၊ မူဝါဒပိုင်းဆိုင်ရာ၊ အသိပေးတင်ပြခြင်း၊ အုပ်ချုပ်ရေး ပိုင်းဆိုင်ရာ၊ စိတ်ဓာတ်ပိုင်းဆိုင်ရာ အထောက်အပံ့များ၊ အစရှိသဖြင့်)   1. ၎င်းအထောက်အပံ့များ ပြည့်စုံနေပြီလား? 2. မပြည့်စုံသေးဘူးဆိုရင် အဆိုပါလိုအပ်ချက်များကိုဖြည့်ဆည်းရန် အစီအစဉ်ချမှတ်ထားတာများ သင် သိသလား? 3. အဆိုပါလိုအပ်ချက်တွေကို အချိန်မီ ဘယ်လိုဖြည့်ဆည်းနိုင်မလဲ?    1. MCBR မှ သတင်းအချက်အလက်များကို အသုံးချ၍ မူဝါဒများ၊ ဆုံးဖြတ်ချက်များ ချမှတ်နေရသော အရာရှိတွေကို ဘာအထောက်အပံ့တွေပေးဖို့လိုမလဲ?   (နည်းပညာနှင့်သင်တန်းများ၊ ငွေကြေး၊ နည်းပညာ၊ မူဝါဒပိုင်းဆိုင်ရာ၊ အသိပေးတင်ပြခြင်း၊ အုပ်ချုပ်ရေး ပိုင်းဆိုင်ရာ၊ စိတ်ဓာတ်ပိုင်းဆိုင်ရာ အထောက်အပံ့များ၊ အစရှိသဖြင့်)   1. ၎င်းအထောက်အပံ့များ ပြည့်စုံနေပြီလား? 2. မပြည့်စုံသေးဘူးဆိုရင် အဆိုပါလိုအပ်ချက်များကိုဖြည့်ဆည်းရန် အစီအစဉ်ချမှတ်ထားတာများ သင် သိသလား? 3. အဆိုပါလိုအပ်ချက်များကို အချိန်မီ ဘယ်လိုဖြည့်ဆည်းနိုင်မလဲ? |
| **(၉) MCBR အပလီကေးရှင်း၏ ရေရှည်တည်တံ့မှု** |
| 9.1. လိုအပ်တဲ့ပြုပြင်မှုတွေပြုလုပ်မယ်၊ လိုအပ်တဲ့အထောက်အပံ့တွေလည်း ပေးမယ်ဆိုရင် MCBR အပလီကေးရှင်းကို လူထုအခြေပြု ဘက်စုံစေတနာ့ဝန်ထမ်း (ငှက်ဖျား)လုပ်သားတွေအသုံးပြုတာဟာ ရေရှည်တည်တံ့မယ်လို့ သင်ထင်ပါသလား? ဘာကြောင့်လဲ?  9.2. MCBR အပလီကေးရှင်းကို ငှက်ဖျားသတင်းအချက်အလက်များပေးပို့ရာမှာ ရေရှည်တည်တံ့ဖို့ အဓိကကျတဲ့ အချက်တွေကဘာတွေလဲ? MCBR အပလီကေးရှင်းကိုရေရှည်တည်တံ့ဖို့ အဲဒီအချက်တွေကို ဘယ်လိုဦးတည်စဉ်းစားမလဲ?  9.3. MCBR အပလီကေးရှင်း ရေရှည်တည်တံ့ဖို့ ဘယ်သူတွေမှာတာဝန်ရှိလဲ? အမျိုးသားငှက်ဖျားရောဂါ တိုက်ဖျက်ရေးစီမံချက်အနေနဲ့ အနာဂတ်မှာ MCBR အပလီကေးရှင်းကိုလက်လွှဲရယူပြီး စီမံခန့်ခွဲဖို့ ဖြစ်နိုင်မယ်လို့ ထင်ပါသလား? ဘာကြောင့်လဲ? ကျေးဇူးပြု၍ ဆွေးနွေးပေးပါ။  9.4. အပလီကေးရှင်းအသုံးပြုပြီးသတင်းပို့တာကို လူထုအခြေပြု ဘက်စုံစေတနာ့ဝန်ထမ်း (ငှက်ဖျား)လုပ်သားအစီအစဉ်မှာပါတဲ့ ငှက်ဖျားမဟုတ်တဲ့တခြားရောဂါတွေအတွက်ရော အသုံးပြုနိုင်မလား?  9.5. အပလီကေးရှင်းအသုံးပြုပြီး သတင်းပို့တာကို ဘယ်သူတွေအသုံးပြုဖို့လိုမလဲ (ကျန်းမာရေးဝန်ထမ်း၊ သားဖွားဆရာမ၊ စသည်ဖြင့်)? ကျေးဇူးပြု၍ ဆွေးနွေးပေးပါ။  9.6. အပလီကေးရှင်းအသုံးပြုပြီး သတင်းပို့နိုင်ဖို့ အပလီကေးရှင်းနဲ့ စနစ်ပိုင်းဆိုင်ရာမှာ ဘာပြုပြင်ပြောင်းလဲ မှုတွေလုပ်ဖို့လိုမလဲ? ကျေးဇူးပြု၍ ဆွေးနွေးပေးပါ။  9.7. အပလီကေးရှင်းအသုံးပြုပြီး သတင်းပို့နိုင်ဖို့ ဘယ်လို အရင်းအမြစ်တွေလိုအပ်မလဲ? ကျေးဇူးပြု၍ ဆွေးနွေးပေးပါ။ |
| **(၁၀)ခြုံငုံသုံးသပ်ခြင်း** |
| 10.1. မြန်မာနိုင်ငံငှက်ဖျားကင်းဝေးရေးလုပ်ငန်းအတွက် ငှက်ဖျားသတင်း အချက်အလက်များ ပေးပို့ရာမှာ MCBR က ကာဗွန်လက်စ် ငှက်ဖျားလချုပ်ကို လုံးဝ အစားထိုးနိုင်မယ်လို့ သင်ထင်ပါသလား? ဘာကြောင့်လဲ?  10.2. အစားထိုးနိုင်မယ်ဆိုရင် ကာဗွန်လက်စ်ငှက်ဖျားလချုပ်စနစ်ကနေ MCBR စနစ်ကိုကူးပြောင်းရာမှာ အကောင်းဆုံးဖြစ်ဖို့ဘာတွေလုပ်ဖို့လိုမလဲ? အဲဒီလိုကူးပြောင်းရာမှာသတိထားရမယ့်အချက်တွေက ဘာ တွေရှိလဲ?  10.3. ငှက်ဖျားရောဂါဆိုင်ရာသတင်းအချက်အလက်များပေးပို့ခြင်းနဲ့ပတ်သက်ပြီး ဖြည့်စွက်အကြံပြုဆွေးနွေးလို တာများ ရှိသေးသလား? |

မေးခွန်းများမေးမြန်းခြင်းပြီးပါပြီ။ ဤသုတေသနနှင့်ပတ်သက်၍ မေးမြန်းလိုသည်များရှိပါသလား?

အခြားမေးမြန်းလိုသည်များ ရှိပါသလား?

အချိန်ပေး၍ ပါဝင်ဖြေဆိုပေးသည့်အတွက် ကျေးဇူးအထူးတင်ရှိပါသည်။

**Appendix H: Fieldwork Observation Guide: ICMVs**

| **Background Information** | | |
| --- | --- | --- |
| 0.1 | Name of observer |  |
| 0.4 | Date (DD/MM/YYYY): |  |
| 0.5 | Start time: |  |
| 0.6 | End time: |  |
| **Section 1: Responsibilities and scope of work** | | |
| 1.1 | How much time does the ICMV spend performing malaria testing and case reporting? | ______ hours/day |
| 1.2 | How many clients did the ICMV see? | ______ per hour **or** _____per day |
| 1.3 | What type of record-keeping tools does the ICMV take when testing malaria in the community? |  |
| **Section 2: Reporting of malaria cases** | | |
| 2.1 | Does the ICMV usually use PBR or MCBR to record malaria cases in the community? |  |
| 2.2 | How much time passes between when the ICMV performs a malaria test and when it is recorded on PBR |  |
| 2.3 | How much time passes between when the ICMV performs a malaria test and when it is recorded on MCBR |  |
| 2.4 | How much time passes between when the ICMV records a malaria test and when that information is transmitted to SR? |  |
| **Section 3: Workflow observations** | | |
| 3.1 | How is the ICMV contacted by the client? |  |
| 3.2 | Where does the ICMV perform the malaria test? |  |
| 3.3 | What services does the ICMV provide? |  |
| 3.4 | Does the ICMV refer any clients? If so, where do the clients go? |  |
| 3.5 | How is the workday structured? What activities are conducted throughout the course of the observation? |  |
| 3.6 | On average, how much time is spent with each client? |  |
| 3.7 | Where is the ICMV recording data associated with the malaria test?  PBR, MCBR, Other? |  |
| 3.8 | When do they enter data into the PBR, MCBR, Other? |  |
| 3.9 | What type of work does the ICMV do when they are not with a client? |  |
| 3.10 | How does the ICMV manage or resolve challenges faced during the day? |  |
| 3.11 | Are there any special circumstances that led to variations in workflow, scheduling, reporting? What are the reasons for the changes? |  |
| 3.12 | What types of activities are the most time-consuming for the ICMV? |  |
| **Section 4: facilitators and barriers in use of the MCBR** | | |
| 4.1 | Has the ICMV developed any technique to utilise the MCBR? / PBR? |  |
| 4.2 | Is the ICMV experiencing any issues with utilising the MCBR? / PBR? |  |
| 4.3 | Does the VHV have access to the internet? |  |
| 4.4 | Is the MCBR application functioning? Is it uploading the information? |  |
| **Section 5: stock management of medicine and supplies** | | |
| 5.1 | What did the ICMV do to report a stock management of medicine or supplies? |  |
| 5.2 | Did they have any issue? |  |
| **Section 6: interaction with supervisors (NMCP staff, BHSs and IP staff) regarding malaria case reporting** | | |
| 6.1 | Did the ICMV meet with supervisors? |  |
| 6.2 | What did they discuss? |  |
| 6.3 | Did any discussion relate to the MCBR? / PBR systems? |  |
| 6.4 | What kind of support related to MCBR or PBR did the supervisor provide to ICMV? |  |

**ငှက်ဖျားရောဂါဆိုင်ရာ သတင်းအချက်အလက်များပေးပို့ရာတွင် မိုဘိုင်းအပလီကေရှင်း (MCBR) သုံးခြင်းနှင့် ကာဗွန်လက်စ် ငှက်ဖျားလချုပ်များသုံးခြင်း (PBR) တို့၏ ထိရောက်မှုကို နှိုင်းယှဉ်လေ့လာခြင်း**

**လူထုအခြေပြု ဘက်စုံစေတနာ့ဝန်ထမ်း (ငှက်ဖျား)လုပ်သား (ICMV)များအား ကွင်းဆင်းလေ့လာကြည့်ရှုခြင်းလမ်းညွှန်**

| **အခြေခံအချက်အလက်များ** | | |
| --- | --- | --- |
| 0.1 | လေ့လာကြည့်ရှုသူ အမည် |  |
| 0.4 | ရက်စွဲ (ရက်/လ/နှစ်) |  |
| 0.5 | စတင်ချိန် |  |
| 0.6 | ပြီးဆုံးချိန် |  |
| **Section 1: Responsibilities and scope of work** | | |
| 1.1 | လူထုအခြေပြု ဘက်စုံစေတနာ့ဝန်ထမ်း (ငှက်ဖျား)လုပ်သားက ငှက်ဖျားသွေးဖောက်စစ်ဆေးတာနဲ့ သတင်းအချက်အလက်တွေဖြည့်သွင်း ပေးပို့ဖို့ တစ်နေ့ကို အချိန်ဘယ်လောက်သုံးသလဲ? | PBR  ______ နာရီ/ရက်  MCBR  ______ နာရီ/ရက် |
| 1.2 | လူထုအခြေပြု ဘက်စုံစေတနာ့ဝန်ထမ်း (ငှက်ဖျား)လုပ်သားက လူနာ ဘယ်နှယောက်ကြည့်ရှုသလဲ? | ______ ယောက် (တစ်ရက်လျှင်) |
| 1.3 | လူထုအခြေပြု ဘက်စုံစေတနာ့ဝန်ထမ်း (ငှက်ဖျား)လုပ်သားက ရပ်ရွာထဲမှာ ငှက်ဖျားစစ်ဆေးရာမှာ မှတ်တမ်းယူဖို့ ဘယ်လိုကိရိယာ (tools) တွေကို သုံးလဲ? |  |
| **Section 2: Reporting of malaria cases** | | |
| 2.1 | ငှက်ဖျားရောဂါဆိုင်ရာ သတင်းအချက်အလက်တွေပေးပို့ဖို့ လူထုအခြေပြု ဘက်စုံစေတနာ့ဝန်ထမ်း (ငှက်ဖျား)လုပ်သားက MCBR အပလီကေးရှင်းကို သုံးသလား၊ ကာဗွန်လက်စ်ငှက်ဖျားလချုပ်ကို သုံးသလား? |  |
| 2.2 | လူနာတစ်ယောက်ကို ငှက်ဖျားပိုးစစ်ဆေးပြီးတာနဲ့ ကာဗွန်လက်စ် ငှက်ဖျားလချုပ်မှာ စာရင်းသွင်း ပြီးတာကြားမှာ အချိန်ဘယ်လောက် ကွာသလဲ? |  |
| 2.3 | လူနာတစ်ယောက်ကို ငှက်ဖျားပိုးစစ်ဆေး ပြီးတာနဲ့ MCBR အပလီကေးရှင်းမှာ စာရင်းသွင်း ပြီးတာကြားမှာ အချိန်ဘယ်လောက် ကွာသလဲ? |  |
| 2.4 | ငှက်ဖျားလူနာကိုစာရင်းသွင်းပြီး အဲဒီသတင်းအချက်အလက်ကို သက်ဆိုင်ရာအဖွဲ့အစည်းဆီပို့ဖို့ အချိန်ဘယ်လောက်ကြာသလဲ (မိမိထံမှ ပေးပို့ရန်)? |  |
| **Section 3: Workflow observations** | | |
| 3.1 | လူနာက လူထုအခြေပြု ဘက်စုံစေတနာ့ဝန်ထမ်း (ငှက်ဖျား)လုပ်သားကို ဘယ်လိုဆက်သွယ်သလဲ? |  |
| 3.2 | လူထုအခြေပြု ဘက်စုံစေတနာ့ဝန်ထမ်း (ငှက်ဖျား)လုပ်သားက ငှက်ဖျားပိုး စစ်ဆေးတာကို ဘယ်နေရာမှာလုပ်သလဲ? |  |
| 3.3 | လူထုအခြေပြု ဘက်စုံစေတနာ့ဝန်ထမ်း (ငှက်ဖျား)လုပ်သားက ဘာဝန်ဆောင်မှုတွေပေးလဲ? |  |
| 3.4 | လူထုအခြေပြု ဘက်စုံစေတနာ့ဝန်ထမ်း (ငှက်ဖျား)လုပ်သားက ညွှန်းပို့တဲ့ လူနာရှိလား? ရှိတယ်ဆိုရင် ဘယ်ကိုညွှန်းပို့သလဲ? |  |
| 3.5 | နေ့တစ်နေ့ရဲ့အလုပ်ချိန်ကို လူထုအခြေပြု ဘက်စုံစေတနာ့ဝန်ထမ်း (ငှက်ဖျား)လုပ်သားက ဘယ်လိုအစီအစဉ်ချထားလဲ? လေ့လာကြည့်ရှု နေစဉ်တလျောက်လုံး ၎င်းက ဘာတွေလုပ်ခဲ့သလဲ? |  |
| 3.6 | ယေဘူယျအားဖြင့် လူထုအခြေပြု ဘက်စုံစေတနာ့ဝန်ထမ်း (ငှက်ဖျား)လုပ်သားက လူနာတစ်ယောက်ကို အချိန်ဘယ်လောက်ပေးသလဲ? |  |
| 3.7 | ငှက်ဖျားစစ်ဆေးတာနဲ့ပတ်သက်တဲ့ အချက်အလက်တွေကို ဘယ်မှာရေးမှတ် သလဲ? (ကာဗွန်လက်စ်ငှက်ဖျားလချုပ်၊ MCBR၊ အခြား) |  |
| 3.8 | လူထုအခြေပြု ဘက်စုံစေတနာ့ဝန်ထမ်း (ငှက်ဖျား)လုပ်သားက အချက်အလက်တွေကို ဘယ်အချိန်မှာ ရေးမှတ်/ဖြည့်သွင်းသလဲ? |  |
| 3.9 | လူနာမရှိတဲ့အချိန်မှာ လူထုအခြေပြု ဘက်စုံစေတနာ့ဝန်ထမ်း (ငှက်ဖျား)လုပ်သားက ဘာအလုပ်တွေ လုပ်နေသလဲ? |  |
| 3.10 | တစ်နေ့တာအတွင်းကြုံတွေ့ရတဲ့ စေတနာ့ဝန်ထမ်းလုပ်ငန်းနဲ့ ပတ်သက်တဲ့ အခက်အခဲတွေကို လူထုအခြေပြု ဘက်စုံ စေတနာ့ဝန်ထမ်း (ငှက်ဖျား)လုပ်သားက ဘယ်လိုဖြေရှင်းသလဲ? |  |
| 3.11 | လုပ်ငန်းအသွားအလာ၊ အစီအစဉ်ချခြင်း၊ သတင်းပို့ခြင်း စတာတွေမှာ ပုံမှန်စီစဉ်ထားတာကနေ သွေဖည်သွားတာမျိုးရှိသလား? |  |
| 3.12 | လူထုအခြေပြု ဘက်စုံစေတနာ့ဝန်ထမ်း (ငှက်ဖျား)လုပ်သားအတွက် ဘယ်အလုပ်တွေက အချိန်အကုန်ဆုံးလဲ? |  |
| **Section 4: Facilitators and barriers in use of the MCBR** | | |
| 4.1 | MCBR (သို့မဟုတ်) ကာဗွန်လက်စ်ငှက်ဖျားလချုပ်ကို ကောင်းစွာအသုံးပြု နိုင်ဖို့ လူထုအခြေပြု ဘက်စုံစေတနာ့ဝန်ထမ်း (ငှက်ဖျား)လုပ်သား အနေနဲ့ ထူးထူးခြားခြားအသုံးပြုနေတဲ့ နည်းလမ်းကောင်းများရှိသလား? |  |
| 4.2 | လူထုအခြေပြု ဘက်စုံစေတနာ့ဝန်ထမ်း (ငှက်ဖျား)လုပ်သားက MCBR (သို့မဟုတ်) ကာဗွန်လက်စ်ငှက်ဖျားလချုပ်အသုံးပြုရာမှာ အခက်အခဲ တစ်စုံတစ်ရာရှိသလား? |  |
| 4.3 | လူထုအခြေပြု ဘက်စုံစေတနာ့ဝန်ထမ်း (ငှက်ဖျား)လုပ်သားသည် အင်တာနက်အသုံးပြုနိုင်ပါသလား? |  |
| 4.4 | MCBR အပလီကေးရှင်းက အလုပ်လုပ်သလား? သတင်းအချက်အလက် တွေကို ပို့လွှတ်သလား? |  |
| **Section 5: Stock management of medicine and supplies** | | |
| 5.1 | ဆေးနှင့်ဆေးပစ္စည်းပြတ်လပ်မှုကို လူထုအခြေပြု ဘက်စုံစေတနာ့ဝန်ထမ်း (ငှက်ဖျား) လုပ်သားတစ်ယောက်က ဘယ်လိုသတင်းပို့သလဲ? |  |
| 5.2 | သူတို့မှာ အခက်အခဲတစ်စုံတစ်ရာရှိသလား? |  |
| **Section 6: Interaction with supervisors (NMCP staff, BHSs and IP staff) regarding malaria case reporting (ကြည့်ရှုလေ့လာရန်အခွင့်ကြုံခဲ့ပါက)** | | |
| 6.1 | လူထုအခြေပြု ဘက်စုံစေတနာ့ဝန်ထမ်း (ငှက်ဖျား)လုပ်သားသည် ၎င်း၏ ကြီးကြပ်သူ(များ)နှင့် တွေ့ဆုံပါသလား? |  |
| 6.2 | ဘာအကြောင်းအရာတွေ ဆွေးနွေးကြသလဲ? |  |
| 6.3 | MCBR (သို့မဟုတ်) PBR စနစ်နဲ့ ပတ်သက်ပြီး ဆွေးနွေးကြသလား? |  |
| 6.4 | MCBR (သို့မဟုတ်) PBR အတွက် ကြီးကြပ်သူက ဘယ်လိုအထောက်အပံ့တွေ ပေးသလဲ? |  |

**Appendix I: Fieldwork Observation Guide: Stakeholders**

| **Background Information** | | |
| --- | --- | --- |
| 0.1 | Name of observer |  |
| 0.4 | Date (DD/MM/YYYY): |  |
| 0.5 | Start time: |  |
| 0.6 | End time: |  |
| **Section 1: Responsibilities and scope of work** | | |
| 1.1 | Organisation at which observation is taking place  (Choose one from the list) | SC SR / HPA / IOM / NMCP / SC PR |
| 1.2 | Number of stakeholder observed for this occasion (ideally it will be 1 stakeholder per observation) |  |
| 1.3 | Level at which observation is taking place  (Choose one from the list) | Local (township and district) / state and regional / national and central |
| 1.4 | Positions of stakeholders observed | Data, monitoring and evaluation staff / program implementation or field staff / field manager / national level program manager or director / PR level staff |
| **Section 2: Day to day operations, interactions and experiences in malaria program** | | |
| 2.1 | How does the stakeholder access and compile the data gathered by PBR and / or MCBR? | PBR  MCBR |
| 2.2 | Where and how the stakeholder store the data collected | PBR  MCBR |
| 2.3 | How does the stakeholder check, manage, clean and process the data collected? | PBR (data completeness, accuracy, etc)  MCBR (data completeness, accuracy, etc) |
| 2.4 | How does the stakeholder analyse the data collected? | PBR  MCBR |
| 2.5 | How does the stakeholder use, apply, report and provide feedback (to the data collectors) the data collected? | PBR   - Data usage in program management - Reporting to WHO and other relevant organizations - Providing feedback to ICMVs and field staff   MCBR   - Data usage in program management - Reporting to WHO and other relevant organizations - Providing feedback to ICMVs and field staff |
| **Section 3: facilitators and barriers in use of the MCBR** | | |
| 3.1 | Are there any difficulties in accessing and compilation of the PBR and MCBR data? How does the stakeholder manage any difficulties that arise in accessing the data? | Barriers  Solutions |
| 3.2 | Are there any tip/ facilitator in accessing and compilation of the PBR and MCBR data? | Facilitator |
| 3.3 | Are there any difficulties in storage of the PBR and MCBR data? How does the stakeholder manage any difficulties that arise in storage the data? | Barriers  Solutions |
| 3.4 | Are there any tip/ facilitator in storage of the PBR and MCBR data? | Facilitator |
| 3.5 | Are there any difficulties in checking, management, cleaning and processing of the PBR and MCBR data? How does the stakeholder manage any difficulties that arise in checking, management, cleaning and processing the data? | Barriers  Solutions |
| 3.6 | Are there any tip/ facilitator in checking, management, cleaning and processing of the PBR and MCBR data? | Facilitator |
| 3.7 | Are there any difficulties in analysing of the PBR and MCBR data? How does the stakeholder manage any difficulties that arise in analysing the data? | Barriers  Solutions |
| 3.8 | Are there any tip/ facilitator in analysing of the PBR and MCBR data? | Facilitator |
| 3.9 | Are there any difficulties in usage, application, reporting and providing feedback (to the data collectors) of the PBR and MCBR data? How does the stakeholder manage any difficulties that arise in usage, application, reporting and providing feedback about the data? | Barriers  Solutions |
| 3.10 | Are there any tip/ facilitator in usage, application, reporting and providing feedback (to the data collectors) of the PBR and MCBR data? | Facilitator |
| **Section 4: Experiences with MBCR application within the field environments** | | |
| **4.1** | What are the resources and predetermined conditions needed for well-functioning of MCBR reporting channel? | Predetermined conditions/ prerequisites  Resources |
| **4.2** | Were there any factors that made the use of MCBR easier for the stakeholder? | Factors |
| 4.3 | Does the stakeholder interact with any superiors and/or colleagues? How do they do this? What is discussed? | Supervisor/colleagues  Discussion points |
| **Section 5: Firsthand experience of data usage by different stakeholders** | | |
| **5.1** | Practical example of data usage from MCBR | Stakeholder 1   - Example 1 - Example 2   Stakeholder 2   - Example 1 - Example 2 |
| **5.2** | Practical example of data usage from PBR | Stakeholder 1   - Example 1 - Example 2   Stakeholder 2   - Example 1 - Example 2 |

**ငှက်ဖျားရောဂါဆိုင်ရာ သတင်းအချက်အလက်များပေးပို့ရာတွင် မိုဘိုင်းအပလီကေရှင်း (MCBR) သုံးခြင်းနှင့် ကာဗွန်လက်စ် ငှက်ဖျားလချုပ်များသုံးခြင်း (PBR) တို့၏ ထိရောက်မှုကို နှိုင်းယှဉ်လေ့လာခြင်း**

**ကျန်းမာရေးနှင့်အားကစားဝန်ကြီးဌာနမှအရာရှိများနှင့် မိတ်ဘက်အဖွဲ့အစည်းများမှ ဝန်ထမ်းများအား ကွင်းဆင်းလေ့လာကြည့်ရှုခြင်းလမ်းညွှန်**

| **အခြေခံအချက်အလက်များ** | | | |
| --- | --- | --- | --- |
| 0.1 | လေ့လာကြည့်ရှုသူ အမည် | |  |
| 0.4 | ရက်စွဲ (ရက်/လ/နှစ်) | |  |
| 0.5 | စတင်ချိန် | |  |
| 0.6 | ပြီးဆုံးချိန် | |  |
| **Section 1: Responsibilities and scope of work** | | | |
| 1.1 | | ကွင်းဆင်းလေ့လာကြည့်ရှုသည့် အဖွဲ့အစည်းအမည်  (တစ်ခုသာရွေးပါ) | SC SR / HPA / IOM / NMCP / SC PR |
| 1.2 | | လေ့လာကြည့်ရှုသည့် အရာရှိ/ဝန်ထမ်း အရေအတွက်  (ပုံမှန်အားဖြင့်လေ့လာကြည့်ရှုမှုတစ်ခုလျှင်အရာရှိ/ဝန်ထမ်းတစ်ဦး ဖြစ်ရမည်။) |  |
| 1.3 | | လေ့လာကြည့်ရှုသည့် လုပ္ငန္းခြင္ နေရာအဆင့်  Level at which observation is taking place  (Choose one from the list) | မြို့နယ်/ခရိုင်အဆင့်  တိုင်းဒေသကြီး/ပြည်နယ် အဆင့်  ဗဟိုအဆင့် |
| 1.4 | | လေ့လာကြည့်ရှုသည့် အရာရှိ/ဝန်ထမ်း၏ ရာထူးအဆင့် | Data, monitoring and evaluation staff / program implementation or field staff / field manager / national level program manager or director / PR level staff |
| Section 2: Day to day operations, interactions and experiences in malaria program | | | |
| 2.1 | | အရာရှိ/ဝန်ထမ်းသည် MCBR နှင့်  ကာဗွန်လက်စ်ငှက်ဖျားလချုပ်တို့မှ ကောက်ယူထားသောသတင်းအချက်အလက် များကို ဘယ်လို ရယူ ပေါင်းစပ် သလဲ? | PBR  MCBR |
| 2.2 | | ကောက်ယူရရှိထားတဲ့ သတင်းအချက်အလက်တွေကို ဘယ်နေရာမှာ ဘယ်လို သိမ်းဆည်းသလဲ? | PBR  MCBR |
| 2.3 | | ကောက်ယူရရှိထားတဲ့ သတင်းအချက်အလက်တွေကို ဘယ်လို စစ်ဆေးပြီး ဆက်လက်စီမံခန့်ခွဲသလဲ? | PBR (data completeness, accuracy, etc)  MCBR (data completeness, accuracy, etc) |
| 2.4 | | ကောက်ယူရရှိထားတဲ့ သတင်းအချက်အလက် တွေကို ဘယ်လို ဆန်းစစ် သလဲ? | PBR  MCBR |
| 2.5 | | ကောက်ယူရရှိထားတဲ့ သတင်းအချက်အလက်တွေကို လက်တွေ့ ဘယ်လို အသုံးချသလဲ? ဘယ်လိုဆက်ပြီး သတင်းပို့သလဲ? သတင်းအချက်အလက် ကောက်တဲ့သူတွေကို ဘယ်လိုပြန်ပြီး တုံ့ပြန်မှုပေးသလဲ? | PBR  Data usage in program management  Reporting to WHO and other relevant organizations  Providing feedback to ICMVs and field staff  MCBR  Data usage in program management  Reporting to WHO and other relevant organizations  Providing feedback to ICMVs and field staff |
| Section 3: facilitators and barriers in use of the MCBR | | | |
| 3.1 | | MCBR နဲ့ ကာဗွန်လက်စ်ငှက်ဖျားလချုပ်တို့နဲ့  ကောက်ယူရရှိထားတဲ့  သတင်းအချက်အလက်တွေကို  ရယူပေါင်းစည်းရာမှာ အခက်အခဲ တစ်စုံတစ်ရာ  ရှိသလား?  ရှိတယ်ဆိုရင် အဲဒီအခက်အခဲကို ဘယ်လိုစီမံခန့်ခွဲပြီး  ဖြေရှင်းခဲ့သလဲ? | အခက်အခဲ  ဖြေရှင်းချက် |
| 3.2 | | PBR နှင့် MCBR တို့ဖြင့်  ကောက်ယူထားသော  သတင်းအချက်အလက်တွေကို  ရယူရာ၌လည်းကောင်း၊  စုပေါင်းပြုစုရာ၌လည်းကောင်း  အထောက် အပံ့ဖြစ်စေသော  နည်းလမ်းကောင်းများရှိသလား? | နည်းလမ်းကောင်းများ |
| 3.3 | | MCBR နဲ့ ကာဗွန်လက်စ်ငှက်ဖျားလချုပ်တို့နဲ့  ကောက်ယူရရှိထားတဲ့  သတင်းအချက်အလက်တွေကို  သိမ်းဆည်းရာမှာ အခက်အခဲ တစ်စုံတစ်ရာ ရှိသလား?  ရှိတယ်ဆိုရင် အဲဒီအခက်အခဲကို  ဘယ်လိုစီမံခန့်ခွဲပြီး ဖြေရှင်းခဲ့သလဲ? | အခက်အခဲ  ဖြေရှင်းချက် |
| 3.4 | | PBR နှင့် MCBR တို့ဖြင့်  ကောက်ယူထားသော  သတင်း အချက်အလက်တွေကို  သိမ်းဆည်းရာမှာ အထောက်အပံ့  ဖြစ်စေသော နည်းလမ်းကောင်း များ ရှိသလား? | နည်းလမ်းကောင်းများ |
| 3.5 | | MCBR နဲ့ ကာဗွန်လက်စ်ငှက်ဖျားလချုပ်တို့နဲ့  ကောက်ယူရရှိထားတဲ့  သတင်းအချက်အလက်တွေကို  စစ်ဆေးပြီး ဆက်လက်စီမံခန့်ခွဲရာမှာ  အခက်အခဲ တစ်စုံတစ်ရာ ရှိသလား?  ရှိတယ်ဆိုရင် အဲဒီအခက်အခဲကို  ဘယ်လိုစီမံခန့်ခွဲပြီး ဖြေရှင်းခဲ့သလဲ? | အခက်အခဲ  ဖြေရှင်းချက် |
| 3.6 | | PBR နှင့် MCBR တို့ဖြင့်  ကောက်ယူထားသော  သတင်းအချက်အလက်တွေကို  စစ်ဆေးပြီးဆက်လက်စီမံခန့်ခွဲရာမှာ  အထောက်အပံ့ဖြစ်စေသော  နည်းလမ်းကောင်းများ ရှိသလား? | နည်းလမ်းကောင်းများ |
| 3.7 | | MCBR နဲ့ ကာဗွန်လက်စ်ငှက်ဖျားလချုပ်တို့နဲ့  ကောက်ယူရရှိထားတဲ့  သတင်းအချက်အလက်တွေကို  ဆန်းစစ်ရာမှာ အခက်အခဲ တစ်စုံတစ်ရာ ရှိသလား?  ရှိတယ်ဆိုရင် အဲဒီအခက်အခဲကို  ဘယ်လိုစီမံခန့်ခွဲပြီး ဖြေရှင်းခဲ့သလဲ? | အခက်အခဲ  ဖြေရှင်းချက် |
| 3.8 | | PBR နှင့် MCBR တို့ဖြင့်  ကောက်ယူထားသော  သတင်းအချက်အလက်တွေကို  ဆန်းစစ်ရာမှာ အထောက်အပံ့ ဖြစ်စေသော  နည်းလမ်းကောင်းများ ရှိသလား? | နည်းလမ်းကောင်းများ |
| 3.9 | | MCBR နဲ့ ကာဗွန်လက်စ်ငှက်ဖျားလချုပ်တို့နဲ့  ကောက်ယူရရှိထားတဲ့  သတင်းအချက်အလက်တွေကို  လက်တွေ့အသုံးချ၊ သတင်းပို့၊  တုံ့ပြန်မှုပေး ရာမှာ အခက်အခဲ တစ်စုံတစ်ရာ ရှိသလား?  ရှိတယ်ဆိုရင် အဲဒီအခက်အခဲကို  ဘယ်လိုစီမံခန့်ခွဲပြီး ဖြေရှင်းခဲ့သလဲ? | အခက်အခဲ  ဖြေရှင်းချက် |
| 3.10 | | PBR နှင့် MCBR တို့ဖြင့်  ကောက်ယူထားသော  သတင်းအချက်အလက်တွေကို  လက်တွေ့အသုံးချ၊ သတင်းပို့၊  တုံ့ပြန်မှုပေးရာမှာ အထောက်အပံ့ဖြစ်စေသော  နည်းလမ်းကောင်း များ ရှိသလား? | နည်းလမ်းကောင်းများ |
| Section 4: Experiences with MBCR application within the field environments | | | |
| 4.1 | | MCBR ဖြင့်သတင်းပို့ခြင်းဖြစ်စဉ်  ကောင်းမွန်စွာလည်ပတ်နိုင်စေရန်အတွက်  လိုအပ်သော အရင်းအမြစ်များနှင့်  ရှိထားရမည့် အခြေအနေများက ဘာတွေလဲ? | Predetermined conditions/ prerequisites  Resources |
| 4.2 | | MCBR အသုံးပြုရာမှာ ပိုမိုလွယ်ကူစေတဲ့  အချက်တွေက ဘာတွေလဲ? | Factors |
| 4.3 | | လေ့လာကြည့်ရှုသည့်အရာရှိ/ဝန်ထမ်းသည်  သူ၏ကြီးကြပ်သူများ၊ အခြားလုပ်ဖော်ကိုင်ဘက်များနှင့်  အပြန်အလှန်ဆက်ဆံတာမျိုးရှိသလား?  ရှိတယ် ဆိုရင် ဘယ်လိုမျိုးလုပ်သလဲ?  ဘာတွေ ဆွေးနွေးကြသလဲ? | Supervisor/colleagues  Discussion points |
| Section 5: Firsthand experience of data usage by different stakeholders | | | |
| 5.1 | | MCBR ဖြင့်ကောက်ယူရရှိထားသော  သတင်းအချက်အလက်များအသုံးချခြင်း  လက်တွေ့ဥပမာများ  (အကယ်၍ ရှိပါက) | Stakeholder 1  Example 1  Example 2  Stakeholder 2  Example 1  Example 2 |
| 5.2 | | ကာဗွန်လက်စ်ငှက်ဖျားလချုပ်ဖြင့်  ကောက်ယူရရှိထားသော  သတင်းအချက် အလက်များအသုံးချခြင်း  လက်တွေ့ဥပမာများ  (အကယ်၍ ရှိပါက) | Stakeholder 1  Example 1  Example 2  Stakeholder 2  Example 1  Example 2 |
