# Supplementary material for: A mobile phone application for malaria case-based reporting to advance malaria surveillance in Myanmar: a mixed methods evaluation
Source: Malar J. 2021 Mar 26;20:167. doi: 10.1186/s12936-021-03701-6 (PMC7995396; doi:10.1186/s12936-021-03701-6)
Supplement: Supplementary file 7 — Additional file 7. Data collection tools. [file 12936_2021_3701_MOESM7_ESM.docx]

***Additional methods***

**Infrastructure to support intervention**

Electricity

Nearly all ICMVs (98%) had access to electricity in their villages, with solar electricity being the most common source. In some remote villages where not all households have solar panels, ICMVs sometimes had to go to other homes to charge their phone. Some use power banks but the power banks also have to be charged. Some ICMVs have to run a generator to charge their phones.

Mobile phone network and internet coverage

In Myanmar the available mobile networks are MPT, Telenor, Ooredoo and MyTel. Mobile phone network coverage was available in 93% villages where this information was collected. Although the mobile phone network coverage was not satisfactory back in 2014, 2015 and 2016, it improved through 2017 and 2018, and it is possible to make a phone call in almost all places. In some remote areas the mobile network strength is still weak and internet access is not possible. For malaria reporting using MCBR application, internet access is an essential requirement for uploading the entered patient records into the DHIS2 database by the ICMVs. For the ICMVs, internet access is achieved through mobile phone data network which can be accessed by their mobile phones. For accessing internet through mobile phones, the ICMVs are supported with mobile phone bills by the supporting organizations.

**Secondary data analysis of malaria test report data**

Excel spreadsheets containing 2018 malaria testing report data collected through the MCBR application and the PBR system, from the NMCP and HPA, IOM, and SCI, were merged into a single dataset in Stata (version 15.1). Each row in the dataset represented a “case” whereby an individual had been given a RDT by an ICMV and information was collected pertaining to their age, sex, pregnancy status, RDT result, and antimalarials given. Following completion of MCBR training, ICMVs were instructed to use both the paper-based register (PBR system) and the MCBR application to enter the requisite details of every malaria test they conducted. Thus, every malaria test conducted by MCBR-trained ICMVs in 2018 should have an PBR record and an MCBR record. PBR malaria testing records were excluded from analysis if the test date preceded the ICMV’s MCBR training date. The number and percentage of complete MCBR and PBR records for each of the following data fields was determined: date of test, patient age, patient sex, patient address, pregnancy status (among females), pregnancy status (among females aged ≥15 years), and RDT result. As an overall measure of completeness, the number of records with complete entries across five key fields (date of test, patient age, patient sex, patient address, and RDT result) was determined. The total number of RDT positive results was determined for each reporting system, and the number and percentage of complete fields specific to these malaria positive results was recorded (complicated/uncomplicated malaria status, antimalarial given, referral recorded). Chi-squared tests were performed to determine whether there was a statistically significant difference in the percentage of completeness between MCBR and PBR records. The number of volunteers with unique identifiers using each system was recorded, and the number of notifications received by each volunteer was determined. The use of distinct volunteer identification systems in the PBR and MCBR system precluded matching of individual records between the two systems.

**ICMV questionnaires**

Sampling method

A two-stage cluster sampling approach was used to sample a target of 200 ICMVs in 37 township clusters across four strata (IOM, HPA, SC-SR and NMCP). At the first stage, across each of the four partner strata, a total of n=8 township clusters were randomly selected (with replacement) with probability proportionate to their ICMV population size (PPS) to ensure larger township clusters were well represented (n=2 clusters per stratum) and to reduce standard error. For HPA, one cluster was selected twice due to the PPS sampling with replacement approach. At the second stage and in order to provide equal selection probabilities at the ICMV level (these become unequal after PPS), a fixed sample of n=25 ICMVs were randomly selected from each of the selected township clusters to participate in the survey (Additional table M1). At this second stage, an inflated number of ICMVs in each cluster was invited to participate in the survey given an expected refusal/non-response rate of 5% (i.e. ~30 per cluster).

A list of selected ICMVs was shared with respective organizations and their eligibility was checked to ensure they were aged 18 years or over and had experience as an ICMV using both the MCBR application and PBR. Due to a lower than expected number of eligible ICMVs, a sample of 165 ICMVs was eventually obtained (Supplementary table 3). Questionnaire responses from two ICMV (one each from HPA and NMCP) were excluded from analyses because at the time of surveying these ICMV had undergone MCBR training but had not yet commenced using the application in their villages.

Questionnaire administration

For IOM and NMCP (Kyaukse and Thazi cluster), surveyors attended the monthly meetings and ICMV refresher training sessions where the respective organizations gathered their ICMVs in their relevant townships. For SC-SR, HPA and NMCP (Chaungzon cluster), selected ICMVs were informed and contacted by their respective organizations one week before the survey was undertaken. These ICMVs were then invited to the relevant townships where a data collection team from Burnet Institute Myanmar visited and executed the survey. Prior to commencing the questionnaire, prospective participants read through and signed a Participant Information and Consent Form outlining the scope of the study, the risks and benefits of participating, the study procedures and their role in the study. Each survey was conducted in a private space by a surveyor who recorded the participants’ answers on the provided questionnaire. Surveys were conducted in Burmese translated into English by researchers at Burnet Institute Myanmar. Each participant was provided with 4000 MMK^[[1]](#footnote-1)^ to compensate for time spent answering the survey questions (approximately 45-60 minutes).

Analysis of questionnaire responses

Data entry was performed in Microsoft Excel and 20% of the data was randomly selected and checked against the primary data source (survey questionnaire) for data validity. Data management and analysis was performed in Stata version 15.0 (StataCorp, Texas, USA). In order to provide unbiased point estimates where the achieved cluster sizes from PPS sampling vary (i.e. refusal/non-response), inverse proportional sampling weights were derived and applied in all statistical analyses. Further, appropriate variance estimation which accounts for the complex cluster sampling approach were used in all statistical analyses to provide correct inference (i.e. 95% confidences intervals and probability values). Categorical survey responses are presented as counts and per cent (%) for each response category.

**Focus group discussions, key informant Interviews and in-depth Interviews**

Participant recruitment

Qualitative data collection methods included semi-structured interviews (Key Informant Interviews, KII and In-depth interviews, IDI) for key stakeholders and Focus Group Discussions (FGD) with ICMVs were conducted in Kachin, Kayin and Mon States, Mandalay, Sagaing and Yangon Regions and Naypyidaw City of Myanmar at a combination of national, state/regional, township and community levels, and engaged ICMVs and other key stakeholders including representatives from the Myanmar Ministry of Health and Sports (MoHS) and other malaria programme Implementing Partners (IPs).

FGD were conducted with a total of 83 ICMVs (male: 38, female: 45), aged 18 years or over, to explore opinions on data collection using the MCBR and PBR systems. The FGD were conducted in groups of 6-8, separated by gender and managing IP, and facilitated by two in-country research team members. The FGDs were conducted in either Burmese or local dialect and took 1-2 hours.

Semi-structured interviews, including 12 in-depth interviews (IDI) and two key informant interviews (KII), were conducted with Myanmar Ministry of Health and Sports (MoHS) and IP stakeholders responsible for managing malaria reporting data. Participants were purposively recruited based on their role in the organization/department, interest, experience with MCBR application, and the operational feasibility of the interview. Interviews were conducted in-person by a trained interviewer or researcher in Burmese (except one interview in English). Interviews took approximately 45-60 minutes to complete. Locations were selected in consideration of potential sensitivities and risks relevant to each participant and included participants’ workplaces and social meeting points with privacy (e.g. tea house, restaurant). Potential participants were informed that audio and written recordings of interviews would be taken and that they could refuse to participate if they did not want to be recorded. Individuals were given at least 1-2 days to decide if they wish to participate and informed that they may withdraw from the study at any time.

FGD participants were provided with refreshments and 4,000 MMK to compensate for their time. Interviewees were provided with refreshments and a small gift such as an umbrella, towel or cap, to the value of no more than 6000 MMK to acknowledge time spent in interviews.

Data management and analysis

FGDs and interviews were audio recorded and field notes were taken with the informed consent of the participants. Each interview participant was assigned a unique code linked to his or her data and no identifying information was stored with the collected data. Voice recorders used to take audio-recordings were kept in a locked box in the field before the data was transferred into a password-protected computer and were only accessible to the investigators. The audio records were transcribed verbatim and translated into English. Translated data were organized, managed and analysed in Nvivo (version 12) by two researchers.

Inductive thematic analysis was used to analyse the qualitative data with process involving the steps of data immersion, coding, categorisation/sub-theme development and major theme development, guided by the collected data via an in-depth code guide. Emerging themes during the data collection were captured and incorporated into the thematic framework in data analysis stage. The level of analysis was mainly surface level and explored patterns and new understandings relating to the perspectives and experiences of the participants. One investigator analysed all the data and another investigator randomly extracted 10% of the data and performed an independent analysis. Afterwards, both investigators discussed the themes and subthemes and reached a consensus.

A phenomenological approach was used to explore the experiences of the stakeholders in data accessing, policy making and program implementation as well as in the exploration of opinions and knowledge of ICMVs and stakeholders. Triangulation of the data from KIIs, IDIs, workshops and FGDs during analysis and reporting strengthened the validity of the findings.

**Field observations of ICMVs**

Researchers conducted field observations of ICMVs who were using both the PBR and MCBR systems to report malaria tests in their villages. Forty-four ICMVs were recruited to participate in field observations: Six ICMVs from each of seven townships (Bilin, Thaton, Homelin, Hlaing Bwe, Waingmaw, Chaungsone, Tharsi), plus an additional two ICMVs. ICMVs were selected purposively to ensure representation across genders and representation of those with high and low annual blood examination rates (ABER) in 2018. All ICMVs in each township were ranked according to ABER and the mean ABER of that township was calculated. ICMVs whose ABERs were above the mean ABER were classified as high performers and those below mean ABER were classified as low performers. Three high-performing ICMVs and the three low-performing ICMVs were selected from each township. At least one male and one female ICMV was included in both high performer and low performer groups. ICMVs from villages that were difficult for researchers to access due to security or logistical reasons were excluded. A list of the selected ICMVs for each township was provided by the researchers to the respective supervisors of NMCP and IPs in order to confirm that the selected ICMVs were available to participate in the observations. The ICMVs were recruited via phone or in-person by the respective township focal person (supervisors) approximately two weeks before the observation. The ICMV observations were conducted in seven townships according to observational field guides (Additional file 7)) by BI investigators from October 2019 to January 2020. Each observation lasted approximately 2 hours and involved one researcher observing the daily work activities of one ICMV. The observer used the observational field guide to take notes throughout the session. Focus areas of the observations included demographic factors such as infrastructure of the villages, day to day operations, interactions and experiences in malaria program, facilitators and barriers to use of the MCBR, experience with the PBR system and MBCR application within the field environments, and descriptions of the PBR system and MCBR application. These focus areas were recorded by a combination of observations of the village itself, direct observation of the ICMV, and inspection of records and documents including the following: malaria case registers, stock and health education record books, notes for recording and reporting, monthly and quarterly reports and MCBR records and documents relevant to the evaluation of malaria PBR or MCBR surveillance in Myanmar.

**Field Office observations**

Researchers visited the field offices of five field managers and data monitoring and evaluation (M&E) staff from IPs and MoHS who are responsible for malaria test reporting data. Researchers observed the entry, compilation, and management of malaria case data and the submission of consolidated reports. Offices included a state level office of IOM, township level offices of SC-SR and district and state level offices of NMCP. An average field office observation took 3-4 hours. Researchers used an observational field guides (Additional file 7) to take notes whilst observing the daily work activity of the ICMV for approximately two hours and to record notes in offices on the entry, compilation, and management of malaria case data and the submission of consolidated reports, with observations taking 3-4 hours, on average. In offices, researchers also inspected monthly and quarterly reports and MCBR records and documents relevant to the evaluation of malaria PBR or MCBR surveillance in Myanmar.

**Cost analysis**

A program experience approach was used to estimate the cost of development, implementation, and roll-out of the MCBR system compared to ongoing costs of running the PBR system. All one-off costs and ongoing costs associated with each reporting system were itemized in an Excel spreadsheet using expenditure reports provided by SC-PR (for costs associated with SC-PR, IOM, SC-SR and HPA programs) and UNOPS-PR (for costs associated with NMCP program).

An estimation of the cost of future implementation of the MCBR system was determined based on the cost of the initial roll-out conducted by SC-PR, minus any components related to the piloting process. Both the implementation costs and ongoing costs were multiplied by a geographical scaling factor calculated by dividing the estimated number of villages serviced by ICMV nationally in Myanmar (20,000) by the number of villages covered by the MCBR roll-out to 2019 (2488). The total implementation costs for MCBR and PBR were calculated for a model of 2,488 ICMVs in a year, 20,000 ICMVs of nationwide implementation in a year, and for 3 years implementation with 20,000 ICMVs nationwide. The total cost of implementing the MCBR system was compared over time to the total cost estimated for the PBR system. This calculation was based on the assumption that MCBR would replace PBR in all villages currently serviced by ICMV using PBR and did not include any estimation of the cost of expanding MCBR to areas not yet serviced by ICMV. It was also assumed that one-off costs such as MCBR software development because one-off resources such as MCBR software development and MCBR server could be shared among all ICMVs.

Any costs that were not contained directly in expenditure reports (e.g. due to integration / reliance on existing services) were identified by the project team, and were either estimated / extracted from other budgets in consultation with SC-PR, UNOPS and NMCP, or determined based on logical assumptions in consultation with UNOPS-PR, SC-PR and NMCP.

The cost per case of reporting using each system was calculated by dividing the total implementation and annual operational costs for 2488 ICMVs by the number of tests conducted in the geographical area covered by those ICMVs in the year 2018 (48,922 tests and 106,743 tests for MCBR and PBR, respectively).

All costs were calculated in United States Dollar ($) and expenditure in Myanmar Kyats were converted into $ using the UN exchange rate applied in 2018.

1. Exchange rate was 1 USD ≈ 1,520 MMK at the time of surveying (Ref: UN exchange rate). [↑](#footnote-ref-1)
