# Supplementary material for: A mobile phone application for malaria case-based reporting to advance malaria surveillance in Myanmar: a mixed methods evaluation
Source: Malar J. 2021 Mar 26;20:167. doi: 10.1186/s12936-021-03701-6 (PMC7995396; doi:10.1186/s12936-021-03701-6)
Supplement: Supplementary file 8 — Additional file 8. Diagram of malaria case data flow. [file 12936_2021_3701_MOESM8_ESM.pdf]

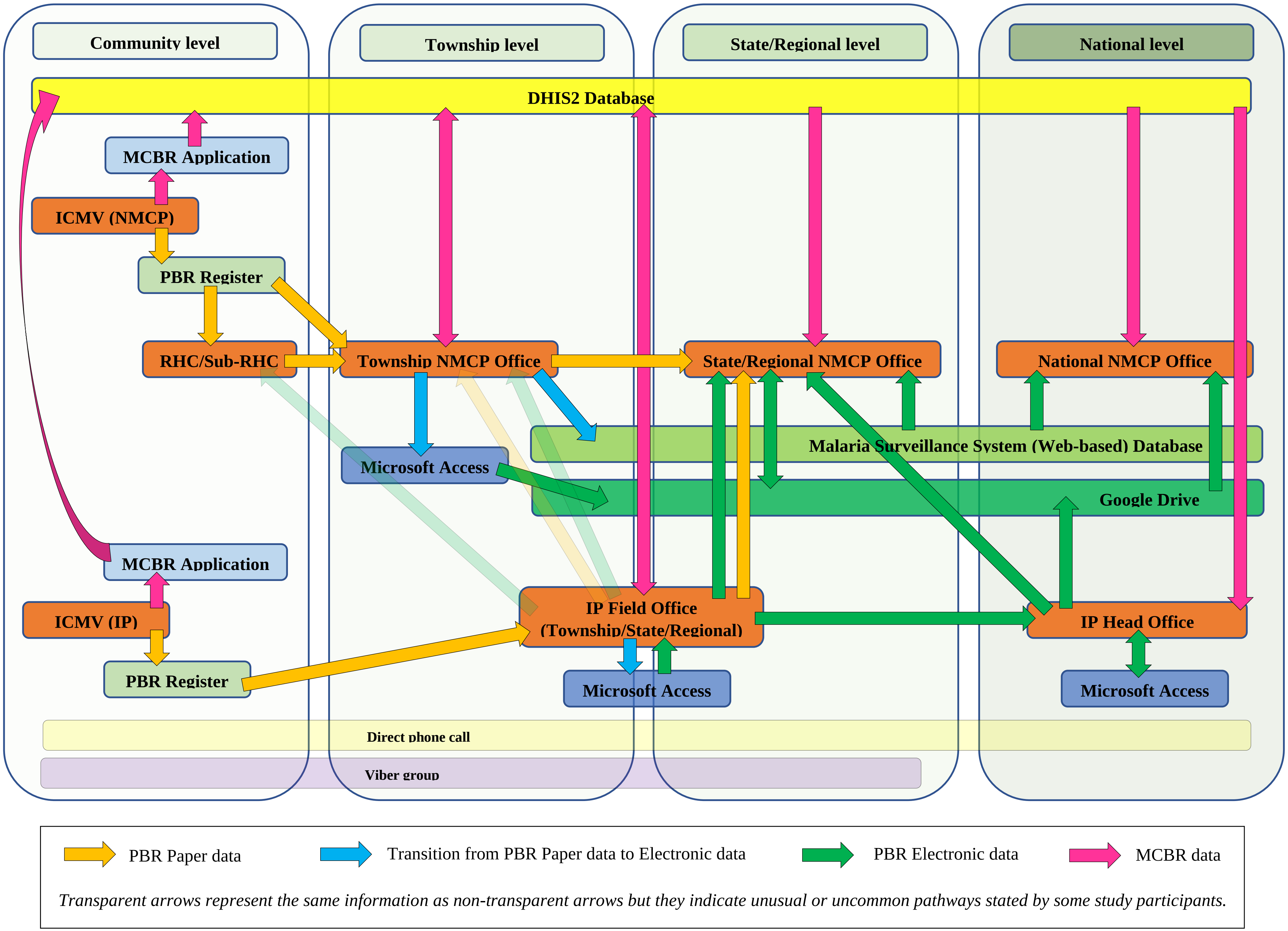
Community level
Township level
State/Regional level
National level
DHIS2 Database
MCBR Application
ICMV (NMCP)
PBR Register
RHC/Sub-RHC
Township NMCP Office
State/Regional NMCP Office
National NMCP Office
Malaria Surveillance System (Web-based) Database
Microsoft Access
Google Drive
MCBR Application
ICMV (IP)
PBR Register
IP Field Office
(Township/State/Regional)
IP Head Office
Microsoft Access
Microsoft Access
Direct phone call
Viber group
PBR Paper data
Transition from PBR Paper data to Electronic data
PBR Electronic data
MCBR data
Transparent arrows represent the same information as non-transparent arrows but they indicate unusual or uncommon pathways stated by some study participants.
