# Supplementary material for: A mobile phone application for malaria case-based reporting to advance malaria surveillance in Myanmar: a mixed methods evaluation
Source: Malar J. 2021 Mar 26;20:167. doi: 10.1186/s12936-021-03701-6 (PMC7995396; doi:10.1186/s12936-021-03701-6)
Supplement: Supplementary file 1 — Additional file 1. Carbonless register. [file 12936_2021_3701_MOESM1_ESM.pdf]

လက်ခံ

# စေတနာ့ဝန်ထမ်း ငှက်ဖျားလူနာစစ်ဆေးကုသမှုမှတ်တမ်း/လချုပ်

တိုင်းဒေသကြီး/ပြည်နယ် ---------------------- မြို့နယ် ---------------------- တိုက်နယ်/ကျေးလက်ကျန်းမာရေးဌာန ---------------------- ကျေးလက်ကျန်းမာရေးဌာနခွဲ ----------------------

အဖွဲ့အစည်းအမည် ---------------------- ကျေးရွာ ---------------------- စေတနာ့ဝန်ထမ်းအမည် ---------------------- လ ---------------------- ခုနှစ် ----------------------

| စဉ် | ရက်စွဲ | အမည် | အသက်အုပ်စု | | | | | လိပ်စာ | လိင် | | ကိုယ်ဝန်ဆောင် | RDT ဖြင့်စစ်ဆေး | | | | ဖြင့်ပ | | ကုသပေးသော ငှက်ဖျားဆေး | | | | | | ဖျားသည့်ရက် | | ဆေးရုံ/ဆေးခန်းသို့ ညွှန်းပို့ခြင်း/သေဆုံး | ငှက်ဖျားပိုးတွေ့၊ သေဆုံး | မှတ်ချက် ကူးစက်ဒေသ |
|---|---|---|---|---|---|---|---|---|---|---|---|---|---|---|---|---|---|---|---|---|---|---|---|---|---|---|---|---|
| | | | ၁ နှစ်အောက် | ၁-၄ | ၅-၉ | ၁၀-၁၄ | ၁၅ နှစ်နှင့် အထက် | | ကျား | မ | | ပိုးတွေ့ ဖယ်လ်စီပါရမ် | ပိုးတွေ့ ဗိုင်းဗက်စ်/ရောနှော | ပိုးတွေ့ ပိုးရောနှော | ပိုးမတွေ့ | သာမန် | ပြင်းထန် | ကိုအာတမ်-၂၄ | ကိုအာတမ်-၁၈ | ကိုအာတမ်-၁၂ | ကိုအာတမ်-၆ | ကလိုရိုကွင်း | ပရိုင်မာကွင်း | ၂၄ နာရီအတွင်း | ၂၄ နာရီအထက် | | | |
| ၁ | | | | | | | | | | | | | | | | | | | | | | | | | | | | |
| ၂ | | | | | | | | | | | | | | | | | | | | | | | | | | | | |
| ၃ | | | | | | | | | | | | | | | | | | | | | | | | | | | | |
| ၄ | | | | | | | | | | | | | | | | | | | | | | | | | | | | |
| ၅ | | | | | | | | | | | | | | | | | | | | | | | | | | | | |
| ၆ | | | | | | | | | | | | | | | | | | | | | | | | | | | | |
| ၇ | | | | | | | | | | | | | | | | | | | | | | | | | | | | |
| ၈ | | | | | | | | | | | | | | | | | | | | | | | | | | | | |
| ၉ | | | | | | | | | | | | | | | | | | | | | | | | | | | | |
| ၁၀ | | | | | | | | | | | | | | | | | | | | | | | | | | | | |
| ၁၁ | | | | | | | | | | | | | | | | | | | | | | | | | | | | |
| ၁၂ | | | | | | | | | | | | | | | | | | | | | | | | | | | | |
| ၁၃ | | | | | | | | | | | | | | | | | | | | | | | | | | | | |
| ၁၄ | | | | | | | | | | | | | | | | | | | | | | | | | | | | |
| ၁၅ | | | | | | | | | | | | | | | | | | | | | | | | | | | | |
| စုစုပေါင်း | | | | | | | | | | | | | | | | | | | | | | | | | | | | |

| လ | အကြောင်းအရာ | ငှက်ဖျားပိုးစစ်ကရိယာ | ကိုအာတမ်-၂၄ | ကိုအာတမ်-၁၈ | ကိုအာတမ်-၁၂ | ကိုအာတမ်-၆ | ကလိုရိုကွင်း | ပရိုင်မာကွင်း |
|---|---|---|---|---|---|---|---|---|
| | ယခင်လလက်ကျန် | | | | | | | |
| | ယခုလရရှိ | | | | | | | |
| | စုစုပေါင်း | | | | | | | |
| | ယခုလအသုံး | | | | | | | |
| | ယခုလလက်ကျန် | | | | | | | |

လက်မှတ်----------------------

အမည် ----------------------

ကျေးရွာ ----------------------

**အဖွဲ့အစည်းအမည်**
